# Anti-hepatocellular carcinoma activities of novel hydrazone derivatives *via* downregulation of interleukin-6†

**DOI:** 10.1039/d4ra05854b

**Published:** 2024-11-28

**Authors:** Ahmed Nabil, Marwa Abdel-Motaal, Ayman Hassan, Mohamed M. Elshemy, Medhat Asem, Mariam Elwan, Mitsuhiro Ebara, Mohammed Abdelmageed, Gamal Shiha, Hassan M. E. Azzazy

**Affiliations:** a Research Center for Macromolecules and Biomaterials, National Institute for Materials Science (NIMS) Tsukuba 305-0044 Japan TOLBA.AhmedNabil@nims.go.jp +201000618349; b Biotechnology and Life Sciences Department, Faculty of Postgraduate Studies for Advanced Sciences (PSAS), Beni-Suef University Beni-Suef Egypt; c Egyptian Liver Research Institute and Hospital (ELRIAH) Sherbin El Mansoura Egypt; d Department of Chemistry, College of Science, Qassim University Qassim Buraydah 51452 Saudi Arabia ma.mohammed@qu.edu.sa +966569909737; e Faculty of Science, Menoufia University Menoufia Egypt; f Department of Civil Engineering, College of Engineering and Information Technology, Onaizah Colleges Qassim Saudi Arabia; g Egyptian Ministry of Health El Mansoura Dakahlia Egypt; h Graduate School of Pure and Applied Sciences, University of Tsukuba 1-1-1 Tennodai, Tsukuba Ibaraki 305-8577 Japan; i Graduate School of Industrial Science and Technology, Tokyo University of Science 6-3-1 Niijuku Katsushika-ku Tokyo 125-8585 Japan; j Department of Pharmacology and Toxicology, Faculty of Pharmacy, Buraydah Colleges Qassim Saudi Arabia; k Hot Laboratory Center, Atomic Energy Authority Cairo Egypt; l Hepatology and Gastroenterology Unit, Internal Medicine Department, Faculty of Medicine, Mansoura University Egypt; m Department of Chemistry, School of Sciences & Engineering, The American University in Cairo New Cairo 11835 Egypt hazzazy@aucegypt.edu +201000565727

## Abstract

Hepatocellular carcinoma (HCC) is one of the leading causes of cancer-related morbidity worldwide. Sorafenib is a first-line drug for the treatment of HCC, however, it is reported to cause serious adverse effects and may lead to resistance in many patients. In this study, 20 hydrazone derivatives incorporating triazoles, pyrazolone, pyrrole, pyrrolidine, imidazoline, quinazoline, and oxadiazine moieties were designed, synthesized, and characterized. In addition to molecular docking and *in silico* ADME study, the cytotoxic activity of the synthesized compounds was evaluated against the human hepatocellular cancer cell line (HepG2) and liver mesenchymal stem cells as a normal cell line. The antitumor activities of the derivatives against sorafenib were compared. Of the 20 synthesized compounds, compound 16 demonstrated potential as a potent anti-HCC drug candidate through downregulation of interleukin 6 which reduces inflammation and tumorigenesis with a strong binding interaction and bioavailability.

## Abstract 2

A novel hydrazone derivative, compound 16, demonstrated a potent anticancer effect against hepatocellular carcinoma.

## Graphical abstract

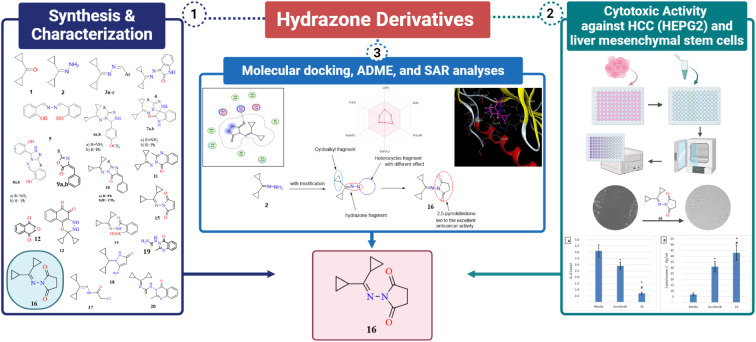

## 1. Introduction

HCC is considered the sixth most identified neoplasm and the second most common cause of cancer-related death worldwide.^1,2^ It usually occurs in patients with liver cirrhosis.^3^ The most recognized risk factors for HCC include chronic viral infections of hepatitis B and C, autoimmune hepatitis, alcohol, aflatoxin B1, non-alcoholic steatohepatitis, obesity, and diabetes mellitus.^4^

Sorafenib is a first-line drug for patients with advanced HCC. It reduces tumor cell growth and progression through inhibition of multiple serine/threonine kinases involved in tumor progression and angiogenesis.^5^ These include the vascular endothelial growth factor receptor (VEGFR-2/3), platelet-derived growth factor receptor (PDGF-R), Flt3, c-Kit, and Raf kinase.^5,6^

Although sorafenib has demonstrated clinical advantages in patients with HCC, it is reported to exert multiple side effects primarily caused by its suppression of kinases in healthy cells. These include diarrhea and dermatological effects such as hand-foot skin reactions, alopecia, stomatitis, and multiforme erythema.^7^ Other side effects include fatigue, hypertension, cardiovascular problems, hemorrhage, renal toxicity, pancreatitis, mouth ulcers, and weight loss.^8^ Furthermore, resistance to sorafenib represents a major challenge in the treatment of advanced/recurrent HCC.^9^ Therefore, there is a need to develop other agents to overcome the previous drawbacks.

Interleukin 6 (IL-6) is a key molecule of the immune response that is produced in response to infections and tissue damage.^10^ Elevated blood levels of IL-6, independent of other risk factors for HCC, have been associated with a higher risk of developing HCC.^11^ Furthermore, by promoting the repair and stimulation of countersignalling (antioxidant and anti-apoptotic/pro-survival) pathways, IL-6 protects tumor cells against DNA damage, oxidative stress, and/or apoptosis caused by anticancer treatments. Therefore, a potential therapeutic approach for cancer treatment could involve suppressing IL-6 or its signalling pathway alone or in conjunction with conventional anticancer strategies.^12^

**Fig. 1:**
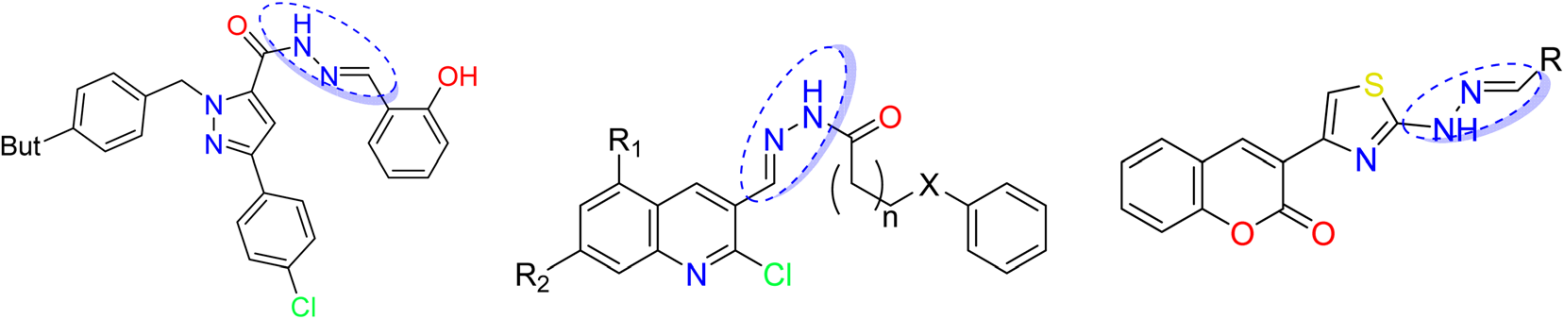
Hydrazones based on heterocycles reported showing antitumor activity.

Hydrazone derivatives represent a class of compounds containing a biologically active pharmacophore (=N–NH–R) with various biological effects^13–15^ such as antibacterial,^16–18^ anti-inflammatory,^19^ antimalarial,^20^ anticonvulsant,^21^ antidepressant, and antiproliferative^22^ activities. Different heterocycle-containing hydrazones have been reported to exert powerful cytotoxic and antitumor actions.^23,24^ Importantly, various classes of hydrazones based on coumarin, triazoles, pyridine, indole, quinolone, caffeine, pyrimidine have been evaluated for their anticancer potential against various cell lines,^25–27^ as depicted in Fig. 1. Additionally, hydrazone derivatives have shown promising therapeutic potential for the treatment of neurodegenerative diseases such as Alzheimer's disease.^28^ Hydrazone links are also used as pH-responsive drug delivery systems^29^ and in nifuroxazide, an antibiotic used for the treatment of colitis and dehydration. Consequently, these pharmacophores were used as intermediates for the synthesis of heterocycles with numerous biological activities.^30–32^

Synthesis of broad-spectrum biologically active hydrazone-based heterocycles was reported.^33^ Some of these compounds exhibited high *in vitro* anticancer activity against breast cancer cell lines (MCF7). Therefore, in this study, 20 hydrazone derivatives were investigated using structure–activity relationships (SAR) and molecular docking to select those with better anticancer activity and higher selectivity. Furthermore, the cytotoxicity of the hydrazone derivatives was assessed against the human hepatocellular carcinoma cell line (HepG2) and liver mesenchymal stem cells using MTT assay and flow cytometry. Their effects on IL-6 and cytochrome *C* levels and tumor cell migration were investigated.

## 2. Materials and methods

### 2.1 Synthesis and characterization of hydrazone derivatives

All chemicals, which are of the highest purity grade, were acquired from Sigma-Aldrich Chemical Co. (St. Louis, MO, USA). The synthesis of hydrazone derivatives was performed as previously reported.^33^ The melting points were measured using a Gallenkamp electric melting point apparatus (Gemini Lab, Apeldoorn, The Netherlands), and the results were uncorrected. Using KBr discs and a FT-IR spectrophotometer, the IR spectra of different compounds were obtained and analyzed. ^1^H-NMR and ^13^C-NMR spectra were assessed in DMSO-d_6_ on a JNM-ECA500II at 500 MHz NMR spectrometer (JEOL Ltd., Peabody, MA) reported as *δ* ppm and using tetramethyl silane (TMS) as internal reference. With the Kratos MS apparatus (Kratos Analytical Ltd, Manchester, UK), mass spectra (EI) were recorded at 70 eV.

### 2.2 Reagents and kits

Dulbecco's modified Eagles culture medium (DMEM), fetal bovine serum (FBS), trypsin/EDTA 0.25%, MTT assay kit, trypan blue 0.4%, and penicillin–streptomycin were purchased from (Lonza, Belgium), while Kreps ringer bicarbonate buffer was purchased from (sigma Aldrich, USA). Bovine serum albumin (BSA) and 1X phosphate buffer saline (PBS) were acquired from (Hyclone, USA). Sorafenib was purchased from (Bayer, Germany).

The high-capacity cDNA reverse transcription kit, TRIzol reagent, and RT-PCR grade water were purchased from (Thermo Fisher Scientific), and the Sso-Fast EvaGreen supermix was from (BIO-RAD). Propidium iodide was purchased from (Miltenyi Biotec), the cytochrome *C* ELISA kit was supplied by (Abcam, Cambridge, UK), and other chemicals and reagents used were of the highest purity grade.

### 2.3 Cytotoxicity

The HepG2 cell line was obtained from ATCC (Rockville, MD). Liver mesenchymal stem cells were harvested from liver biopsy according to previously published protocols.^34^ Cells were grown in DMEM media with 10% fetal bovine serum (FBS), 100 U per mL penicillin, and 100 g per mL streptomycin at 37 °C, 5% CO_2_. The MTT assay kit, which relies on succinate dehydrogenase in living cells' mitochondria to convert the 3-(4,5-dimethylthiazol-2-yl)-2,5-diphenyltetrazolium bromide (MTT) dye to violet formazan crystals, was used to calculate the vitality of individual cells. 6000 cells per well were seeded in the 96-well plate containing, which were then incubated for 24 h. Different concentrations of drug were dissolved in DMEM and then added to the wells to replace the culture medium. The medium was discarded after incubation for 24 h under the same conditions, and 100 μL of MTT (2 mg mL^−1^) were added. After 3 h of incubation at 37 °C, the generated formazan crystals generated were dissolved in 50 μL of DMSO. The optical density was then measured using a Stat Fax ELISA plate reader (Romer Labs, Getzersdorf, Austria) at 570 nm with a reference wavelength of 630 nm after incubating the plate at 37 °C for 15 min. As a positive control, the tyrosine kinase inhibitor sorafenib (Bayer, Germany) was employed. DMSO was used as a solvent and its final concentration was less than 0.2%. SI and IC_50_ were calculated. Triplicates of each *in vitro* test were performed.^33^

### 2.4 Flow cytometry analysis

Cells were harvested, aliquoted up to 1 × 10^6^ cells/100 μL in FACS tubes, washed twice with 2 mL of PBS, centrifuged at 300 × *g* for five minutes, and the buffer was decanted. Reconstituted cells in 100 μL of staining buffer for flow cytometry. HepG2 cells were stained with propidium iodide according to the technique offered *via* the kit and subsequently performed on the cytometer. The distribution of the cell cycles was estimated and analyzed using a FACScan TM device (BD, Franklin Lakes, NJ). Furthermore, the caspase-3 apoptotic marker levels were investigated by flow cytometry. During the flow cytometry assay, we investigated a population of HepG2 cells to obtain enough cells for statistically significant detection.

FITC active caspase-3 apoptosis kit (BD Biosciences, Franklin Lakes, NJ, USA) was used for detection of active caspase 3 followed by flow cytometry analysis according to the instructions of the kit manufacturer.

### 2.5 IL-6 gene expression

After two rounds of PBS washing, total RNA was extracted using the TRIzol Reagent, and cDNA synthesis was performed using the high-capacity cDNA reverse transcription kit in accordance with the manufacturer's instructions. The qPCR reaction mixture contained so-fast EvaGreen supermix (10 μL), cDNA (2 μL), invitrogen RT-PCR grade water (6 μL), and the primer pair (500 nM; forward primer 5′-CAAATTCGGTACATCCTC-3′, reverse primer 5′-CTGGCTTGTTCCTCACTA-3′). The amplification started with heating 10 min at 95 °C, followed by 40 cycles of 15 s at 95 °C, 20 s at 55 °C and 30 s at 72 °C. The amplification data was then analyzed as previously described previously with normalization to β-actin.^35^

### 2.6 Cell migration assay

The wound healing assay was used to investigate the anti-cell migration/antiproliferation effects of synthesized hydrazone derivatives on the HepG2 cell line. In DMEM medium supplemented with 10% fetal bovine serum (FBS), 100 U per mL penicillin, and 100 g per mL streptomycin, cells were cultured at 37 °C and 5% CO_2_. After reaching 80% density, the monolayer cells were scratched using a sterile tip and then washed with phosphate buffered saline, pH 7.4. Different hydrazone derivatives were applied to cells, which were then incubated for 24 h at 37 °C and 5% CO_2_. Finally, an Olympus microscope (Olympus Europa, Hamburg, Germany) was used to take pictures of the wound areas at 0 and 24 h. The relative cell migration was calculated using the following equation:Relative migration = (width_0h_ − width_24h_)/width_0h_

### 2.7 Cytochrome *C* assay

Cytochrome *C* was quantified using a sandwich ELISA kit (Abcam, Cambridge, UK) according to the manufacturer's procedures. After the addition of the TMB substrate and the development of the color, the optical density was measured using a Stat fax microplate reader, USA, at 450 nm.

### 2.8 Docking studies

Molecular Operating Environment (MOE) 2015.10 software was used to perform the docking studies. The 3D structures and conformations of the fibroblast growth factor receptor 4 (FGFR4) complex with *N*-(3,5-dichloro-2-((5-((2,6-dichloro-3,5-dimethoxybenzyl)oxy)pyrimidin-2-yl)amino)phenyl)acrylamide were downloaded from the PDB website (http://www.rcsb.org/; ID: 6NVG). The structures of compound 16 and the standard drug, 5-fluorouracil (5-FU), were drawn using Chem Draw Ultra 16.0 (ChemOffice). Before docking, preparatory steps were performed for the ligand (including protonation, partial charge, and energy minimization in the database) and protein (including elimination of water molecules and repeating chains, addition of hydrogens, calculation of the partial charges, and determination of the active site). The performance of the docking method was assessed by redocking the co-crystalline ligand in the detected active pocket site of FGFR4. The validation of the docking process was affirmed by determining the scoring energy (lower binding energy), the root means standard deviation (RMSD) values, and detecting amino acid interactions for the best pose by free rotation of the rotatable bonds into the rigid receptor binding site. The validation process was carried out by redocking the co-crystal within its binding pocket. A valid performance was verified when an RMSD value less than 2 Å was obtained.

### 2.9 *In silico* ADME simulation

The prediction of the ADME properties of the synthesized compounds was determined from https://www.swissadme.ch/online toolkit according to Lipinski's molecular rules. Lipinski's ‘Rule of Five’ was used to establish the druglikeness of the synthesized hydrazone derivatives compared to the reference drug 5-fluorouracil.

### 2.10 Statistical analysis

Data were expressed as mean ± standard error (SE) of the mean. Statistical significance was determined by one-way ANOVA and Tukey post hoc test using data analyzed using SPSS software version 22 (SPSS Inc., Chicago, IL, United States). A level of *p* < 0.05 was defined as statistically significant.

## 3. Results and discussion

### 3.1 Synthesis of hydrazone derivatives

**Scheme 1:**
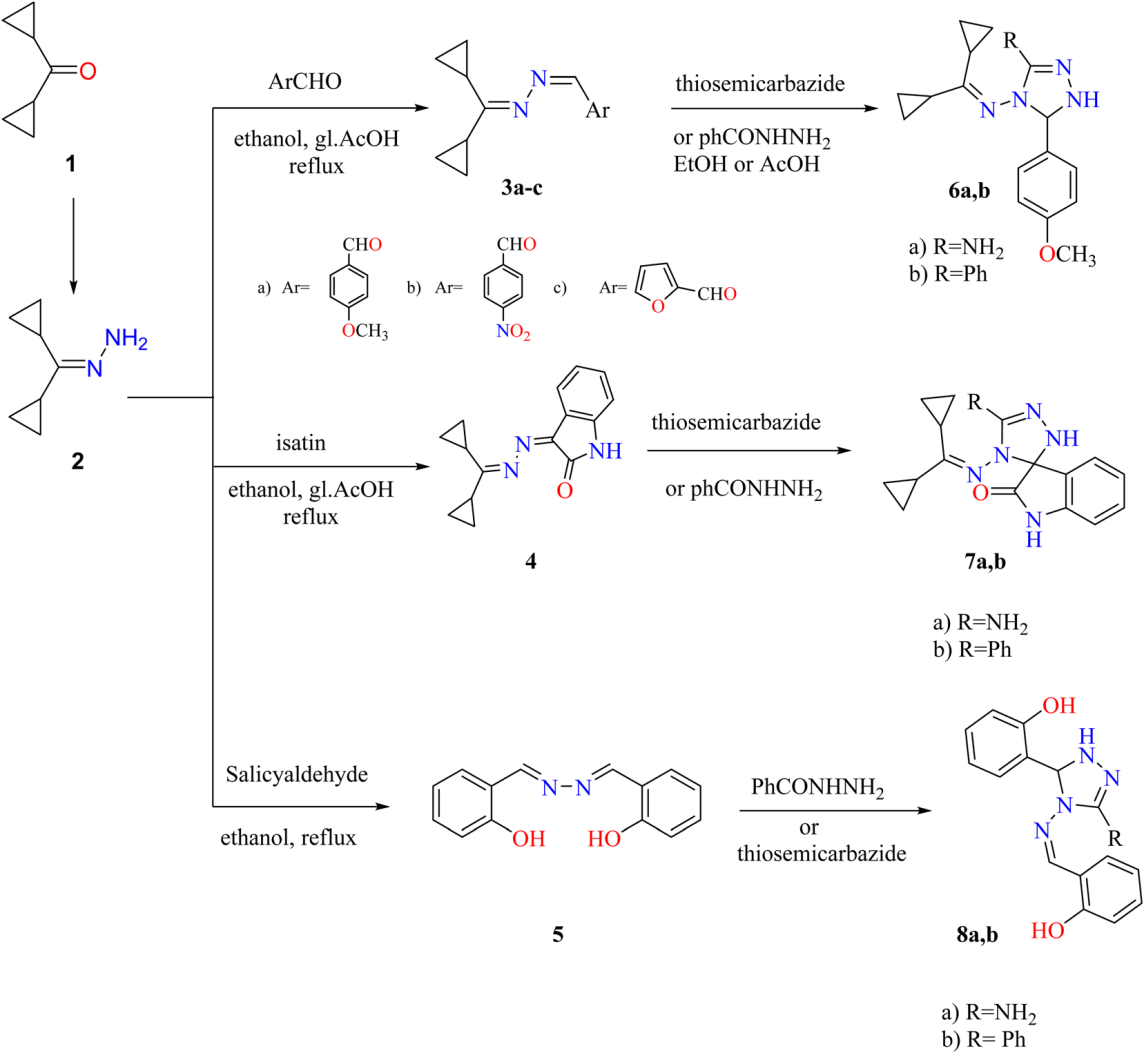
Synthetic route to obtain triazoles 3–8. Reproduced from ref. 33. Copyright (2019), Deuton-X Ltd.

**Scheme 2:**
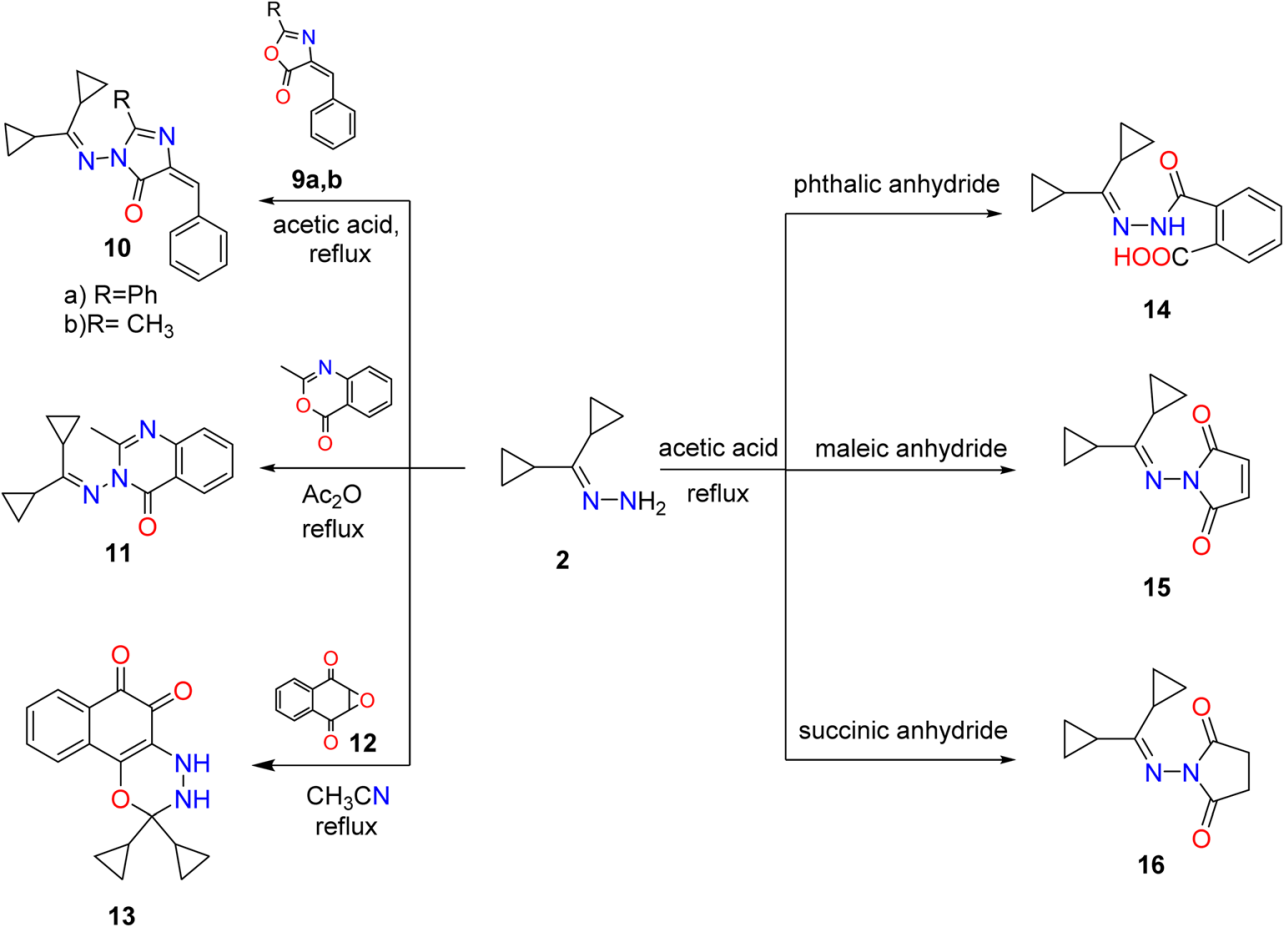
Synthesis of imidazole, cyclic imides, fused diazine, and oxadiazine targets. Reproduced from ref. 33. Copyright (2019), Deuton-X Ltd.

**Scheme 3:**
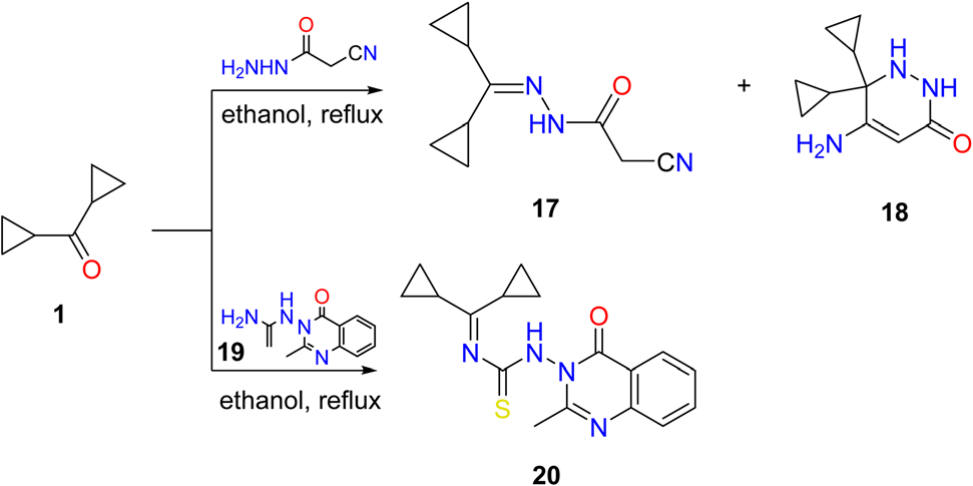
Condensation of dicyclopropyl ketone 1 with hydrazide, and thiosemicarbazide derivatives. Reproduced from ref. 33. Copyright (2019), Deuton-X Ltd.

Dicyclopropyl ketone 1 was condensed with hydrazine hydrate to generate dicyclopropylmethylene hydrazone (2). Compound 2 was used as the lead compound for the synthesis of 3–20 derivatives through the synthetic routes depicted in Schemes 1–3.

Treatment of hydrazone 2 with suitable aromatic aldehydes (such as *p*-anisaldehyde, *p*-nitrobenzaldehyde, furfural, and salicylaldehyde) in refluxed ethanol furnished the corresponding Schiff bases 3a–c and the bisazene derivative 5. Similarly, condensation of hydrazone 2 with isatin (1*H*-indole-2,3-dione) in EtOH and reflux with a few drops of glacial acetic acid (gl. AcOH) gave a high yield of 3-(dicyclopropylmethylene)hydrazone indolin-2-one (4). Triazoles 6a,b, 7a,b, and 8a,b were generated by cycloaddition on the azine derivatives 3a, 4, and 5 with thiosemicarbazide or benzohydrazide (Scheme 1).

Also, synthesis of imidazolones 10a,b by treating hydrazone 2 with oxazolones 9a,b in acetic acid under reflux. Furthermore, the reaction of hydrazone 2 with several anhydrides such as phthalic, succinic, maleic anhydrides proceeded by the same methodology and generated amido and pyrrole derivatives 14–16, respectively. Although 3-((dicyclopropylmethylene) amino)-2-methylquinazolin-4(3*H*)-one (11) was achieved by reaction of hydrazone 2 with an equimolar amount of 2-methylbenzoxazinone in boiling acetic anhydride. Heating of hydrazone (2) (dicyclopropylmethylene) with 2,3-epoxy-1,4-naphthoquinone 12 in CH_3_CN produced naphthoxadiazine-5,6-dione 13 (Scheme 2).

Additionally, a mixture of cyano acetohydrazone and pyridazinone derivatives 17 and 18 was acquired by condensation of 1 with cyano acetic acid hydrazide in boiling ethanol and the two products were easily separated. Finally, dicyclopropyl ketone was treated with the thiosemicarbazide derivative 19 in refluxing ethyl alcohol, providing the corresponding quinazoline derivative 20 (Scheme 3).

The structures of these targets were identified and confirmed based on IR, NMR, and MS spectral and analytical data as previously reported.^33^ FTIR, ^1^H-NMR, ^13^C-NMR, and MS spectra of most derivatives are provided in ESI (Fig. S1 and Table S1†).

### 3.2. Cytotoxicity assay

The cytotoxic effects of the synthetic hydrazone derivatives were compared to those of sorafenib using the HepG2 cell line using the MTT assay. The mean IC_50_ values ranged from 23.6 to 94.7 μM and the SI values are presented in Table 1. The results demonstrated that sorafenib and compounds 2, 4, 8a, 13, 16, 18, and 20 had excellent cytotoxic action against HepG2. Compound 16 had IC_50_ and SI values similar to those of sorafenib, indicating a strong potency and selectivity for activated HepG2 cell lines with little cytotoxicity and the potential to be a successful anticancer agent.

**Table 1:** IC_50_ and SI calculations for sorafenib and the prepared hydrazone derivatives^a^^,^^b^

MTT assay IC_50_ 24 h (μM)			SI
Compounds	HepG2	Liver mesenchymal stem cells	
Sorafenib	25.6	100	3.9
2	94.7	>100	1.58
3a	48.2	>100	—
3b	>100	90.2	—
4	53.7	100	1.86
5	75.8	>100	—
7a	>100	>100	—
7b	>100	100	—
8a	62.2	100	1.6
8b	>100	75.5	—
11	>100	>100	—
13	61.4	85.2	1.38
16	23.6	76.5	3.2
18	89.5	100	1.11
19	>100	>100	—
20	48.6	61.5	1.26

a IC_50_: drug concentration that inhibits cell growth by 50%.

b SI: calculated by dividing the IC_50_ value against liver mesenchymal stem cells for each compound by the IC_50_ value of that compound against the cancer cell line HepG2.

### 3.3 Cell migration assay

**Fig. 2:**
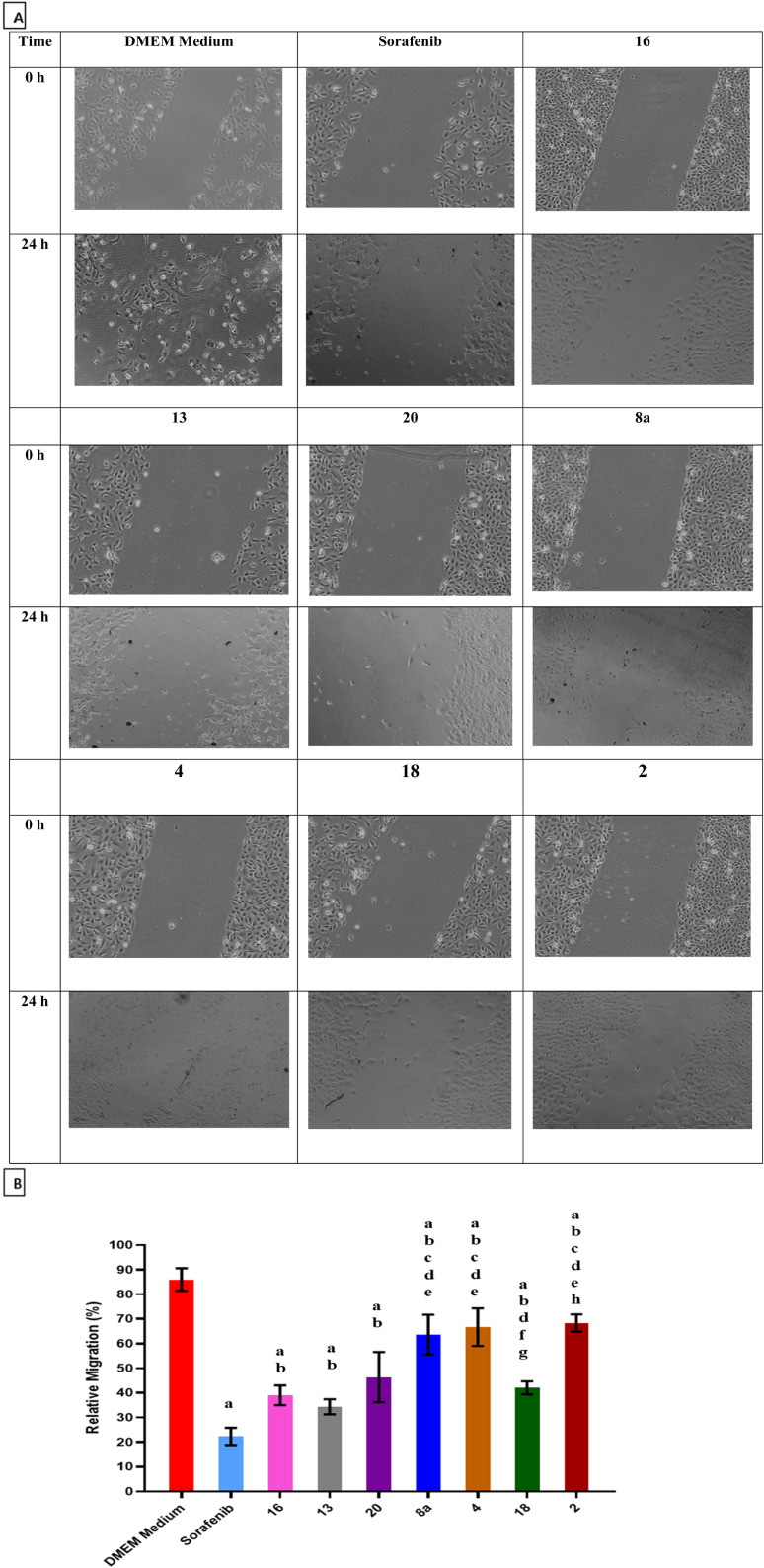
*In vitro* wound healing assay of HepG2 cells treated with sorafenib and synthesized hydrazone derivatives (A) cell morphology before and after drug screening, (B) quantified relative migration summarized as a bar graph. Data are mean ±SD (*n* = 3). (a) significant with DMEM Medium, (b) significant with sorafenib, (c) significant with 16, (d) significant with 13, (e) significant with 20, (f) significant with 8a, (g) significant with 4, (h) significant with 18. Cell migration was dramatically suppressed after treatment with compounds 2, 4, 8a, 13, 16, 18, and 20 for 24 h. Wound healing assay of HepG2 cells treated with treatment with sorafenib and hydrazone derivatives. Compared to the commercially available anticancer drug, sorafenib, the data showed that compounds 16, 13, and 20 had a significant antimigration effect.

The ability of sorafenib and the synthesized hydrazone derivatives to attenuate the migration of HepG2 cells was evaluated using the wound healing assay. Cell migration increased after 24 h for untreated cells (control) but was substantially reduced in the presence of sorafenib and hydrazone derivative compounds 2, 4, 8a, 13, 16, 18, and 20 (Fig. 2). These results suggest that compounds 2, 4, 8a, 13, 16, 18, and 20 delay wound closure and significantly inhibit migration/invasion of HepG2 cells similar to or better than sorafenib. Quantified relative migration calculations showed that compounds 16, 13, and 20 had a significant antimigration and antiproliferative effects on HepG2 cells compared to the commercially available anticancer drug sorafenib.

### 3.4 Apoptosis and cell cycle analyses

**Fig. 3:**
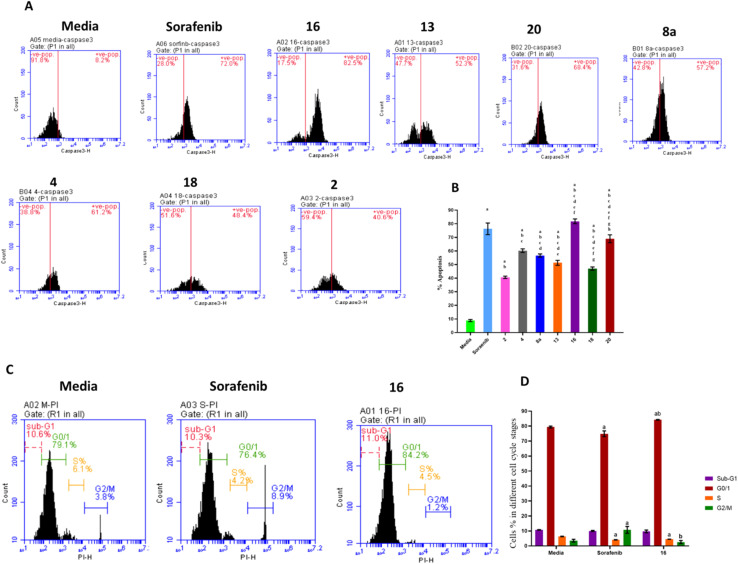
Effects of the selected derivatives of hydrazones on HepG2 cell apoptosis, necrosis, and cell cycle. The treated cells were stained using propidium iodide cell cycle staining and caspase 3 and analyzed by flow cytometry to determine percentages of necrotic and apoptotic cells characterizing cells in different cell cycle stages. (A) Caspase 3 flow cytometry histograms for all derivatives of hydrazone compared to Sorafinib. (B) The quantified percentages are summarized as a bar graph. Data are mean ±SD (*n* = 3). (a) significant with DMEM Medium, (b) significant with Sorafenib, (c) significant with 2, (d) significant with 4, (e) significant with 8a, (f) significant with 13, (g) significant with 16, (h) significant with 18. Treatment with sorafenib and hydrazone derivatives (compounds 2, 4, 8a, 13, 16, 18, and 20) significantly increased the apoptotic percentage of HepG2 cells compared to the control group. Compound 16 caused a higher percentage of apoptosis compared to sorafenib and the other derivatives. (C) Histograms showing different cell cycle stages for compound 16 compared to sorafenib and media. (D) The quantified percentages are summarized as a bar graph.

The effects of treatment with sorafenib and hydrazone derivatives on HepG2 cells were investigated. Apoptotic and necrotic effects of the tested compounds on HepG2 cells were evaluated using caspase 3 assay kit which employs an FITC-antibody reported to specifically recognize the active form of caspase 3 (not its proenzyme). On the other hand, cell cycle arrests at different stages, caused by treatment with hydrazone derivatives were assessed using propidium iodide staining (Fig. 3, ESI Fig. S2 and S3†).

Caspase 3 is synthesized as a proenzyme that is cleaved by other proteases into small and large subunits which then associate to form the active enzyme. Active caspase 3 proteolytically cleaves other caspases and cellular targets leading to execution of apoptosis. Caspase 3 flow cytometry analysis of HepG2 cells treated with the synthesized hydrazone derivatives or sorafenib was conducted and the results showed that, compared to the control group, the percentage of apoptotic cells increased significantly after treatment with sorafenib and the derivatives of hydrazone (2, 4, 8a, 13, 16, 18, and 20). Treatment with compound 16 showed a higher increase in the percentage of apoptotic cells (80%) than sorafenib (75%) (Fig. 3A and B). This explains the key effect, of the synthesized hydrazone derivatives on caspase-3 activation that leads to cellular apoptosis upregulation that supports the anticancer potential of the synthesized compounds especially compound 16.

Additionally, the anticancer potential of the most potent hydrazone derivatives and sorafenib on the cell cycle progression, crucial for proliferation of cancer cells, was determined. Flow cytometry cell cycle analysis (Fig. 3C and D) was used to investigate the effect of compound 16 on HepG2 cell cycle stained with propidium iodide. Approximately 79.38% of untreated HepG2 cells were in the G0/G1 phase, 6.23% in the S-phase, and 3.5% in the G2/M phase. For cells treated with sorafenib, a positive control, 74.93% of cells were in the G0/G1 phase, 4.17% in S-phase, and 10.68% in G2/M. The most potent anticancer hydrazone derivative, compound 16, reduced the number of cells in S-phase to 4.5% while 2.5% of the cell population was in the G2/M phase which suggest a significant inhibitory effect of compound 16 on the HepG2 cells. These data confirm that compound 16 has a greater impact on HepG2 cell cycle when compared to sorafenib. This cellular proliferation inhibitory effect of the synthesized hydrazones, especially compound 16, could enable their application as anticancer agents following further assessments. Additional flow cytometry graphs illustrating the effects of different treatments on caspase 3 levels and cell cycle of HepG2 cells can be found in ESI.†

### 3.5 Effects of sorafenib and compound 16 treatment on IL-6 gene expression and cytochrome *C* concentration

**Fig. 4:**
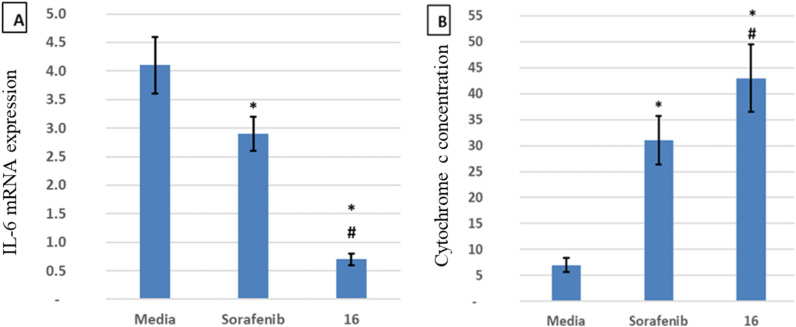
Sorafenib and compound 16 effects on (A) IL-6 mRNA expression (RT-PCR) and (B) cytochrome *C* concentration (ELISA). All samples were measured separately in duplicate, and the data were provided as mean standard deviation. *Statistical significance compared to the control group (*p* < 0.001); # statistical significance compared to the sorafenib group (*p* < 0.001). Treatment with compound 16 showed a significant decrease in the level of IL-6 and an increase in cytochrome *C* greater than sorafenib. Therefore, the proposed anticancer mechanism of compound 16 may include a marked reduction in the expression of the IL-6 gene.

**Fig. 5:**
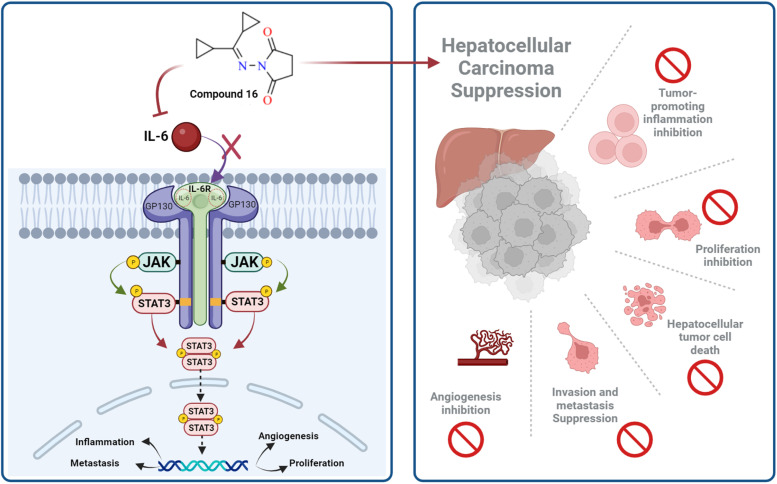
Proposed mechanism by which compound 16 may exert its anticancer effect.

IL-6 plays a key role in the regulation, inflammation, and oncogenesis of the immune system.^36^ When IL-6 binds to its receptor, glycoprotein 130, JAK phosphorylates the receptor, activating the JAK/STAT3 signalling pathway. Through the reduction of oxidative damage and inhibition of the apoptotic cascade, these pathways play a crucial role in liver regeneration.^37^ However, continuing activation of the IL-6 signalling pathway is detrimental to the liver and can eventually lead to HCC progression or recurrence.^12^ High serum levels of IL-6 have been suggested as a tumor marker of HCC. Furthermore, IL-6 has been reported to protect cancer cells against DNA damage, oxidative stress, and apoptosis caused by cancer therapy.^38^ Inhibition of IL-6 was proposed as a possible therapeutic strategy to make HCC cells more susceptible to sorafenib.^39^ Because the JAK/STAT3 cascade is critical for the development of HCC, and various intracellular signalling pathways are activated by the IL-6 cytokine, a potential therapeutic approach for the treatment of malignancies could involve blocking IL-6 or interfering with its signalling pathways alone or in conjunction with traditional anticancer medicines. Cellular death is significantly influenced by cytochrome *C*.^40^ The expression of the mitochondrial respiratory chain proteins cytochrome *C*, cytochrome *C* oxidase subunits I and IV, and the manganese superoxide dismutase, which scavenges free radicals, increases in response to chemotherapy. An apoptotic signal is produced when Bax encourages the release of cytochrome *C* from mitochondria, which in turn triggers the caspase 9 apoptotic pathway. Caspase 9 activates downstream caspases, including caspase 3, which execute apoptosis and cell death.^12,41^ Cytochrome *C* is released from injured mitochondria into plasma after induction of apoptosis and its serum levels could be used to assess the effect of chemotherapy. In this study, the administration of sorafenib and compound 16 caused a significant (*P* < 0.001) decrease (*P* < 0.001) in the expression of IL-6 (Fig. 4A) and a significant increase in cytochrome *C* concentration (Fig. 4B) compared to the control group. Treatment with compound 16 showed a significant decrease in the level of IL-6 level and an increase in cytochrome *C* greater than sorafenib. Therefore, the proposed anticancer mechanism of compound 16 may include a marked reduction in IL-6 gene expression that causes suppression of the JAK/STAT3 pathway and ultimately decreases inflammation and tumorigenesis, as shown in Fig. 5.

### 3.6. Molecular docking studies

**Fig. 6:**
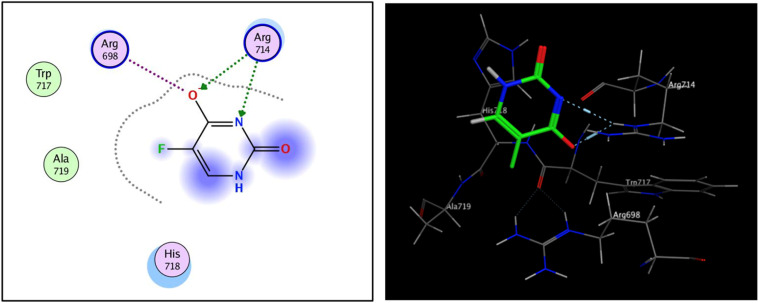
2D and 3D interaction analysis of structural redocked with 5-fluorouracil with FGFR4 showing clear active site interactions.

**Fig. 7:**
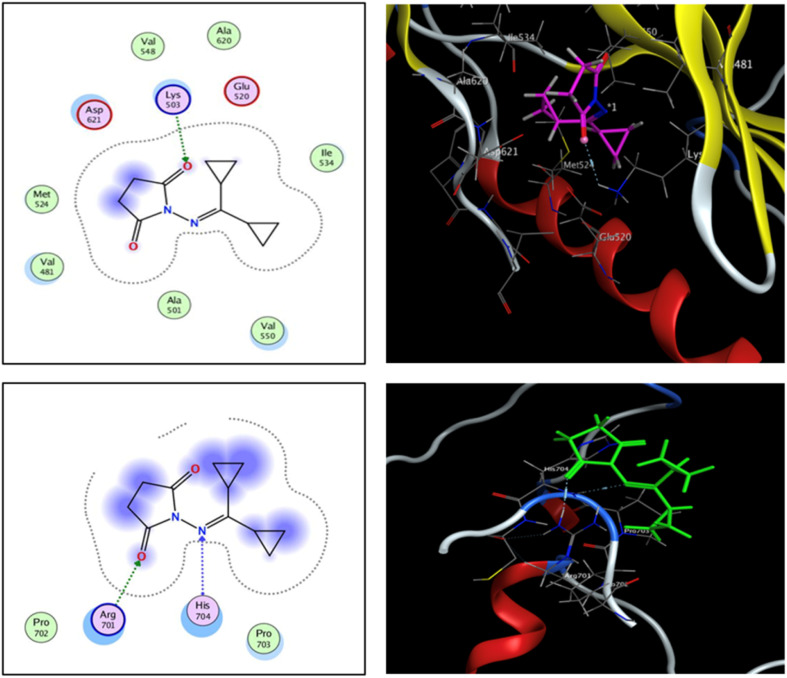
2D and 3D interaction analysis of 16 newly redocked compounds with two different pockets of FGFR4 showing significant active site interactions.

The fibroblast growth factor receptor (FGFR) family of receptor tyrosine kinases consists of 4 members (FGRF 1–4) encoded by different genes (50–70% sequence homology). FGFRs are key to activation of mitogen-activated protein kinase; phosphatidylinositol 3-kinase, phospholipase Cγ, and signal transducer and activator of transcription (STAT) which lead to activation of target genes responsible for cellular proliferation, metastasis, angiogenesis, and development of HCC.^42^ The fibroblast growth factor receptor 4 (FGFR4) is involved in cell proliferation, differentiation, and migration, and its abnormal signalling was linked to development and progression of HCC. Similar to other tyrosine kinases, FGFR consists of an extracellular receptor domain and a transmembrane helix connected to a cytoplasmic kinase domain.^36,43–45^ Therefore, compound 16 and a standard drug, 5-fluorouracil, were docked to the active site of the FGFR4 kinase domain (PDB ID: 6NVG) to predict their ability to inhibit the kinase function using Molecular Operating Environment 2015.10 (MOE) software to analyze all docking poses and binding energies (Fig. 6 and 7). RMSD was employed to measure the overall stability of the protein and ligand in relation to the initial protein backbone structure. Therefore, validation of the docking protocol indicates that the ligand is confirmed by the active site pocket with a score = 4.6292 kcal mol^−1^ and *r*_smd_ = 1.1392 Å. Compound 16 presented better binding interactions compared to 5-fluorouracil (score = −3.8501 kcal mol^−1^, *r*_smd_ = 1.2706 Å). The imino and carbonyl groups of compound 16 showed hydrogen bond interactions with His 704 and Arg 701 amino acids. On the other hand, when the same pose was docked with a different pocket on the same protein, compound 16 also formed a hydrogen bond with Lys 503. These interactions suggest a strong polar connection between compound 16 and the binding pocket of the protein, potentially stabilizing the complex (Table 2 and Fig. 7). In addition to hydrogen bonds, hydrophobic interactions (typically enable the molecule to fit more tightly into the binding site and increase its affinity for the protein) are achieved by the orientation of compound 16 within the protein pocket which contribute to the binding stability of compound 16. The ability of compound 16 to display better binding interactions to EGFR4 as compared to 5-FU, a common chemotherapeutic drug used for treatment of several cancers, supports its potential as an effective anticancer agent.

**Table 2:** Ligand interactions report for compound 16 and 5-fluoruracil

Ligand		Receptor	Interaction Distance (^o^A)	*E* (kcal mol^−1^)
Compound 16	N 5	N HIS 704	H-acceptor 3.49	−1.0
	O 15	NH1 ARG 701	H-acceptor 2.96	−4.8
	O 5	N Lys 503	H-acceptor 3.28	−0.7

5-Fluorouracil	O 5	NE ARG 714	H-acceptor 2.99	−5.5
	N 6	NE ARG 714	H-acceptor 3.43	−1.7
	O 5	NE ARG 698	Ionic 3.80	−0.9

### 3.7 *In silico* ADME predictions

The preferable oral bioavailability of the compounds examined was confirmed by *in silico* ADME predictions (log *P* < 5, *M*_w_ < 500, HBA 10 and HBD <5). The number of values of rotatable bonds (*n*-ROTB) (which should be <10), indicated molecular flexibility. The topological polar surface area (TPSA) revealed that most of the calculated values of the target compounds indicated good permeability. According to ADME results (Table 3), the compounds tested did not violate Lipinski's rules, which provides positive values of drug similarity. Skin permeation (log *K*_p_; cm s^−1^) showed that the more negative the log *K*_p_, the less skin permeant for each compound.^46^ The Lipinski qualitative model (mostly known as rule of five) is a widely used approach for prediction of the likelihood of a small compound being orally bioavailable. It is used in early drug development to guide compound selection by using criteria such as molecular weight, lipophilicity (log *P*), hydrogen bond donors, and acceptors. It helps to streamline the prioritize selection of compounds more likely to be absorbed in the gastrointestinal tract and consequently and potentially succeed in clinical trials. Several studies have proven a strong correlation between its criteria and the pharmacokinetic properties of successful drugs. Further, it assists in evaluating ADME parameters for drug candidates and molecules and offers insights that help address uncertainties early in the drug discovery process. Therefore, the predicted oral bioavailability was summarized using the bioavailability radar, in which the pink zone represents the ideal ranges of different characteristics (lipophilicity: XLOGP3 0.7 to +5.0, size 150–500 g mol^−1^, polarity: TPSA between 20 and 130, solubility: logarithmic *S* with maximum value 6, saturation: the fraction of hybridized carbons sp^3^ with minimum value 0.25, and flexibility: rotatable bonds with maximum value 9). Accordingly, all of the evaluated hydrazones exhibit the probability of being orally active drug-like candidates. Compared to the reference drug (5-fluorouracil), the hydrazone derivatives tested showed greater bioavailability (Table 4). These *In silico* ADME predictions are matched with the *in vitro* investigations that showed that compared to the control group, the percentage of apoptotic cells increased significantly after treatment with sorafenib and the derivatives of hydrazone (2, 4, 8a, 13, 16, 18, and 20) and the treatment with compound 16 showed a higher increase in the percentage of cell apoptosis than sorafenib (Fig. 3A and B). Also, these computational studies matched with cell cycle analysis as for cells treated with sorafenib, 74.93% of cells were in the G0/G1 phase, 4.17% in S-phase, and 10.68% in G2/M while the most potent anticancer hydrazone derivative, compound 16, reduced the number of cells in S-phase to 4.5% while 1.2% of the cell population was in the G2/M phase which suggest a significant inhibitory effect of compound 16 on the HepG2 cells (Fig. 3C and D). Moreover, treatment with compound 16 showed a significant decrease in the level of IL-6 level and an increase in cytochrome *C* greater than sorafenib. Therefore, the proposed anticancer mechanism of compound 16 may include a marked reduction in IL-6 gene expression that causes suppression of the JAK/STAT3 pathway and ultimately decreases inflammation and tumorigenesis, as shown in Fig. 5.

**Table 3:** *In silico* drug-likeness ADME predictions according to the Lipinski rule^a^

Compounds	*M*_w_ (g mol^−1^)	log *P*_0_/*w* (MLO GP)	TPSA (Å)	*n*-ROTB	HBD	HBA	Drug likeness/violation	log *K*_p_ (skin permeation) (cm s^−1^)
4	253.31	2.03	53.82	3	1	3	Yes/0	−6.35
8a	297.32	2.03	53.82	3	1	3	Yes/0	−6.35
13	296.33	1.43	67.43	2	2	3	Yes/0	−6.42
16	206.25	1.76	49.74	3	0	3	Yes/0	−7.38
18	193.25	1.69	63.81	3	2	1	Yes/0	−6.80
20	326.43	3.66	91.37	5	1	3	Yes/0	−6.74
5-Fluorouracil	130.08	−0.42	66.24	0	2	5	Yes/0	−6.91

a Molecular weight (*M*_w_) < 500, partition coefficient between *n*-octanol and water (log *P*) < 5.0, topological polar surface area (TPSA) < 140 Å:, number of rotatable bonds (*n*-RB) <10, hydrogen-bond donor (HBD) <5, and hydrogen-bond acceptors (HBA) <10.

**Table 4:** Bioavailability radar of different hydrazone derivatives^a^

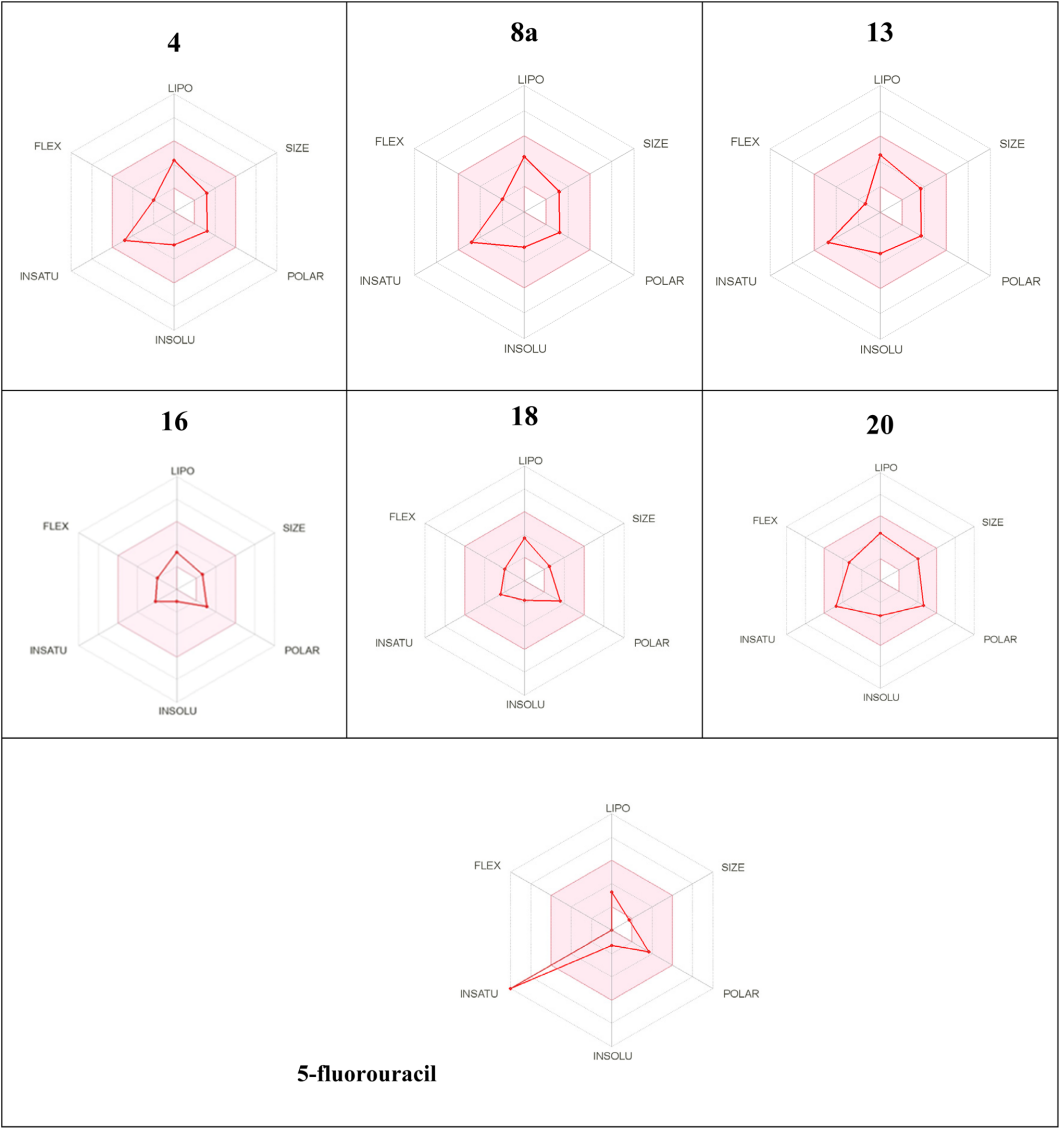

a The pink area represents the optimal range for each properties as follows: lipophilicity (LIPO): XLOGP3 between 0.7 and + 5.0, Size: *M*_w_ from 150 to 500 g mol^−1^; polarity (POLAR): TPSA between 20 and 130 2, insolubility (INSOLU): log *S* not greater than 6; saturation (INSATU): fraction of hybridized carbons of sp^3^ not less than 0.25; and flexibility (FLEX): no more than 9 rotatable bonds.

### 3.8. Structure–activity relationship (SAR)

**Fig. 8:**
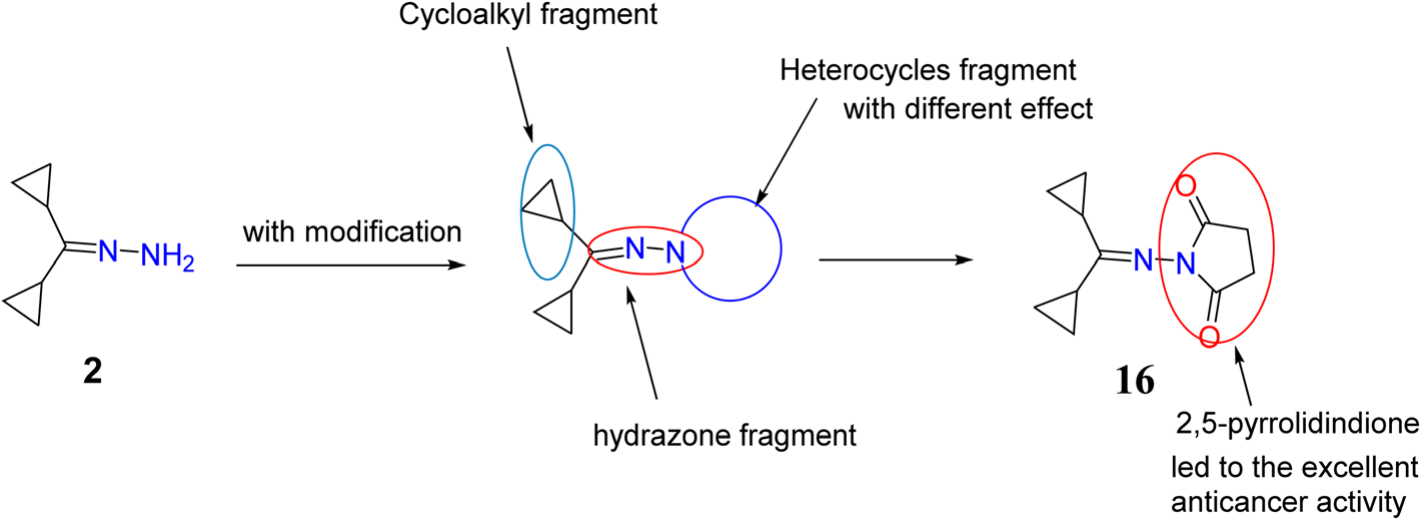
Chemical structure of target compounds with important sites for the structure–activity relationship (SAR).

The synthesized compounds were characterized and evaluated against anticancer activities that support the described SAR (Fig. 8). In a previous investigation of lead cyclopropyl hydrazone 2 with its structural modifications, the presence of the cyclopropyl ring^47,48^ and the hydrazone fragment^49,50^ was predicted to exert broad cytotoxic activities against cancer cells. To further enhance its potential as an anticancer agent, important modifications such as the addition of *N*-heterocycles were introduced to the lead compound.

As evidenced by the effects of the synthesized compounds in the MTT assay, compounds 2, 4, 8a, 13, 16, 18, and 20 possessed the highest cytotoxic activities against HepG2 cells. Although the starting material, compound 2, with the basic structure of cyclopropyl rings, hydrazone, and primary amine exerted moderate cytotoxicity against HepG2 cells, it was found that cyclization of the amino group to a heterocycle moiety or introduction of functional group such as formation of cyanoacetamide fragment (compound 18) increased its potency and selectivity. This may be essential to achieve good binding interactions between the tested compounds and the receptor, possibly through the formation of a hydrogen bond.

Similarly, it is suggested that the incorporation of indoline-2-one (compound 4), aminotriazole moiety and imino group (compound 8a) and oxadiazine-5,6-dione (compound 13) is responsible for their biological significance. Introducing a thiourea fragment substituted with a 4-oxoquinazoline moiety remarkably improved the cytotoxic activity. However, compound 16 with the 2,5-pyrrolidinone moiety^50^ showed an excellent effect compared to sorafenib and the other compounds tested, possibly due to the formation of hydrogen bonds with the receptor (due to the presence of an imide group). Treatment with compound 16 showed a significant increase in cytochrome *C* greater than that observed upon treatment with sorafenib. Chemotherapeutic agents are reported to significantly increase cytochrome *C* release from the mitochondrial matrix into the cytosol in addition to the other mitochondrial respiratory chain proteins cytochrome *C* oxidase subunits I and IV, and manganous superoxide dismutase (which scavenges free radicals). The released cytochrome *C* indirectly activates caspase-9 and caspase-3 which ultimately execute apoptosis.^51–53^ This study showed that treatment of HepG2 cells with compound 16 led to high increase in activation of cytochrome *C* and activated caspase 3 which represent significant markers of apoptosis. This supports the potential role of the synthesized hydrazone derivatives, especially compound 16, as anticancer agents mediated by their ability to trigger cellular apoptosis.

To the best of our knowledge, this report is the first to investigate the potential of the newly synthesized hydrazone derivatives for hepatocellular carcinoma treatment. However, further validations such as assessing the effect of hydrazone derivatives, namely compound 16, on the proteins involved in cell proliferation, apoptosis, JAK/STAT3 pathway, as well as animal studies to confirm the anticancer effects of hydrazone derivatives are required. A further computational molecular dynamics simulation and docking study compared with a co-crystallized ligand are required to better elucidate the interaction between the protein and the ligand.

## 4. Conclusions

Despite recent improvements in the management of HCC, it remains one of the leading causes of cancer-related mortality. Various molecular targets involved in signaling pathways that regulate tumor proliferation and angiogenesis that characterize HCC were identified. The first HCC medication approved by the FDA is the oral multikinase inhibitor sorafenib. This first-line treatment may not work for all patients for various causes, including drug resistance. Therefore, it is necessary to develop additional effective HCC drugs.

In the current study, new hydrazone derivatives with improved activities and selectivity were synthesized and evaluated *in silico* by molecular docking and structure–activity correlations (SAR) in addition to *in vitro* studies using HepG2 human hepatocellular carcinoma cell line and liver mesenchymal stem cells as a normal cell line. With mean IC_50_ values ranging from 23.6 to 94.7 μM, compounds 2, 4, 8a, 13, 16, 18, and 20 exhibited high cytotoxic effects against HepG2 cells. Compound 16 had IC_50_ and SI of 23.6 μM and 3.2, respectively, against HepG2 as compared to sorafenib (25.6 μM. and SI 3.9) indicating a strong potency and selectivity for activated HepG2 cell lines. Compound 16, however, exhibited low cytotoxicity against liver mesenchymal stem cells (IC_50_ 76.5 μM). *In vitro* wound scratch assay indicated that several synthesized hydrazone derivatives, including compound 16, attenuated HepG2 cell migration and wound closure by 40–70%. HepG2 cell apoptosis as determined by assay of active caspase 3 enzyme, showed that the most potent hydrazone derivative, compound 16, caused a higher increase in the percentage of cell apoptosis (80%) as compared to sorafenib (75%). Cell cycle analysis showed that compound 16 reduced the number of cells in S-phase to 4.5% while 1.2% of the cell population was in the G2/M phase which suggest a significant inhibitory effect of compound 16 on the HepG2 cells. Furthermore, compound 16 significantly increased cytochrome *C* release and significantly decreased IL-6 gene expression compared to sorafenib.

These findings suggest that compound 16 might be a potent anticancer candidate for HCC treatment by downregulating the expression of the IL-6 gene that causes suppression of the JAK/STAT pathway, decreases inflammation, and tumorigenesis with favorable binding interaction and bioavailability. Computational analysis including molecular docking and structure–activity correlations also supported the anticancer potential of the synthesized hydrazone derivative 16. Compound 16 displayed better binding interactions to EGFR4 as compared to 5-FU which further supports the potential of compound 16 as an effective chemotherapeutic agent. Furthermore, ADME predictions supported preferable bioavailability of the synthesized hydrazones.

EGFR and IL-6 signaling pathways crosstalk in multiple downstream signaling pathways and both are linked to tumorigenesis. Therefore, co-targeting them could represent a promising strategy for effective cancer treatment.^54^ Findings of this study indicate that compound 16 targets both signalling pathways by binding to the kinase domain of EGFR4 and decreasing IL-6 expression. Further computational, *in vitro*, and *in vivo* evaluations should be considered to support the anticancer potential of the synthesized novel hydrazone derivatives.

## Data availability

The data supporting this study are available in the main manuscript and its ESI.†

## Author contributions

All authors contributed equally to the experimental and writing parts depending on their specialty. All authors read and approved the final version of this manuscript.

## Conflicts of interest

The authors declare no conflict of interest.

## Supplementary Material

RA-014-D4RA05854B-s001
