# Supplementary material for: Anti-hepatocellular carcinoma activities of novel hydrazone derivatives via downregulation of interleukin-6
Source: RSC Adv. 2024 Nov 28;14(51):37960–74. doi: 10.1039/d4ra05854b (PMC11603412; doi:10.1039/d4ra05854b)
Supplement: RA-014-D4RA05854B-s001 [file RA-014-D4RA05854B-s001.pdf]

Supplementary Information (SI) for RSC Advances.
This journal is © The Royal Society of Chemistry 2024

Supporting Information

Anti-Hepatocellular Carcinoma Activities of Novel Hydrazone Derivatives Via Downregulation of Interleukin-6

Ahmed Nabil * [1,2,3,] Marwa Abdel-Motaal[*4,5], Ayman Hassan[3], Mohamed M. Elshemy[6], Medhat asem[7], Mariam Elwan[8], Mitsuhiro Ebara [1,9, 10], Mohammed Abdelmageed [11, 12], Gamal Shiha [3, 13], Hassan M. E. Azzazy[14*]

[1] Research Center for Macromolecules and Biomaterials, National Institute for Materials Science (NIMS), Tsukuba 305-0044, Japan.
[2] Biotechnology and Life Sciences Department, Faculty of Postgraduate Studies for Advanced Sciences (PSAS), Beni-Suef University, Beni-Suef, Egypt.
[3] Egyptian Liver Research Institute and Hospital (ELRIAH), Sherbin, El Mansoura, Egypt.
[4] Chemistry Department, College of Science, Qassim University, Qassim, Buraydah, 51452 Saudi Arabia.
[5] Chemistry Department, Faculty of Science, Mansoura University, Mansoura, 35516 Egypt.
[6] Faculty of Science, Menoufia University, Menoufia, Egypt.
[7] Department of Civil Engineering, College of Engineering and Information Technology, Onaizah Colleges, Qassim, Saudi Arabia.
[8] Egyptian Ministry of Health, El Mansoura, Dakahlia, Egypt.
[9] Graduate School of Pure and Applied Sciences, University of Tsukuba, 1-1-1 Tennodai, Tsukuba, Ibaraki 305-8577, Japan.
[10] Graduate School of Industrial Science and Technology, Tokyo University of Science, 6-3-1 Niijuku, Katsushika-ku, Tokyo 125-8585, Japan.
[11]Department of Pharmacology and Toxicology, Faculty of Pharmacy, Buraydah Colleges, Qassim, Saudi Arabia
[12]Hot Laboratory Center, Atomic Energy Authority, Cairo, Egypt
[13]Hepatology and Gastroenterology Unit, Internal Medicine Department, Faculty of Medicine, Mansoura University, Egypt.
[14]Department of Chemistry, School of Sciences & Engineering, The American University in Cairo, New Cairo, Egypt, 11835

***Correspondence may be addressed to:**
Marwa Abdel-Motaal; ma.mohammed@qu.edu.sa, dr_marwachem@mans.edu.eg, Tel +966569909737

Ahmed Nabil; TOLBA.AhmedNabil@nims.go.jp Tel.: (008180-3540-4321, +201000618349)

Hassan M. E. Azzazy; hazzazy@aucegypt.edu, Tel: 00201000565727

Table of contents:

Figure S1. Spectral analyses of different hydrazone derivatives.

Table S1. Physicochemical and spectroscopy data for the synthesized compounds

Figure S2. Caspase 3 assay.

Figure S3. Cell cycle analysis.

Figure S1. Spectral analyses of different hydrazone derivatives.

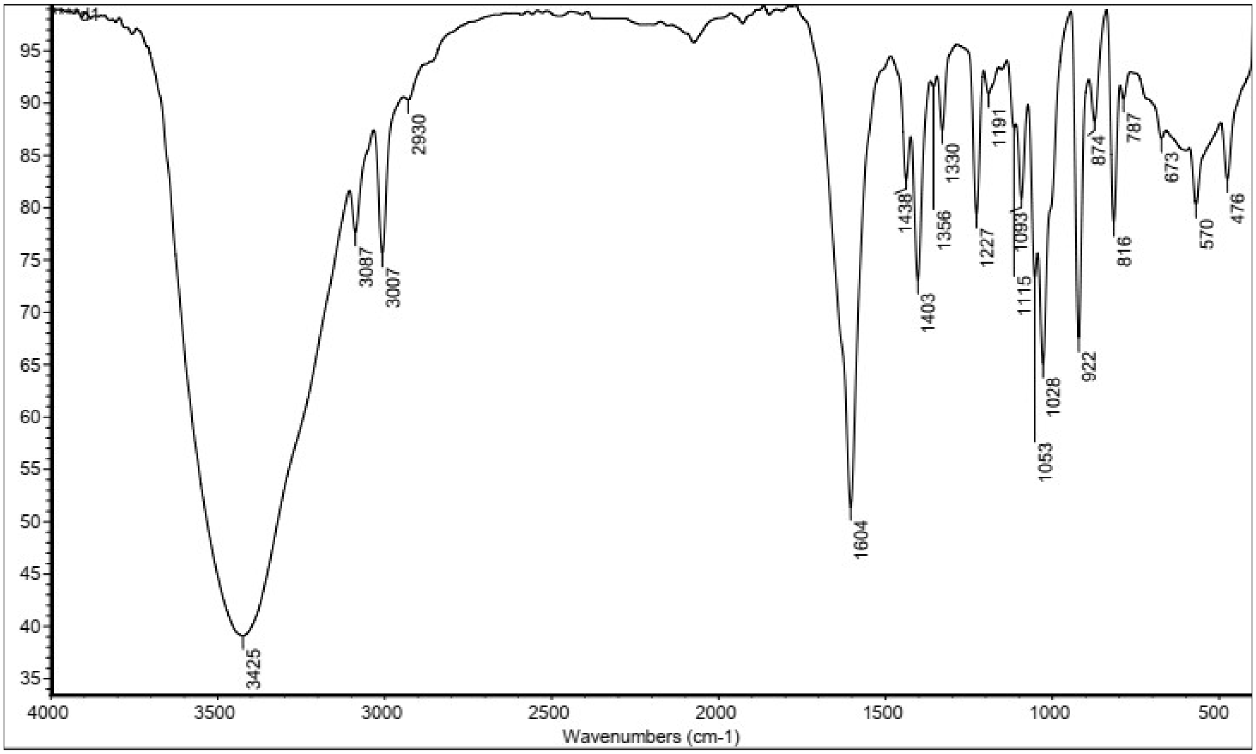

FTIR spectrum of compound 2.

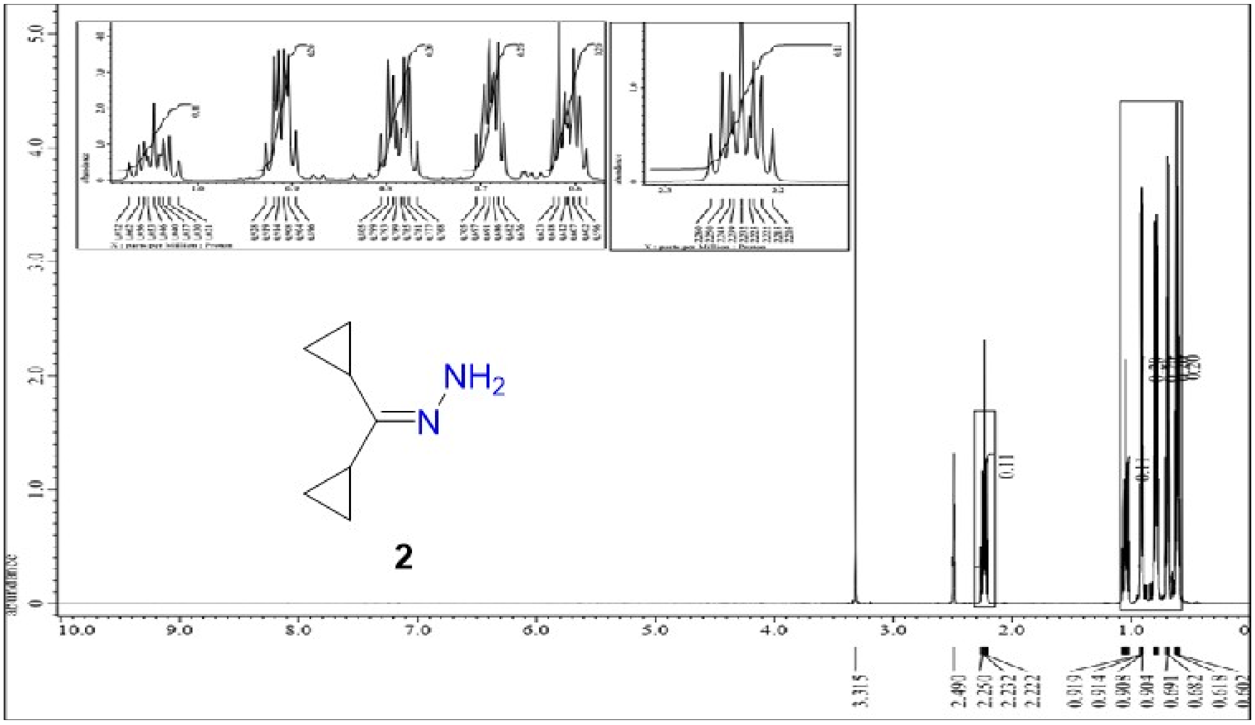

^{1}H-NMR (500 MHz, DMSO) spectrum of compound 2.

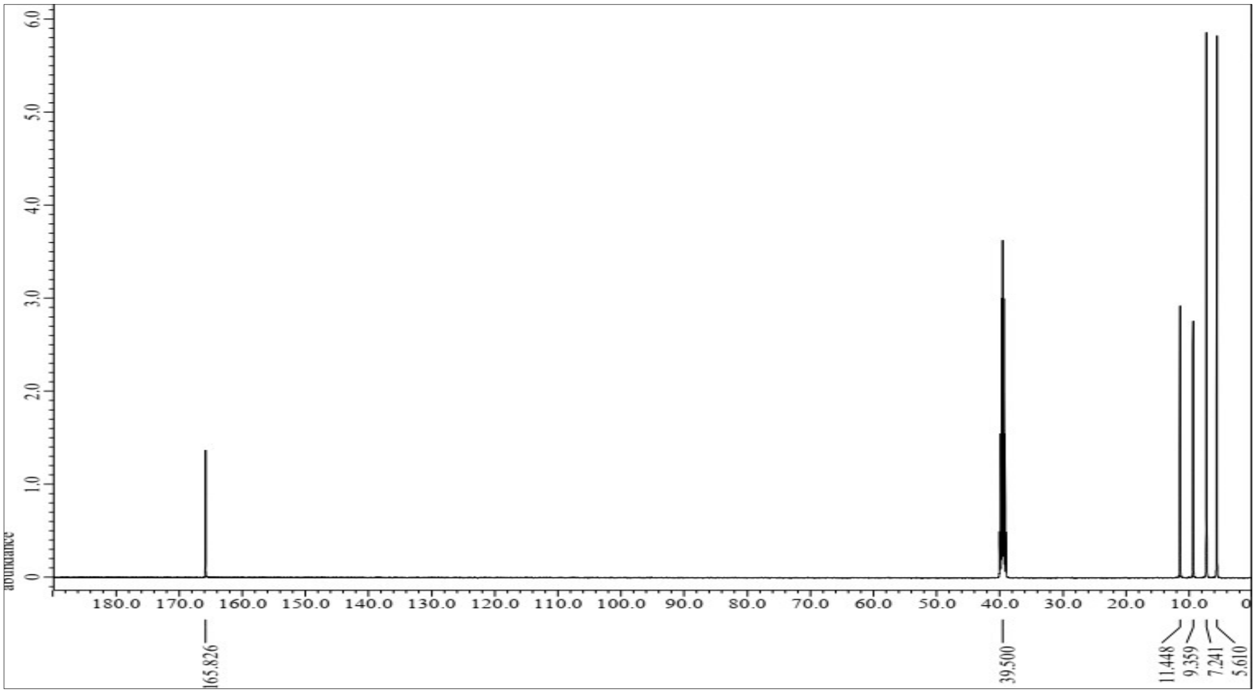

^{13}C-NMR spectrum (125 MHz, DMSO) spectrum of compound 2.

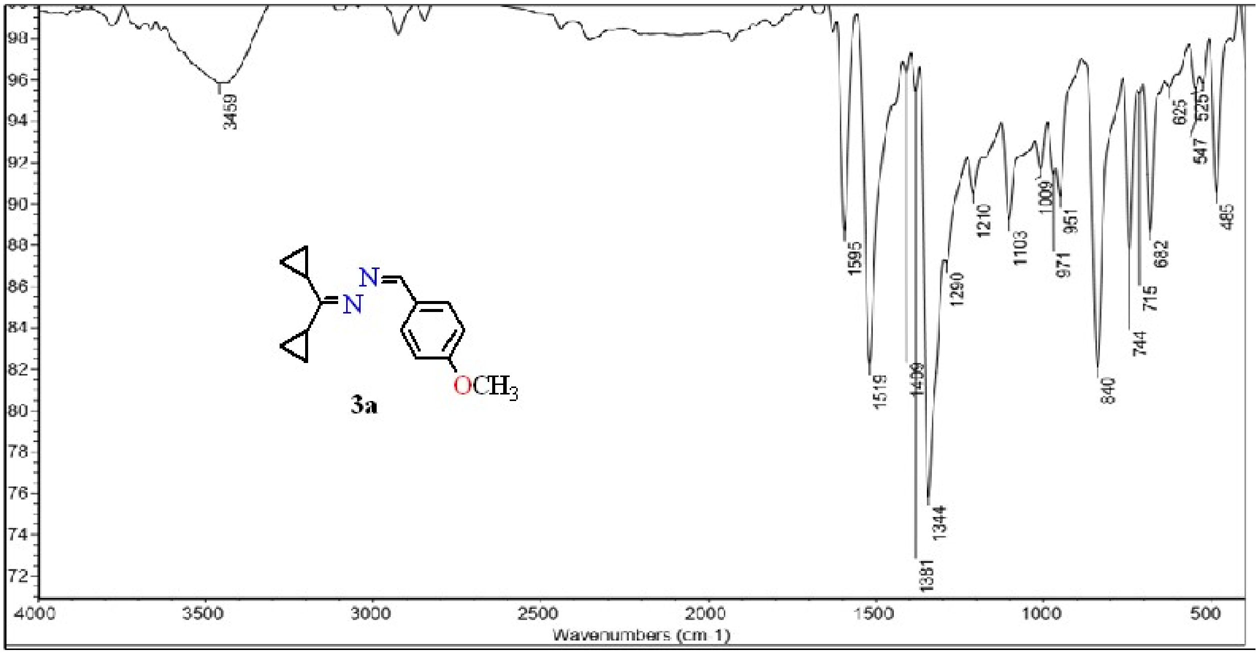

FTIR spectrum of compound 3a.

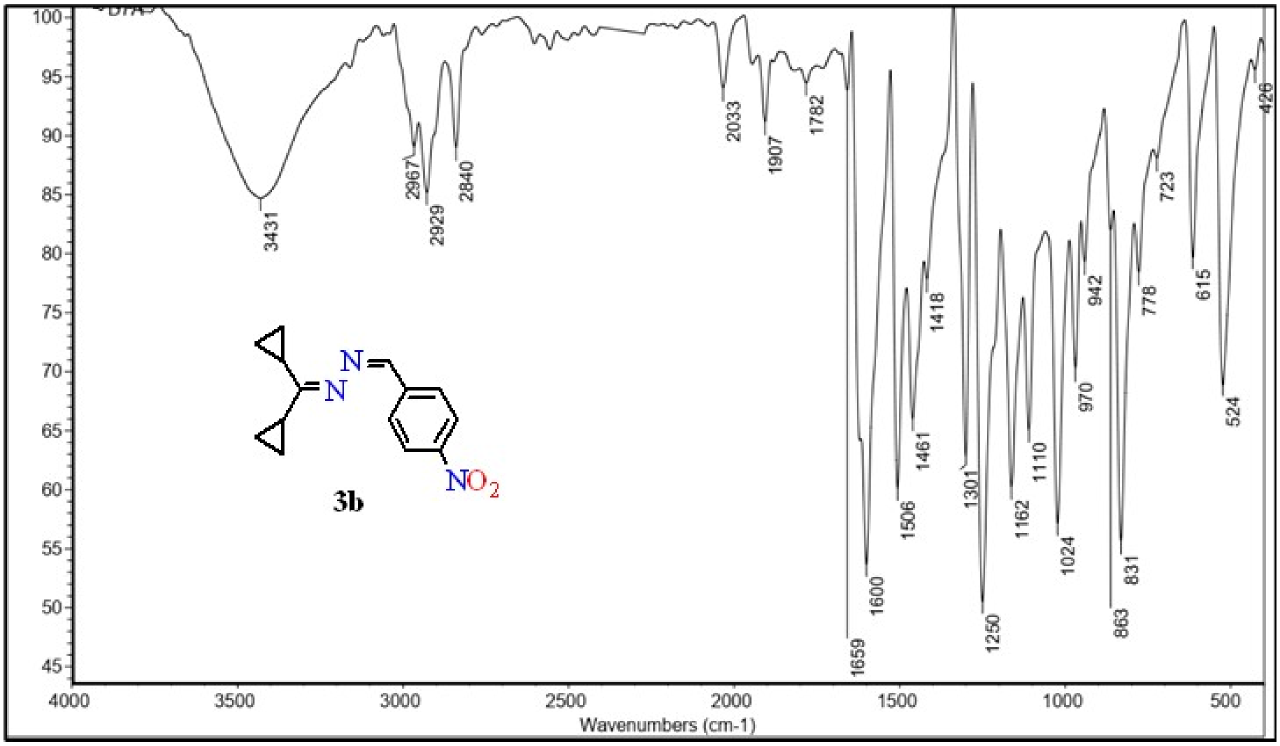

FTIR spectrum of compound 3b.

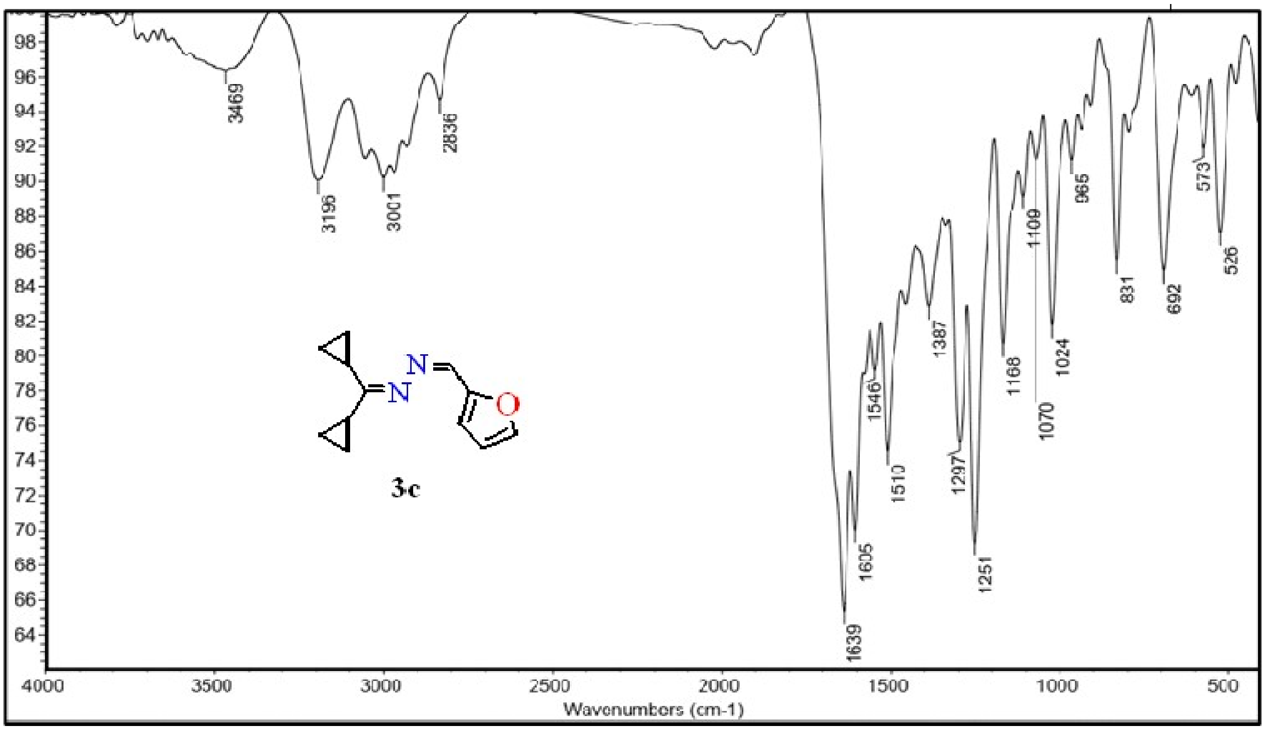

FTIR and ^{1}H-NMR (500 MHz, DMSO) spectra of compound 3c.

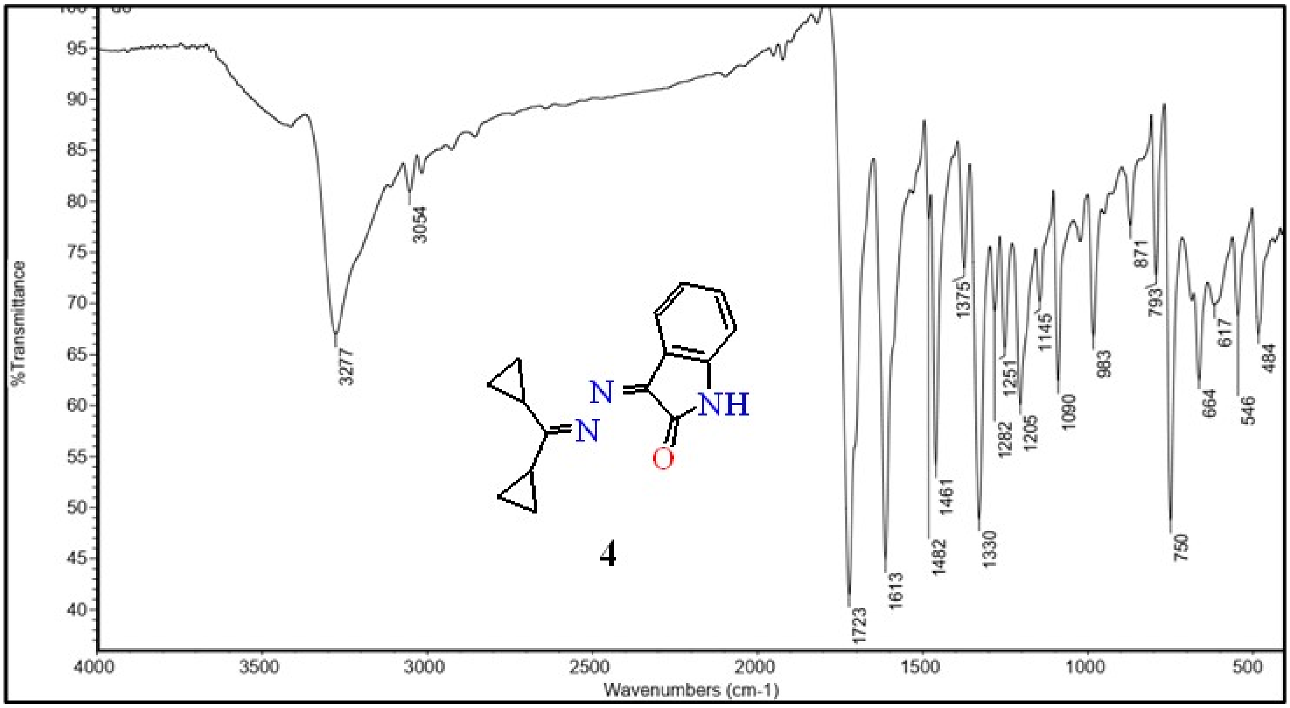
%Transmittance
Wavenumbers (cm-1)
3277
3054
1723
1613
1482
1461
1375
1330
1282
1251
1205
1145
1090
983
871
793
750
664
617
546
484
N
N
NH
O
4

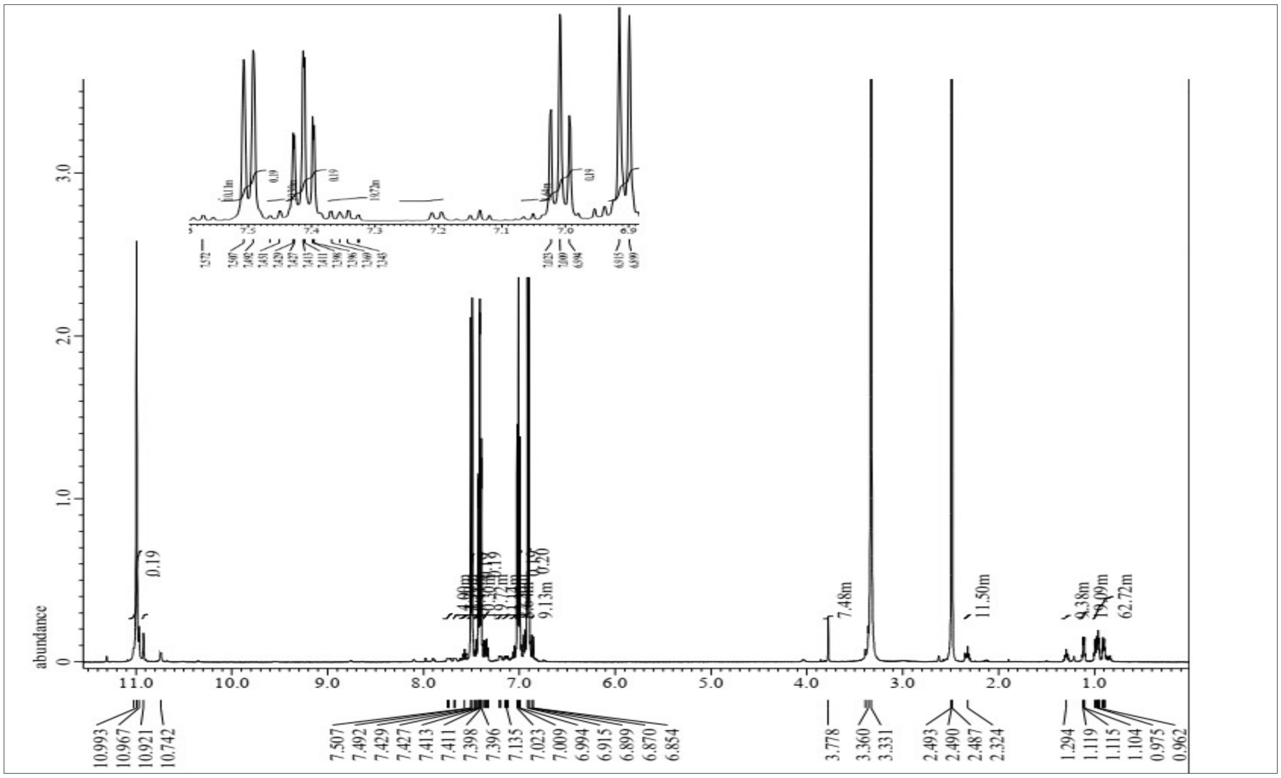
abundance
0.19
7.48m
11.50m
62.72m
9.13m
10.993
10.967
10.921
10.742
7.507
7.492
7.429
7.427
7.413
7.411
7.398
7.396
7.135
7.023
7.009
6.994
6.915
6.899
6.870
6.854
3.778
3.360
3.331
2.493
2.490
2.487
2.324
1.294
1.119
1.115
1.104
0.975
0.962

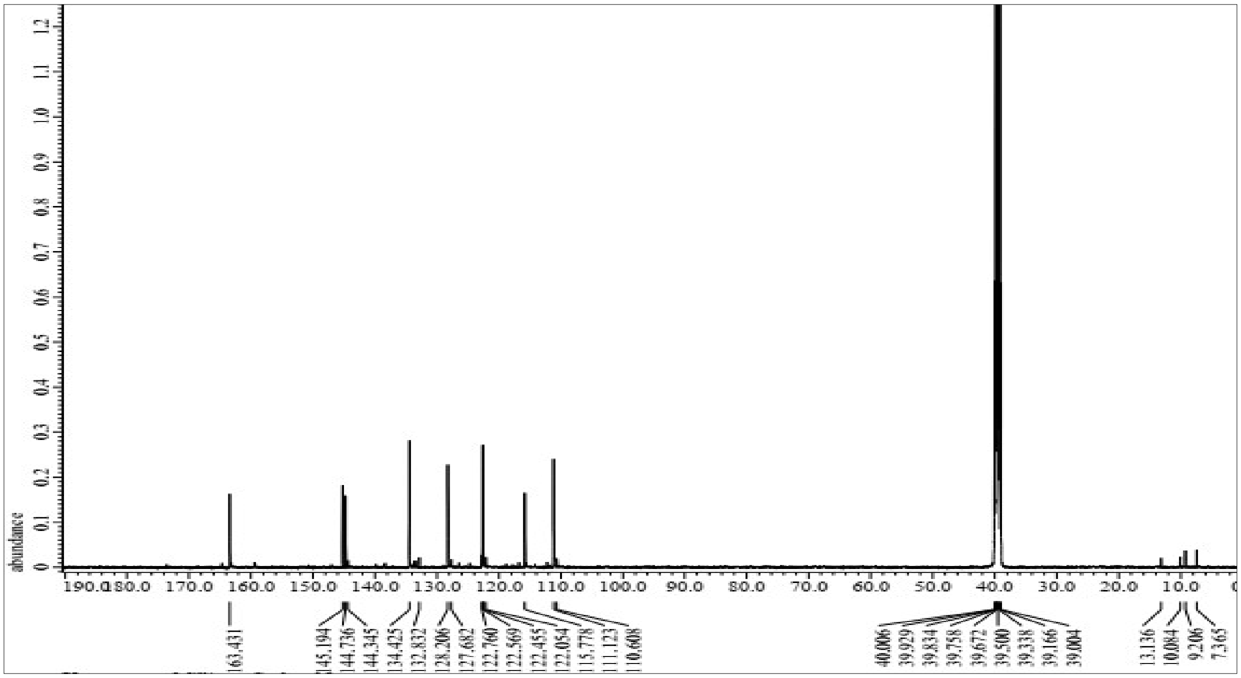

FTIR, ¹H-NMR (500 MHz, DMSO), and ¹³C-NMR (125 MHz, DMSO) spectra of compound 4.

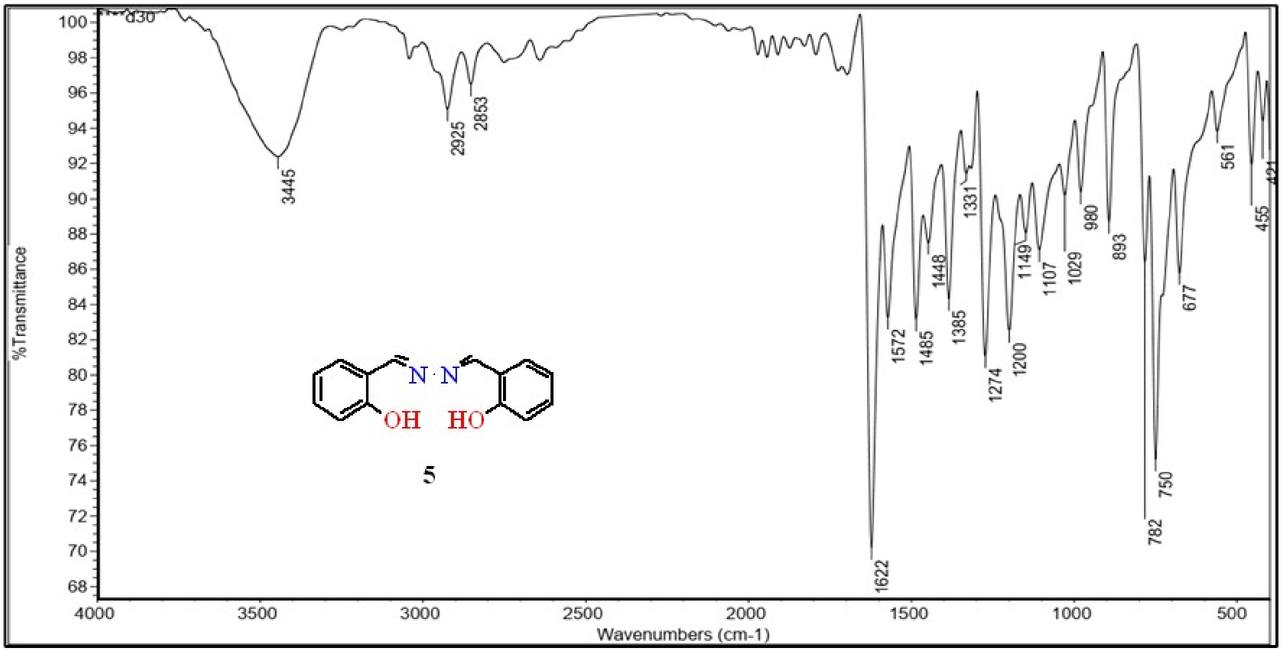

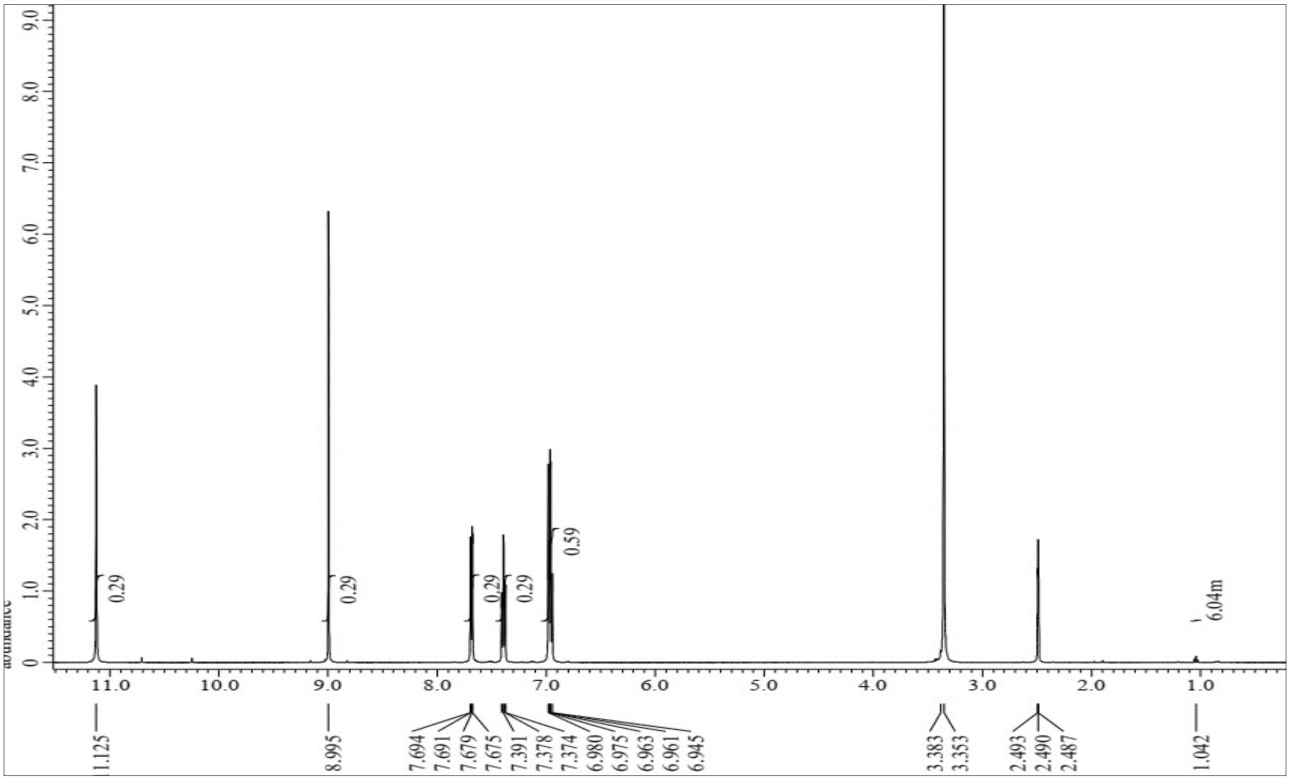

FTIR and ^{1}H-NMR (500 MHz, DMSO) spectra of compound 5.

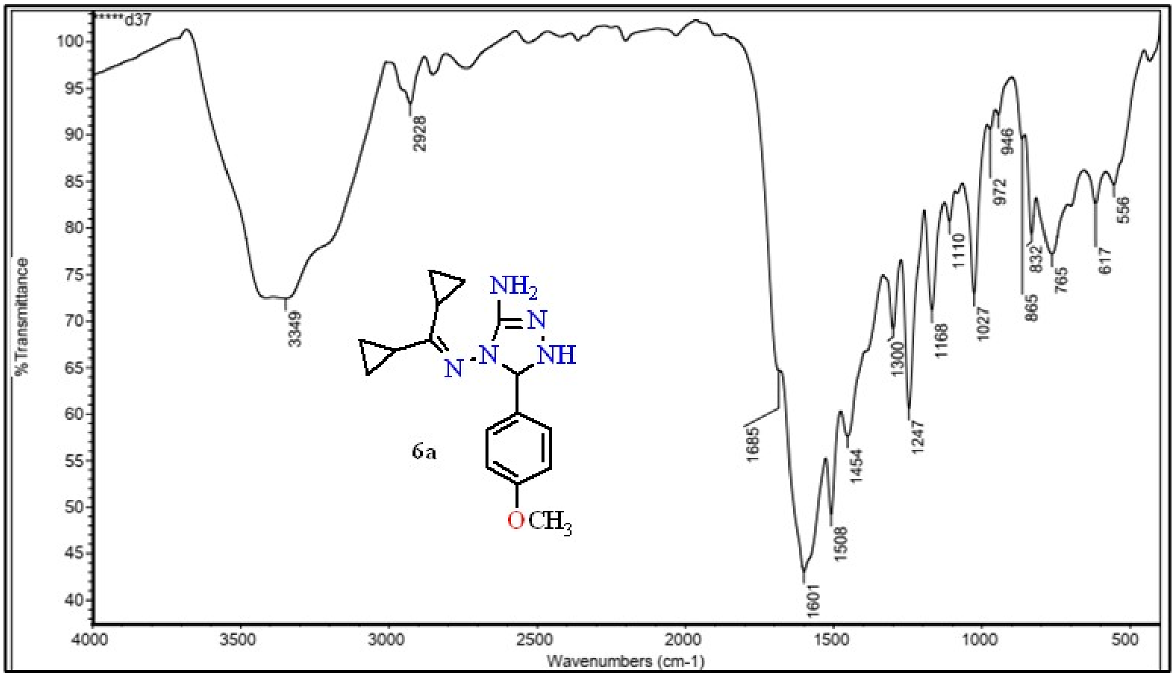

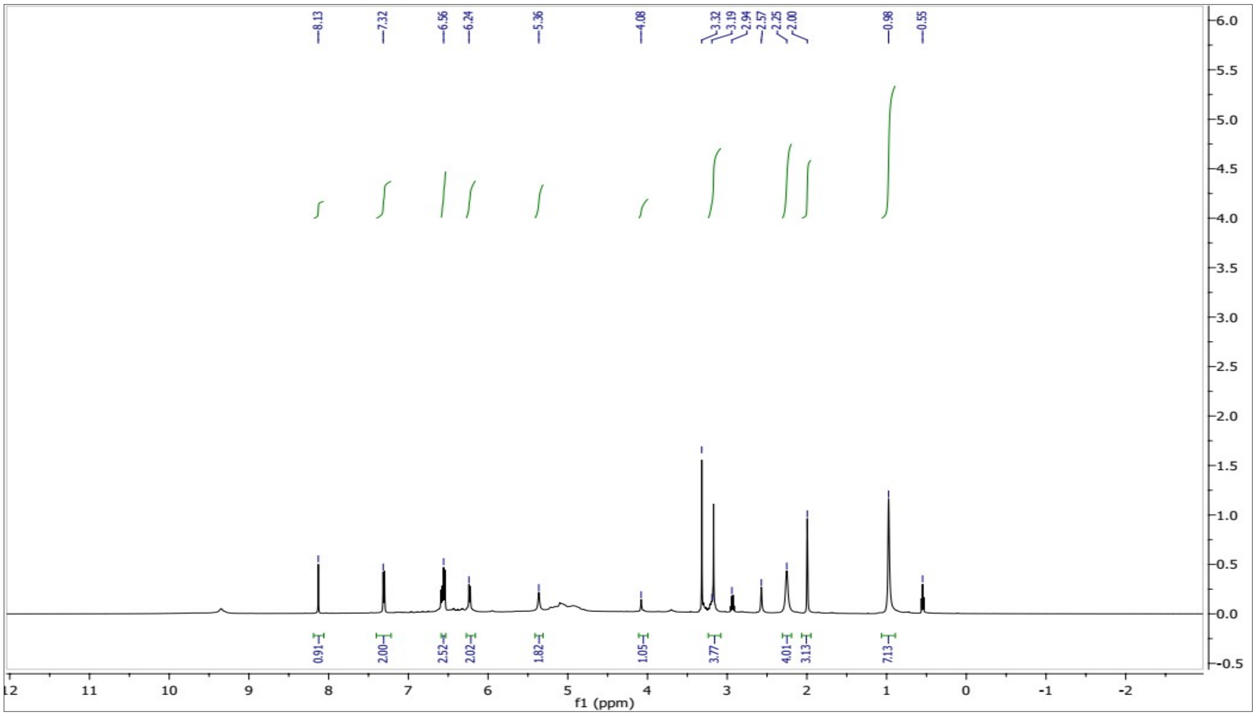

FTIR and ^{1}H-NMR (500 MHz, DMSO) spectra of compound 6a.

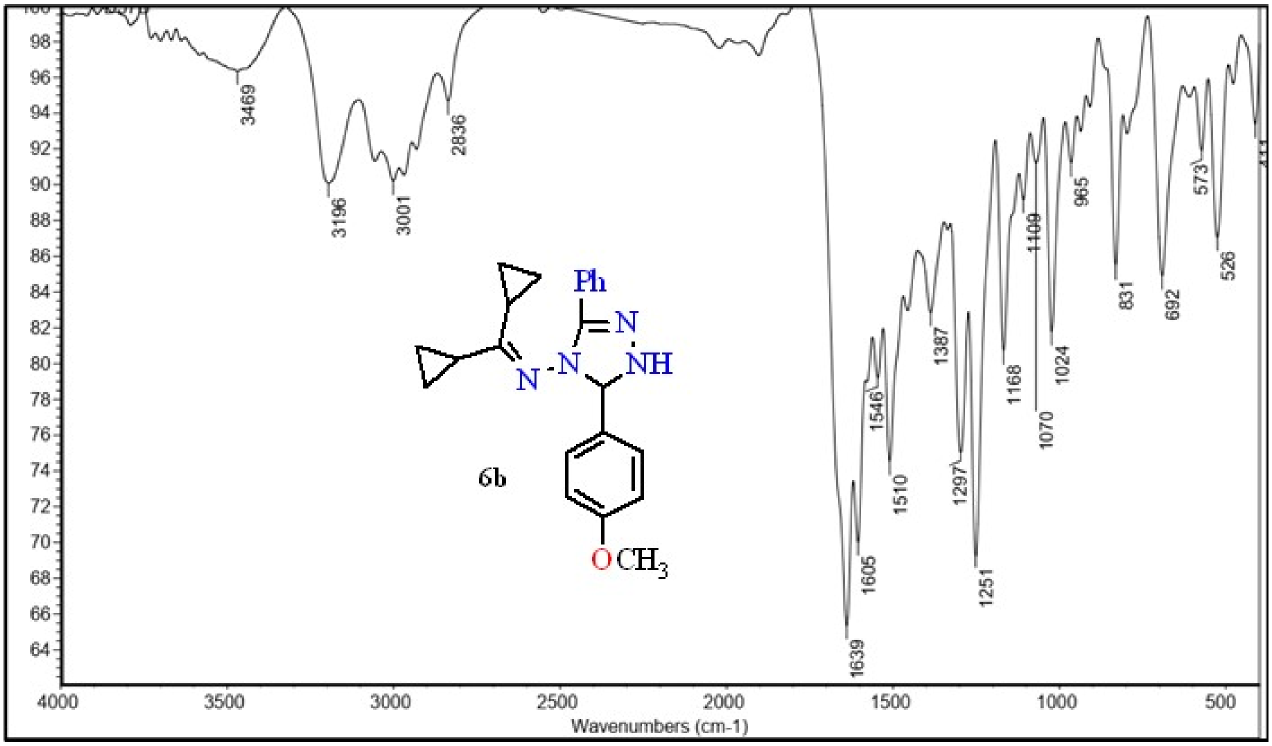

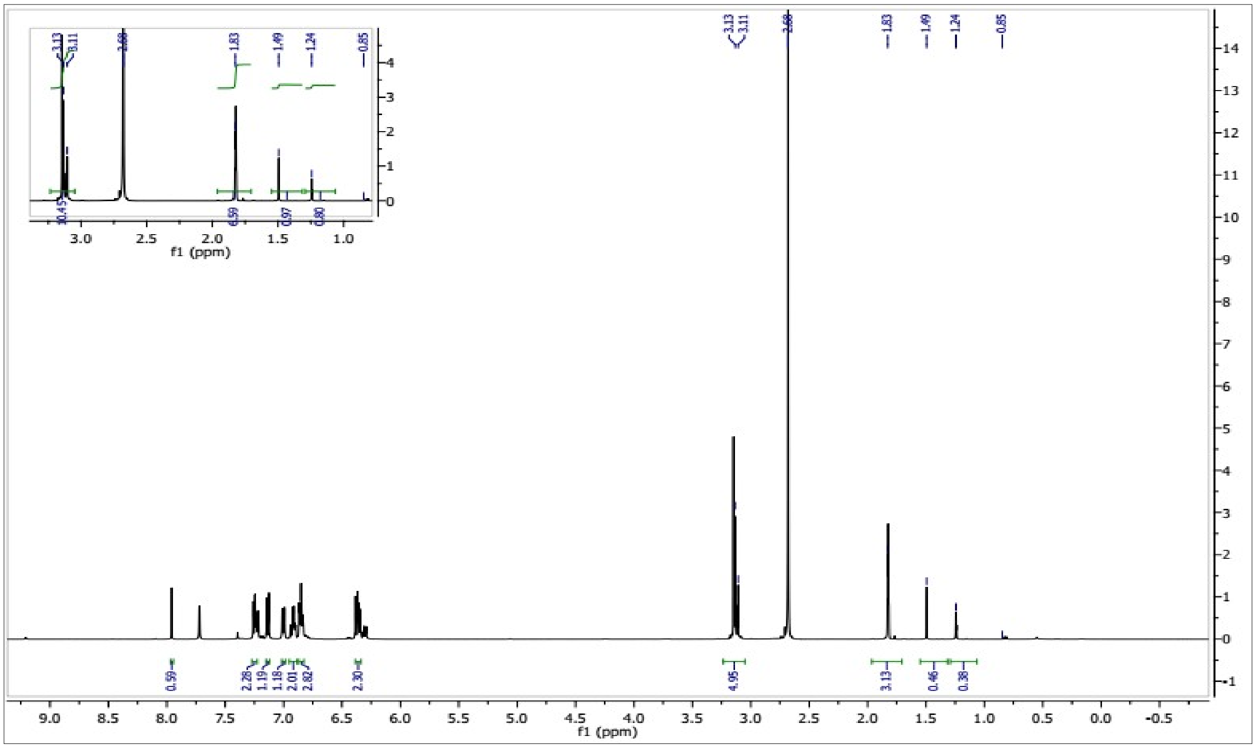

FTIR and ^{1}H-NMR (500 MHz, DMSO) spectra of compound 6b.

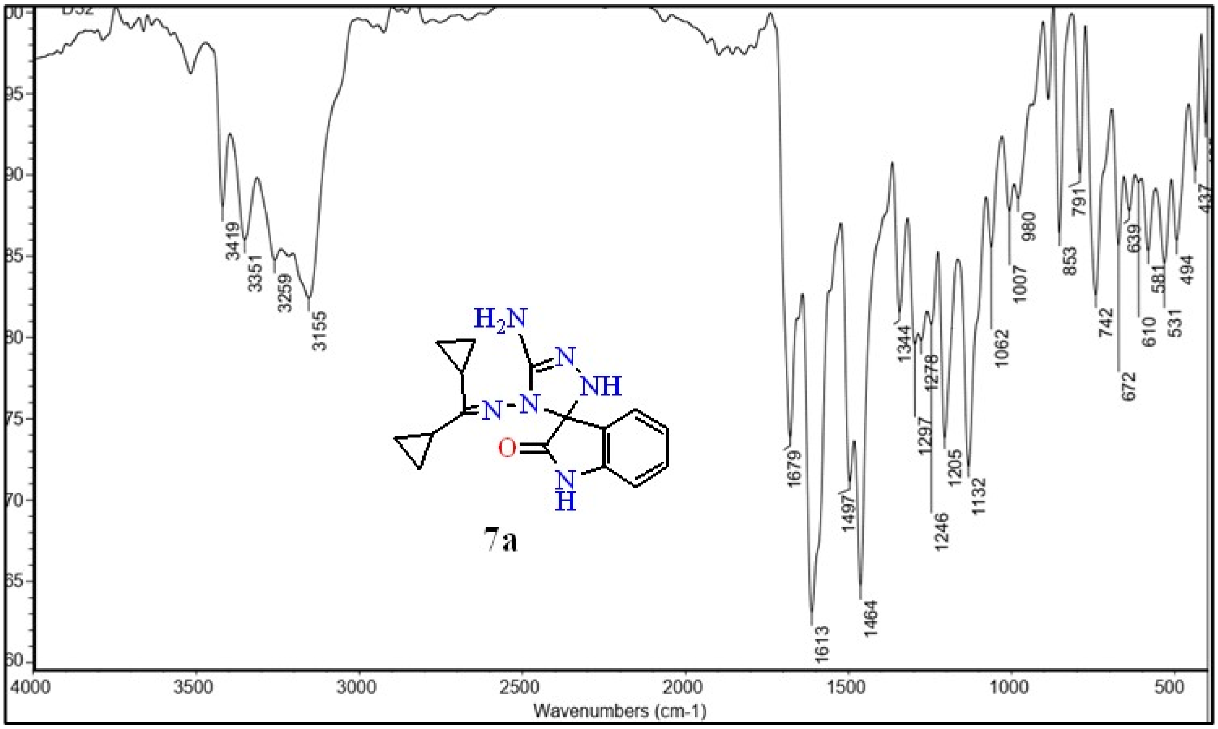

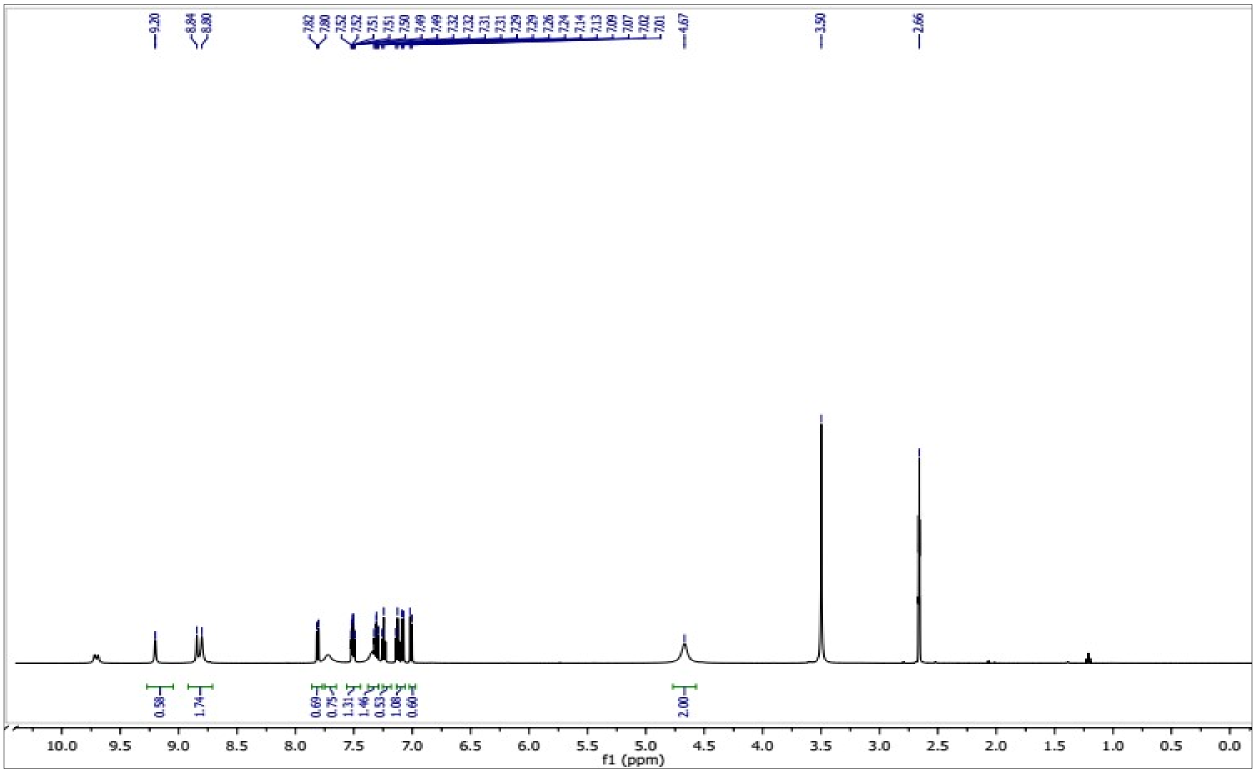

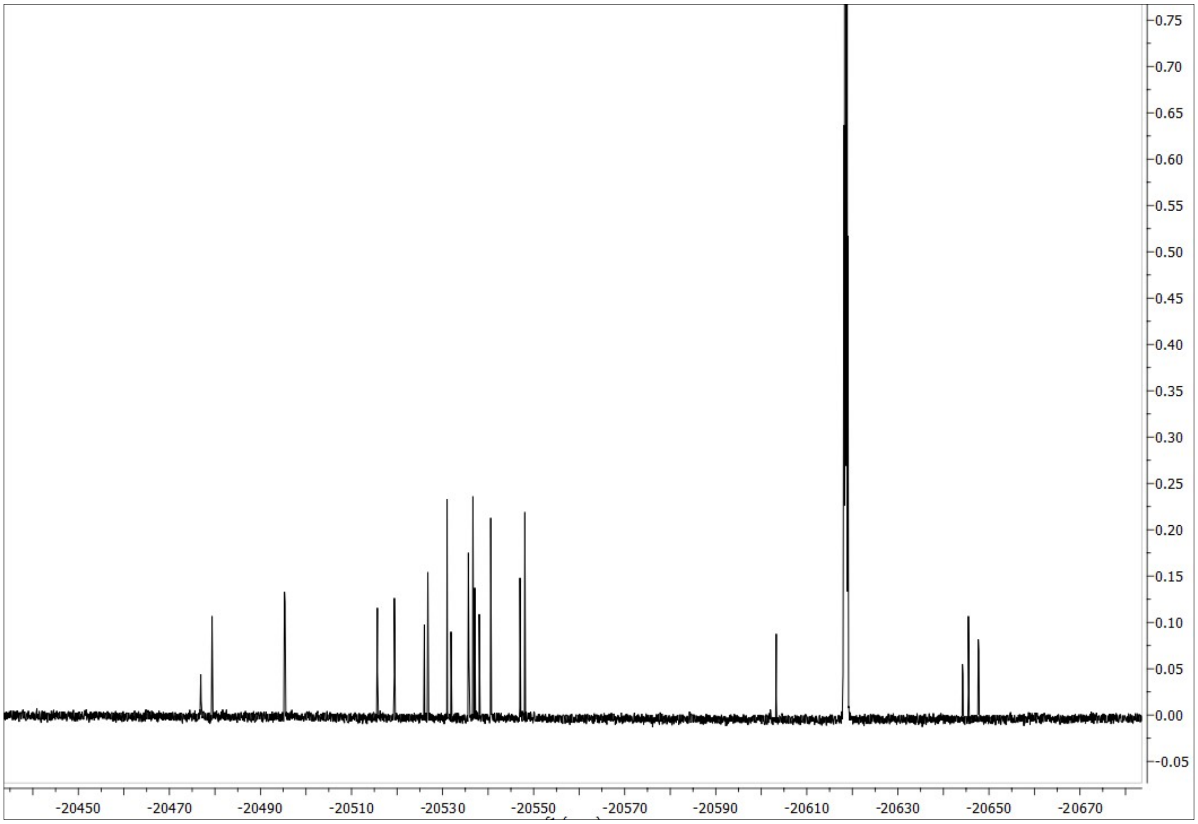

FTIR, ^{1}H-NMR (500 MHz, DMSO), and ^{13}C-NMR (125 MHz, DMSO) spectra of compound 7a.

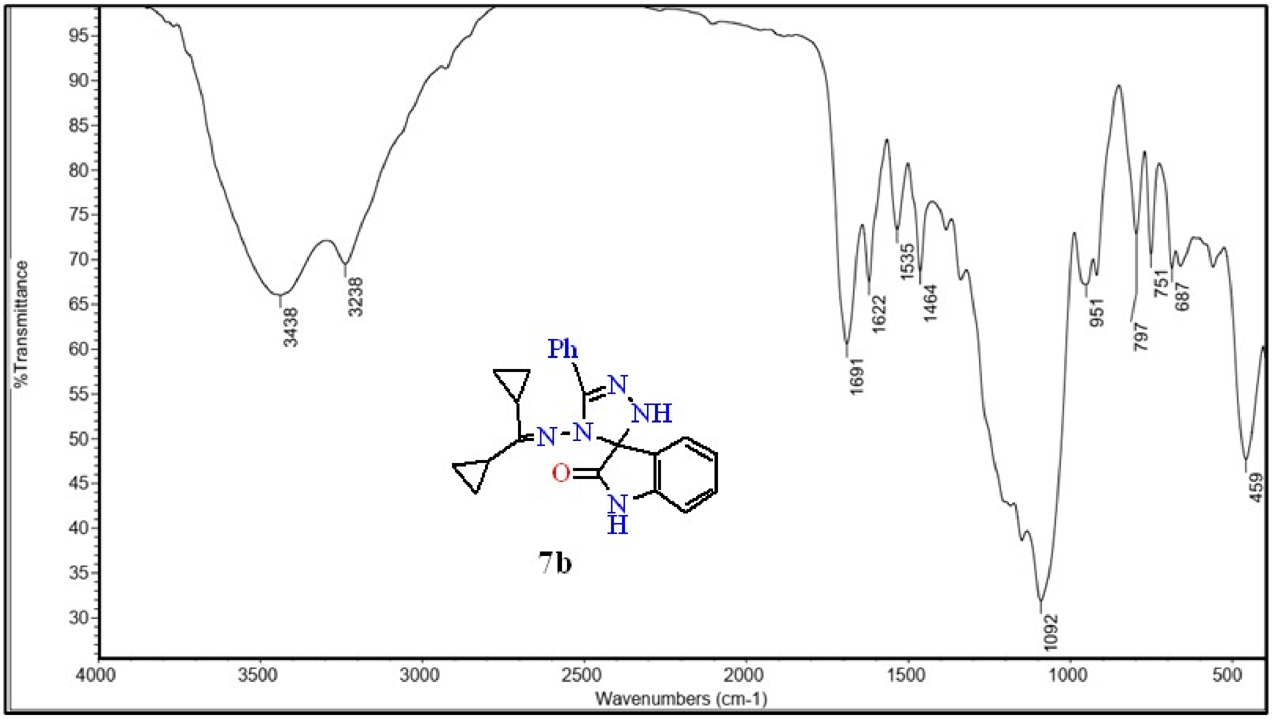
%Transmittance
Wavenumbers (cm-1)
3438
3238
1691
1622
1535
1464
1092
951
797
751
687
459
Ph
N
NH
N
N
O
N
H
7b

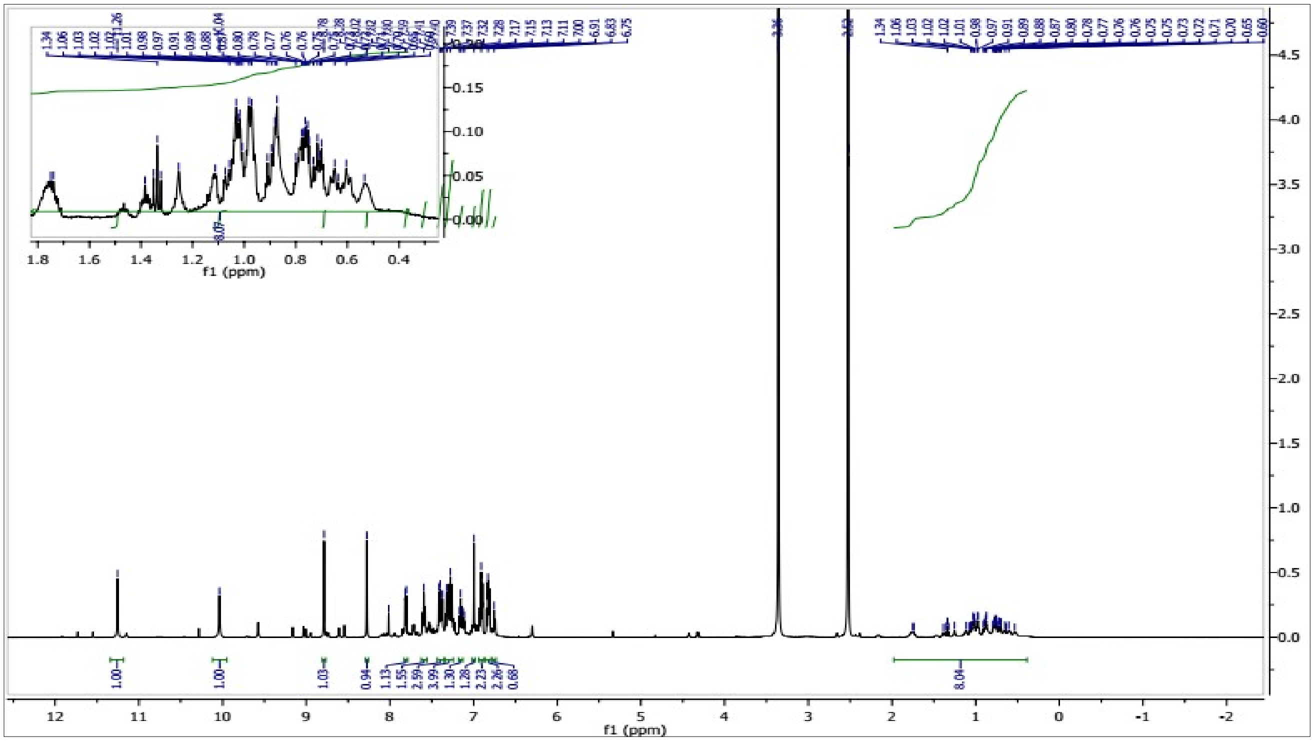
f1 (ppm)

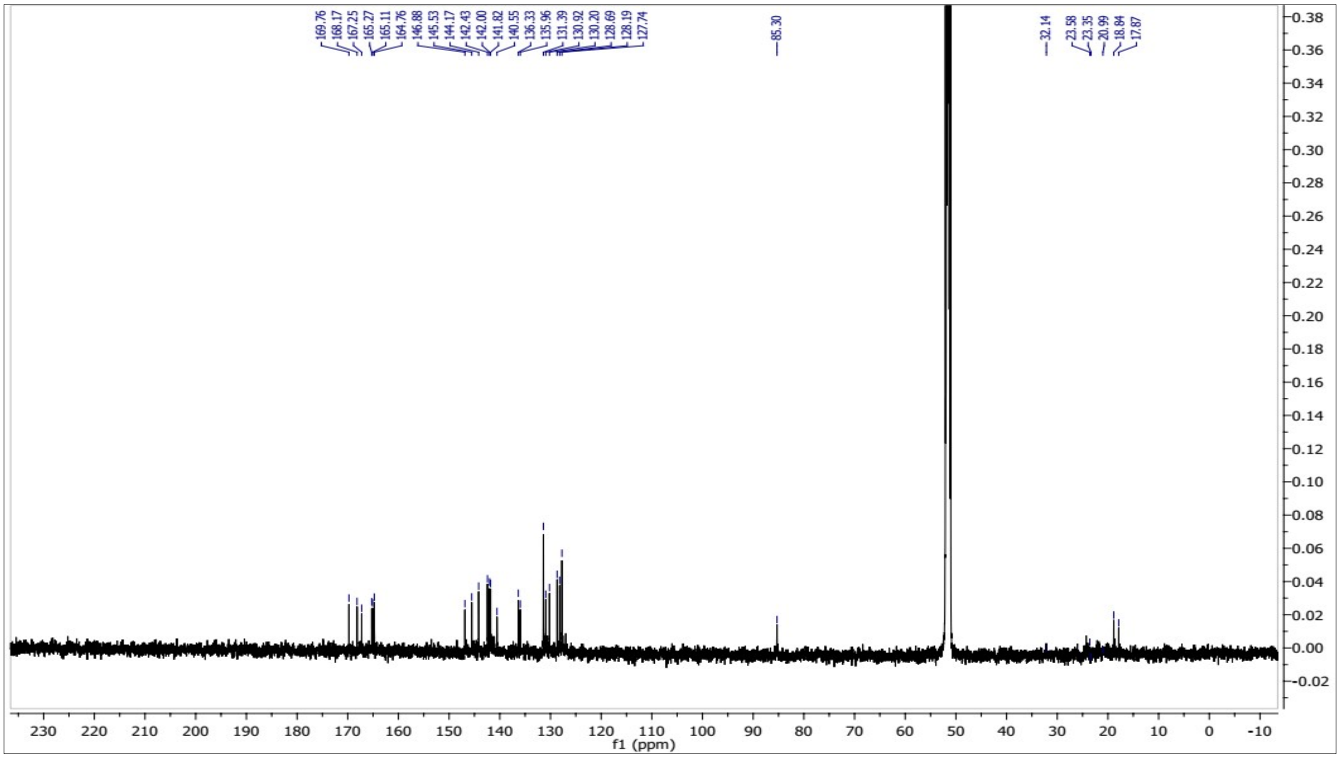

FTIR, ^{1}H-NMR (500 MHz, DMSO), and ^{13}C-NMR (125 MHz, DMSO) spectra of compound 7b.

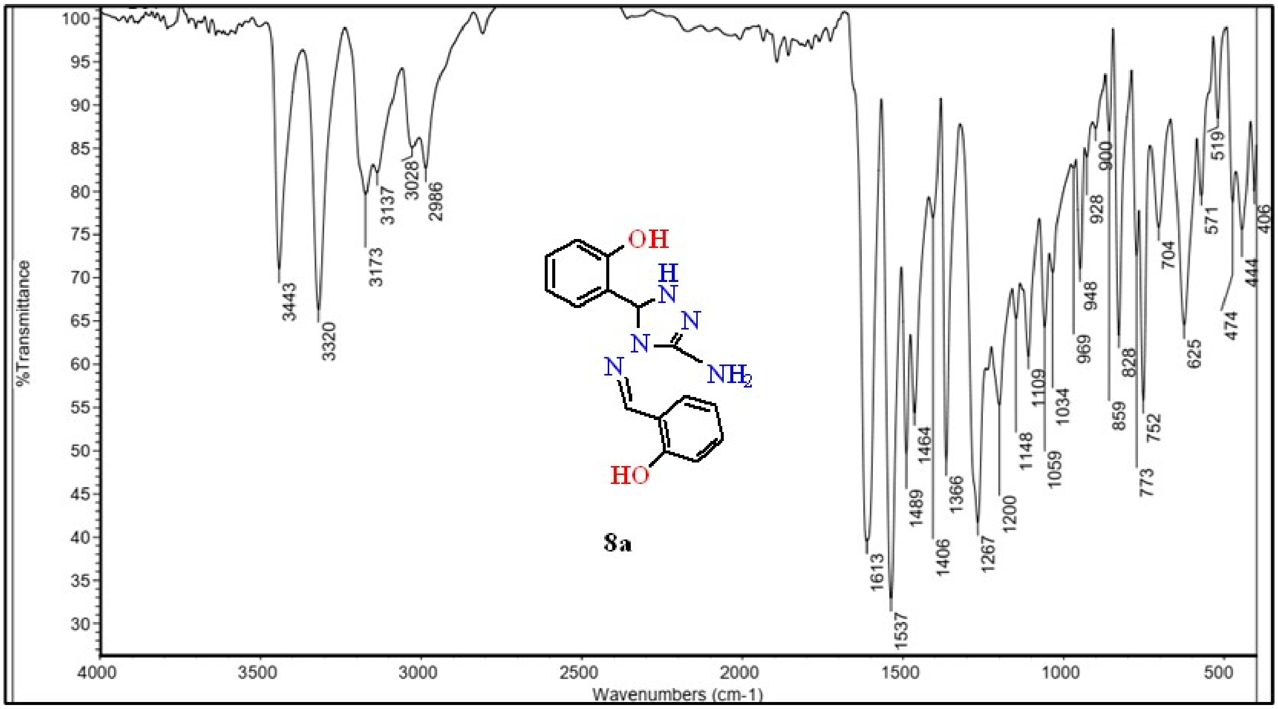

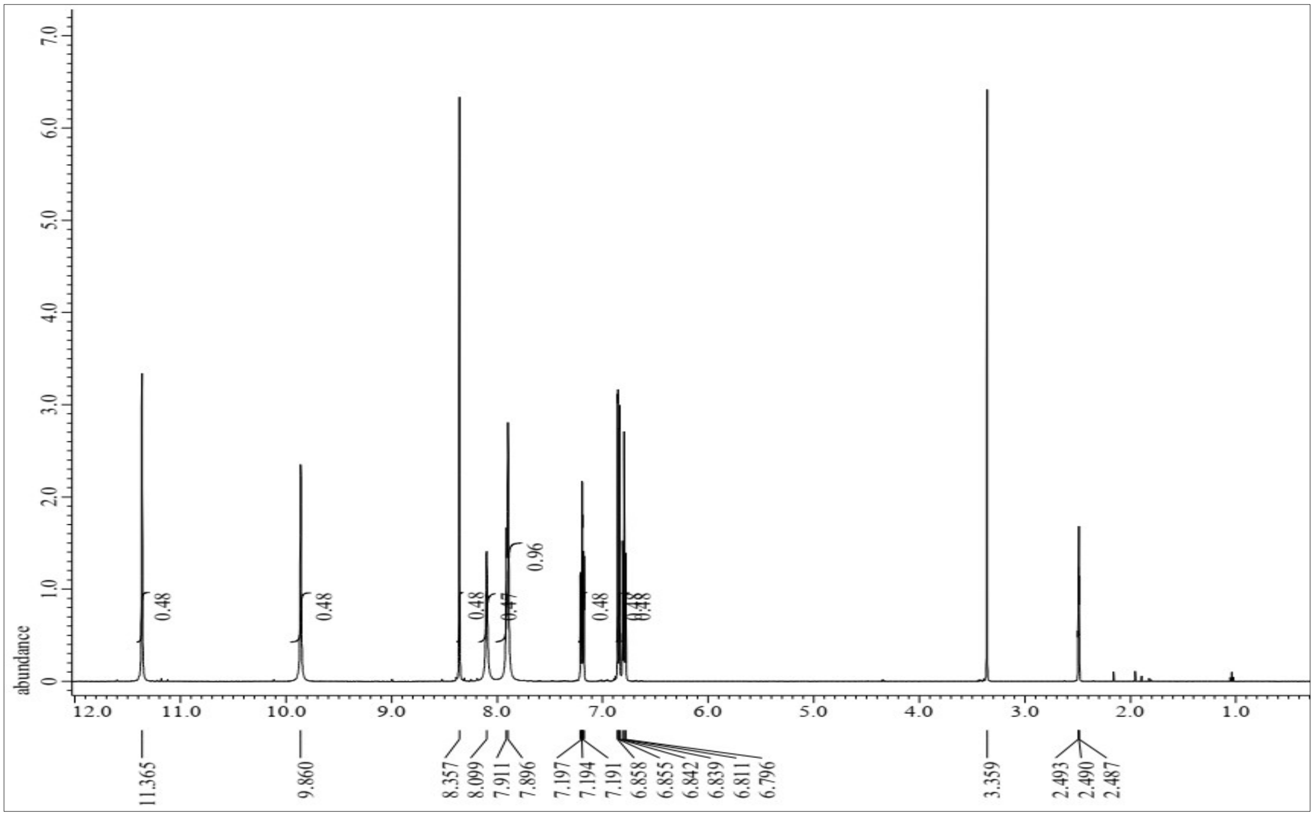

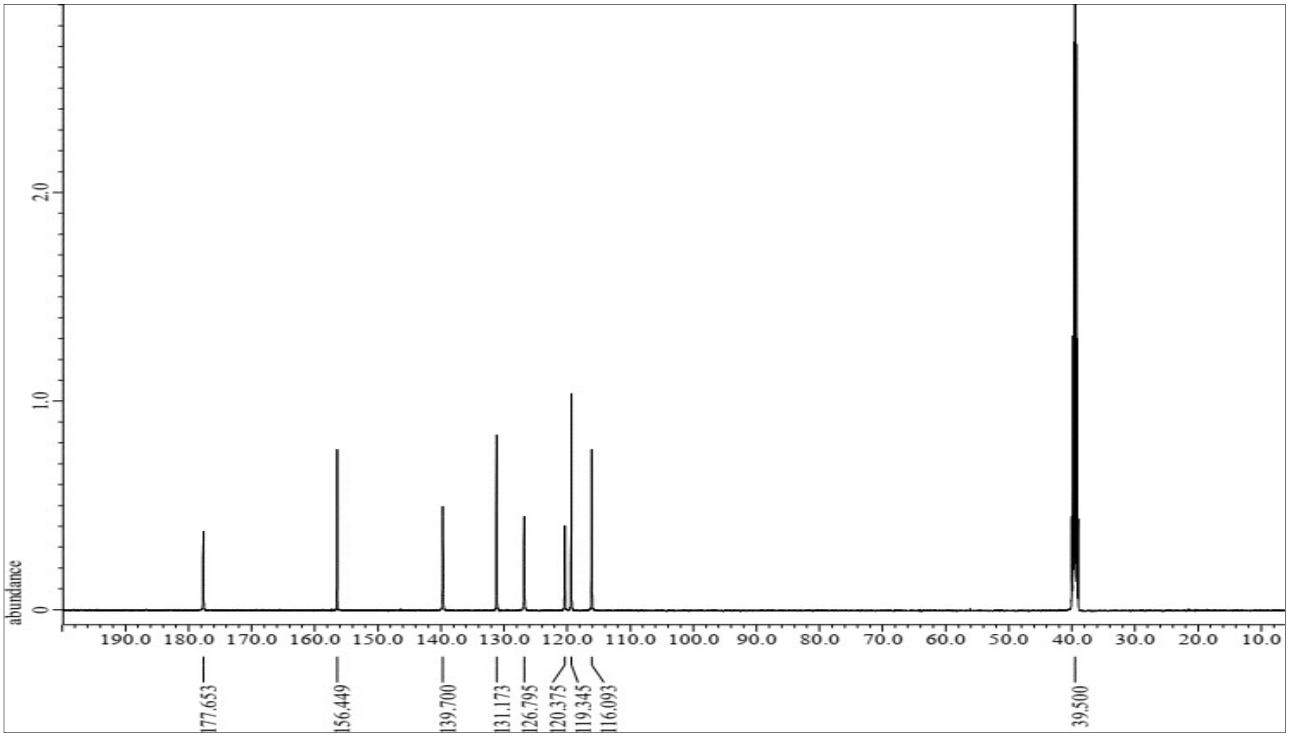

FTIR, ^{1}H-NMR (500 MHz, DMSO), and ^{13}C-NMR (125 MHz, DMSO) spectra of compound 8a.

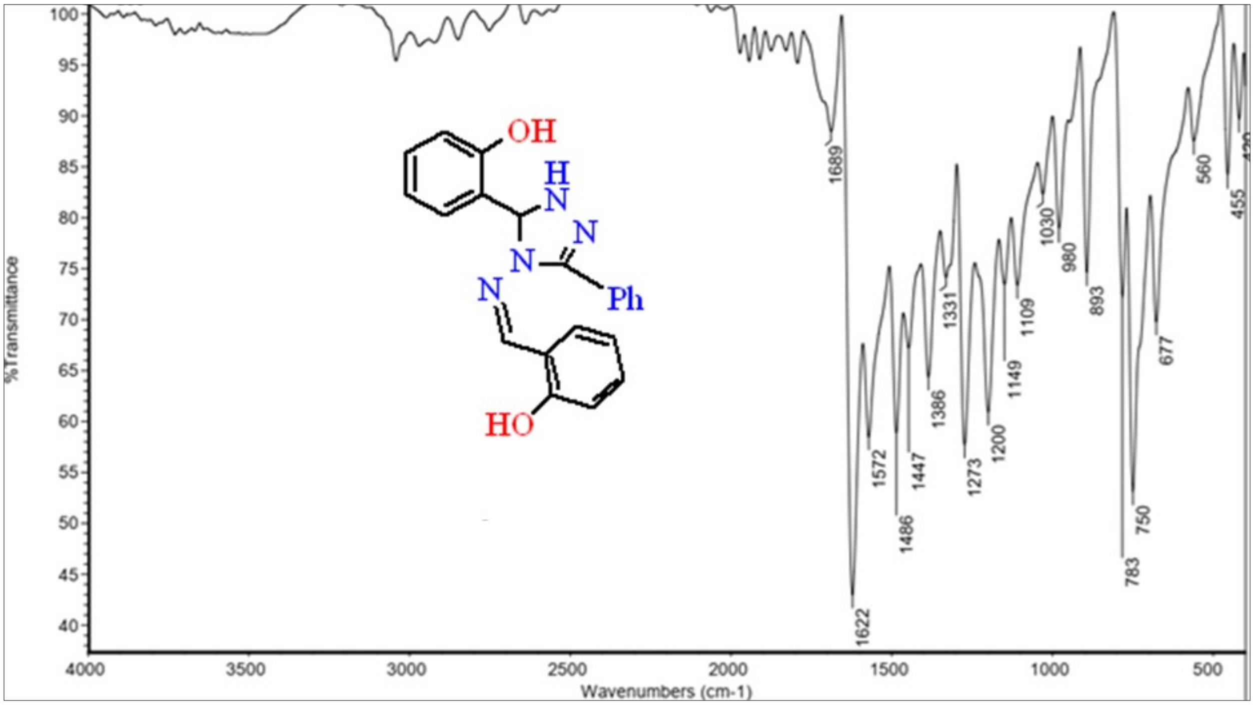

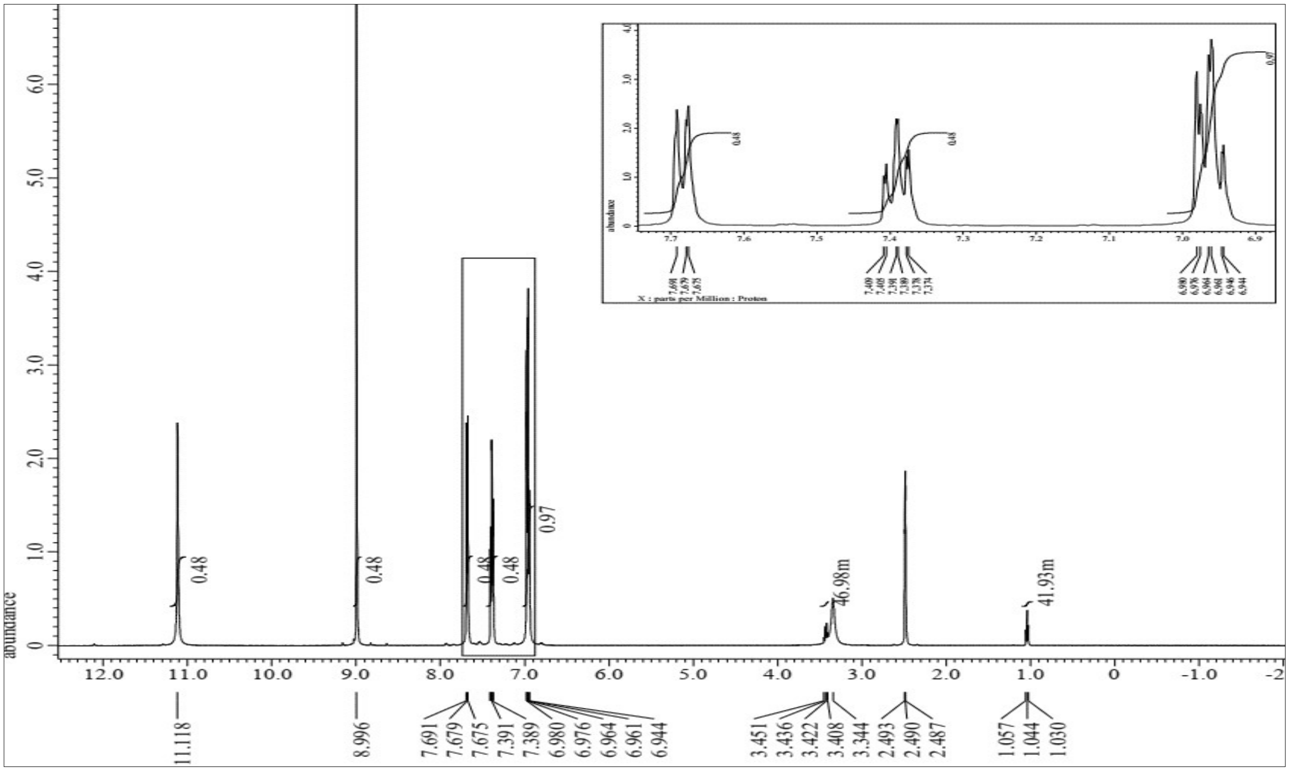

FTIR, and^{1}H-NMR (500 MHz, DMSO) of compound 8b.

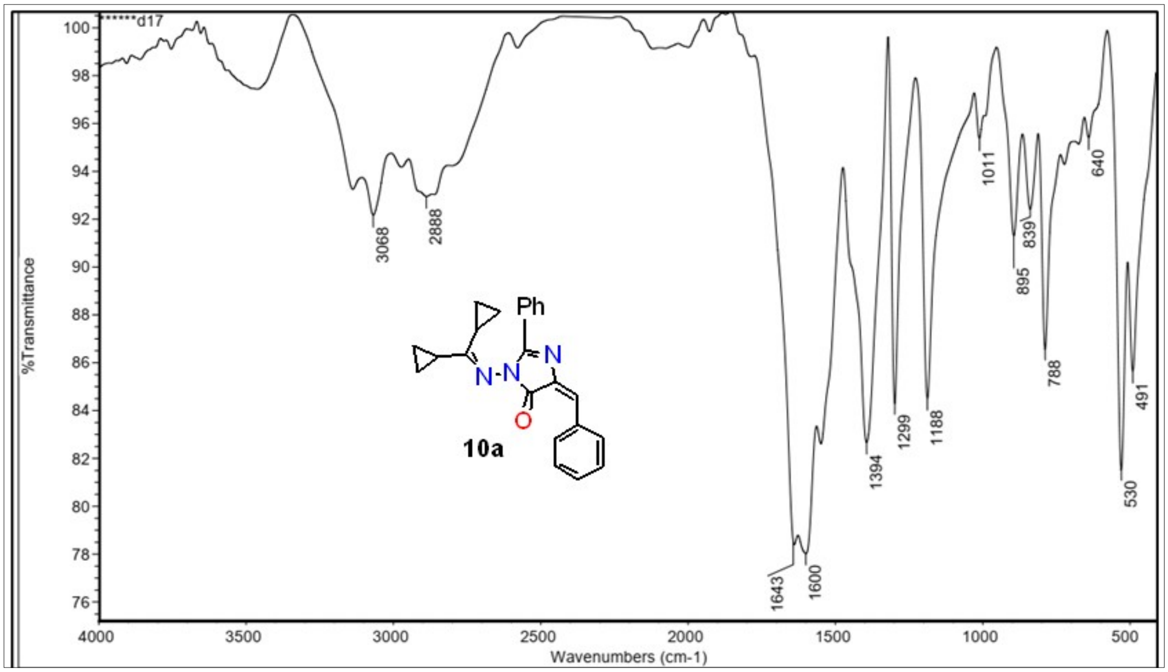
******d17
%Transmittance
Wavenumbers (cm-1)
10a
Ph
N
N
N
O
3068
2888
1643
1600
1394
1299
1188
1011
895
839
788
640
530
491

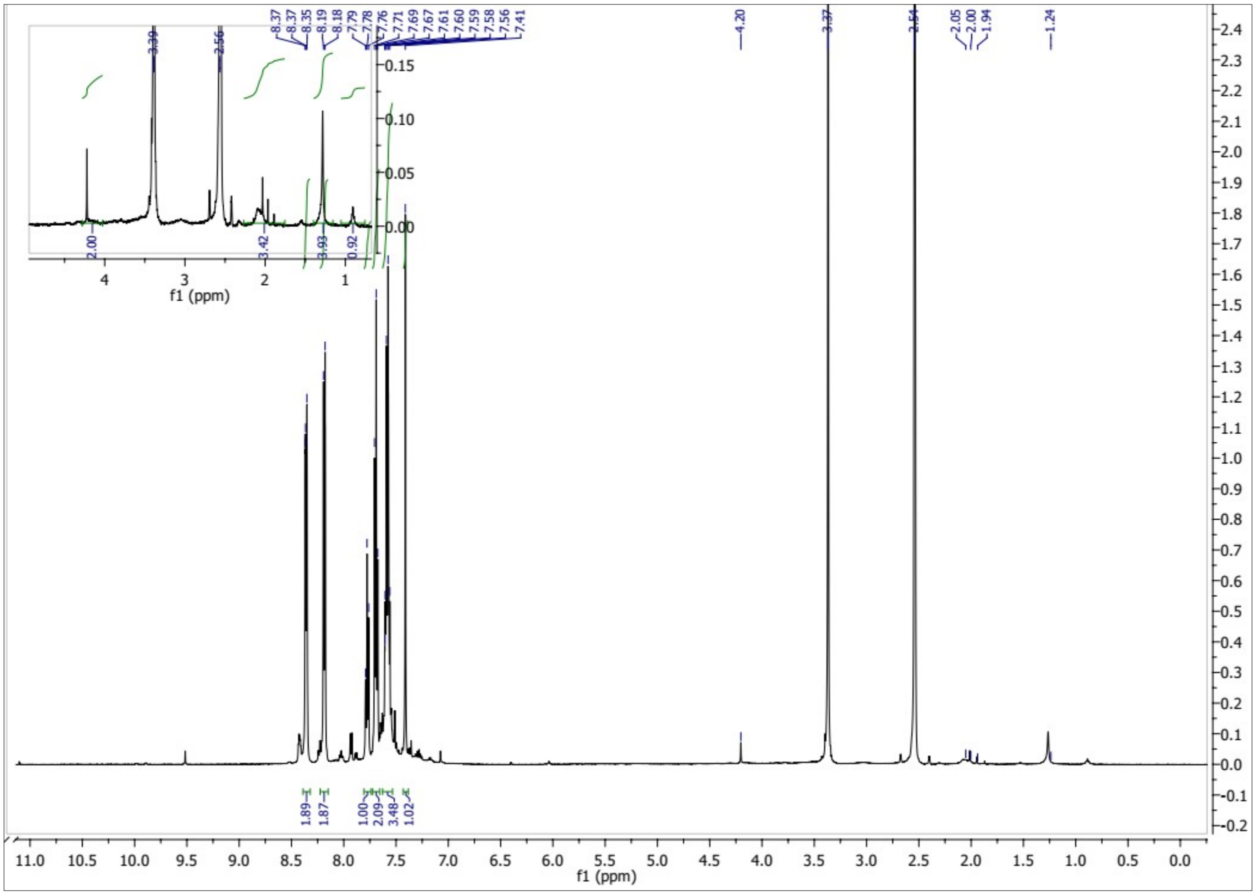
8.37
8.37
8.35
8.19
8.18
7.79
7.78
7.76
7.71
7.69
7.67
7.61
7.60
7.59
7.58
7.56
7.41
4.20
3.37
2.54
2.05
2.00
1.94
1.24
1.89
1.87
1.00
2.09
3.48
1.02
3.39
2.56
2.00
3.42
3.93
0.92
f1 (ppm)

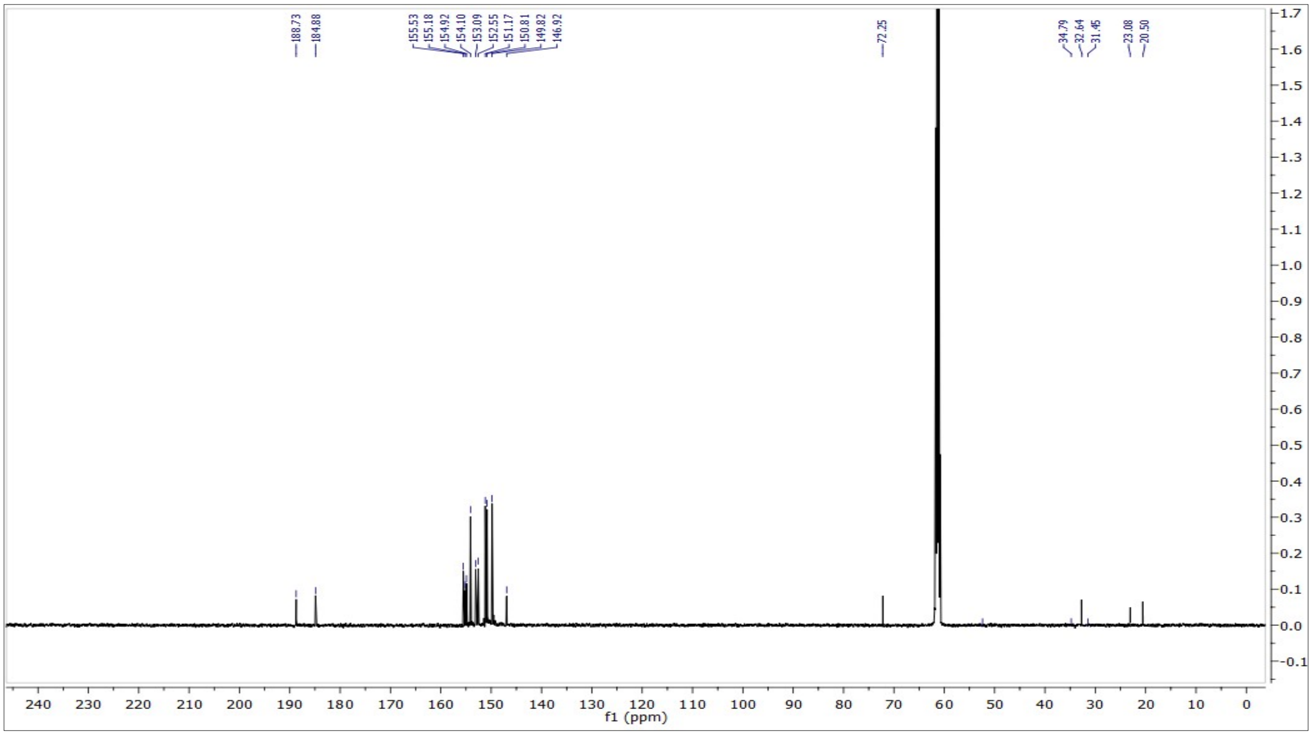

FTIR, ^{1}H-NMR (500 MHz, DMSO), and ^{13}C-NMR (125 MHz, DMSO) of compound 10a.

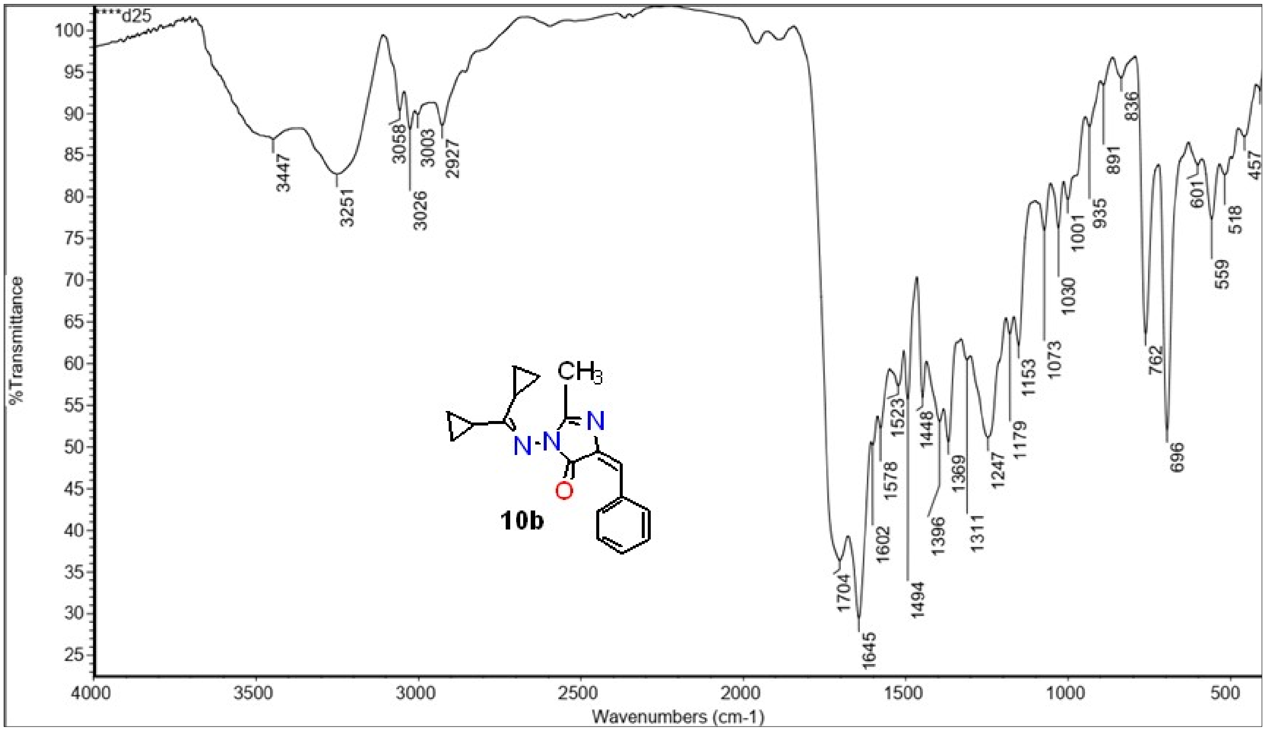

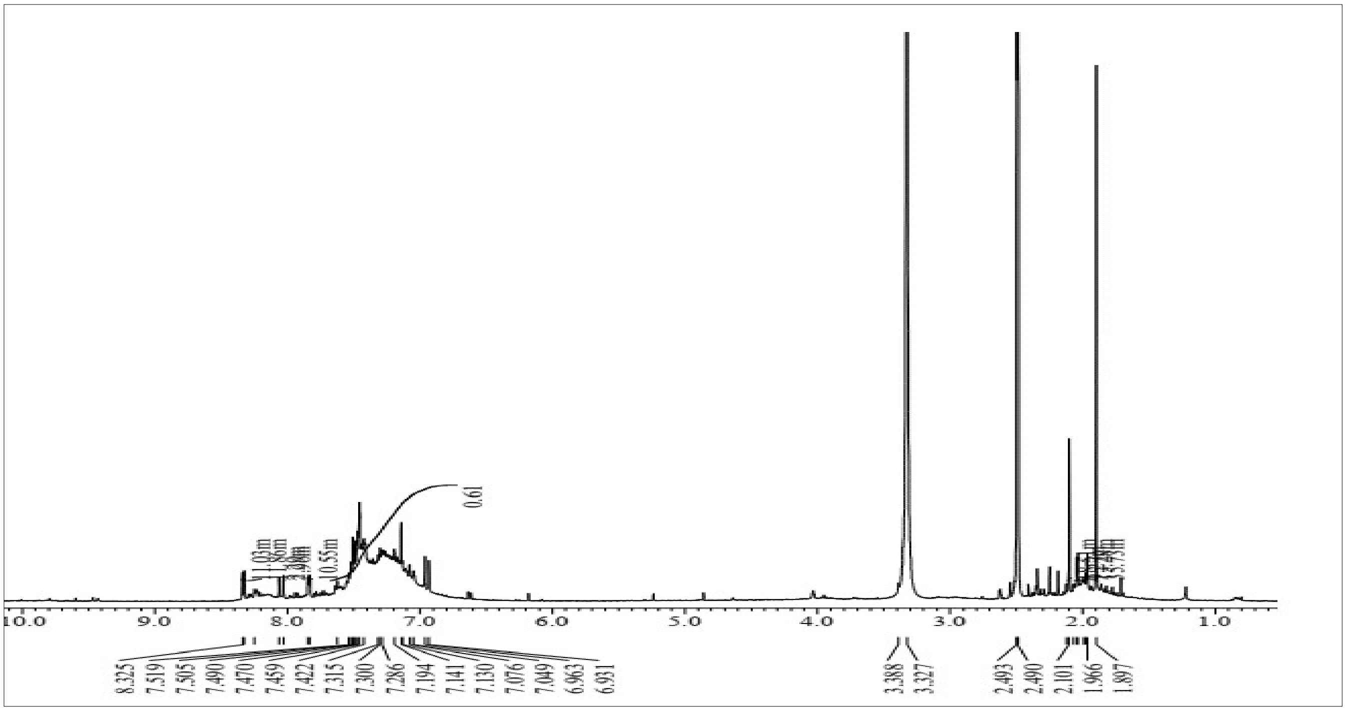

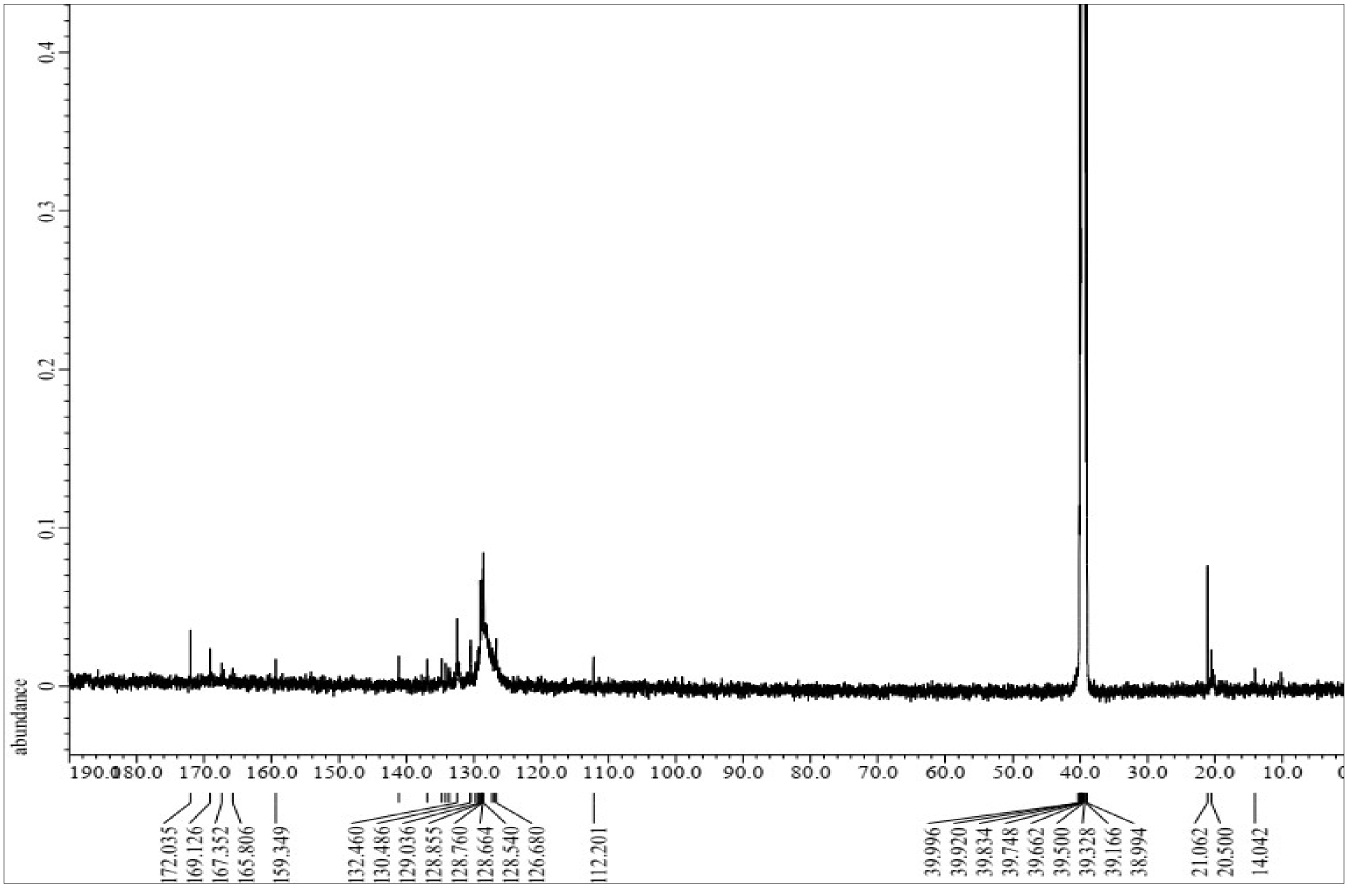

FTIR, ^{1}H-NMR (500 MHz, DMSO), and ^{13}C-NMR (125 MHz, DMSO) of compound 10b.

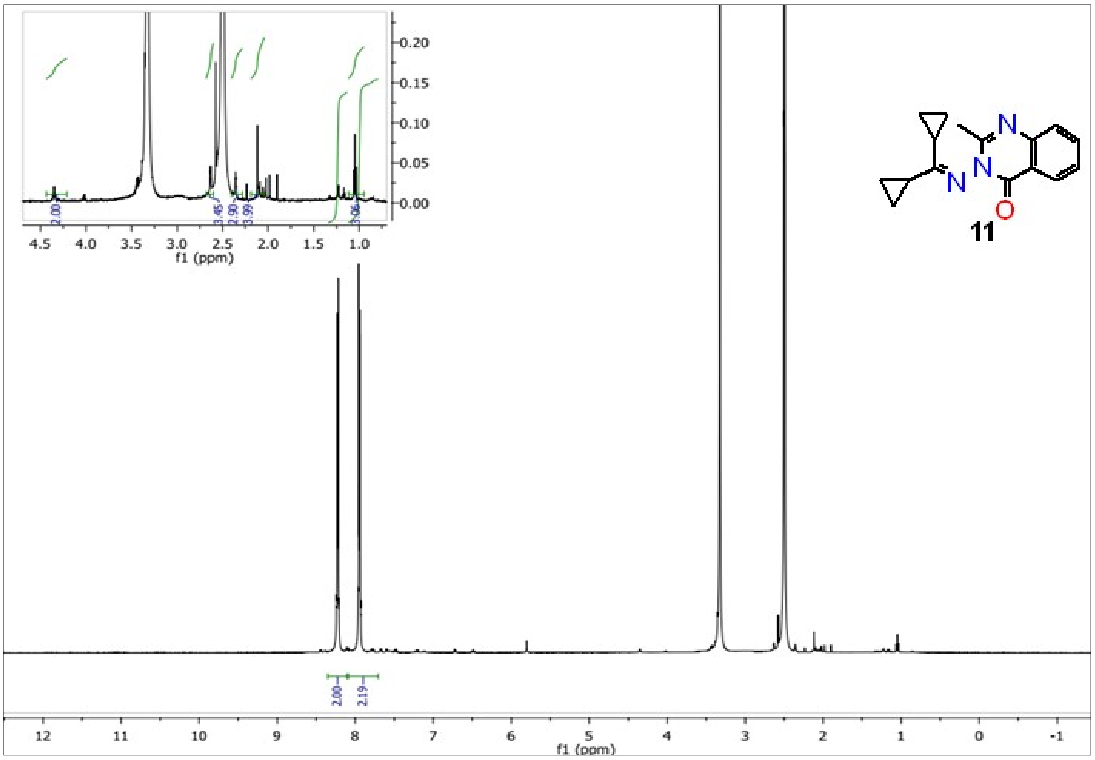

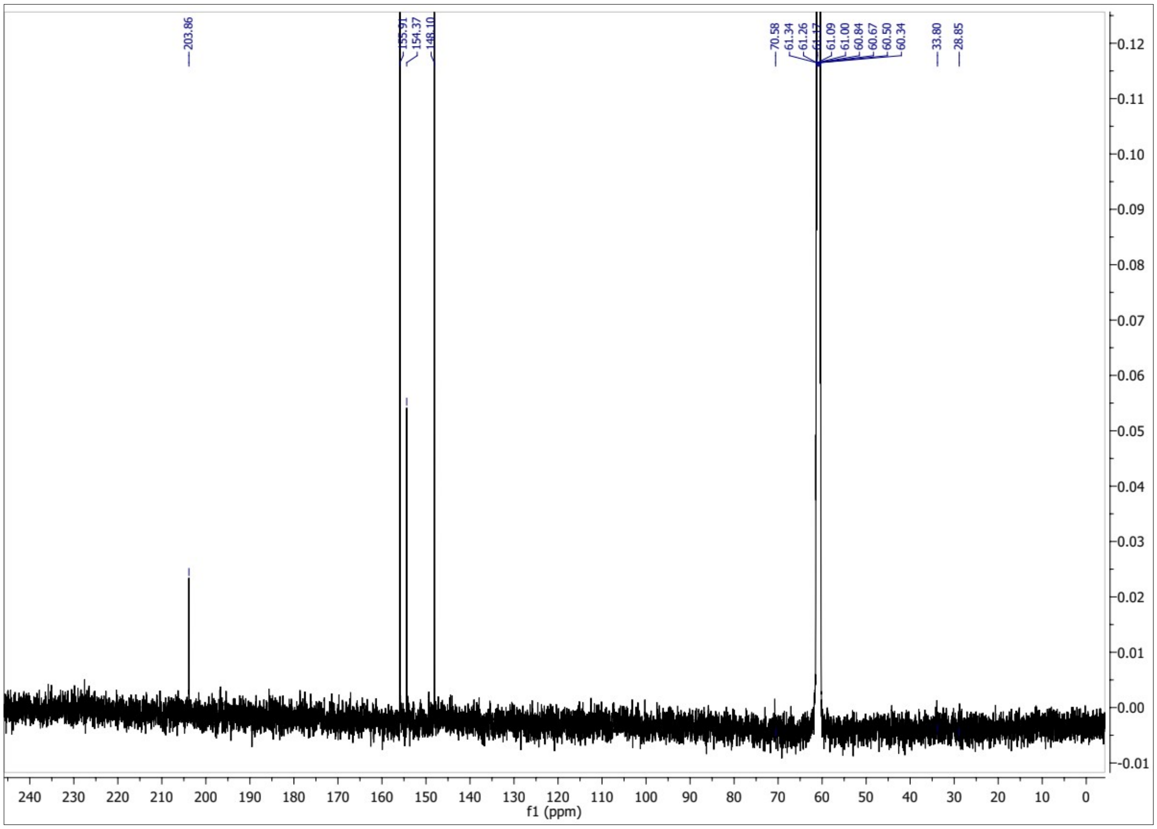

^{1}H-NMR (500 MHz, DMSO), and ^{13}C-NMR (125 MHz, DMSO) of compound 11.

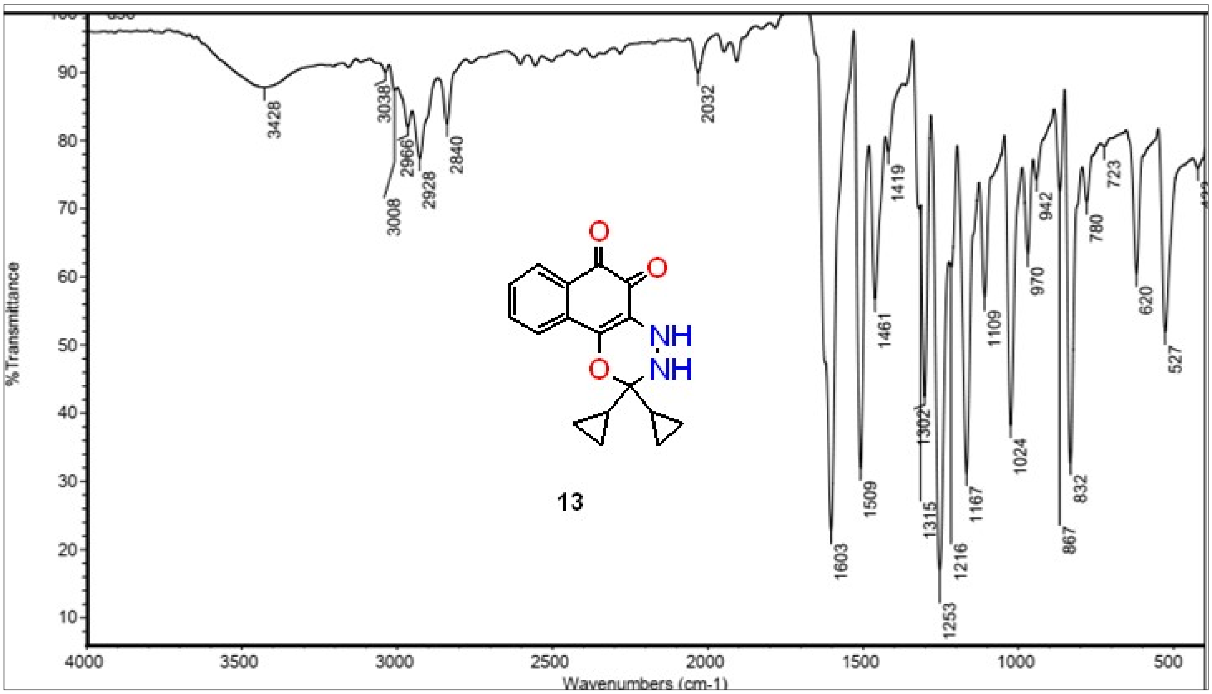
% Transmittance
Wavenumbers (cm-1)
3428
3038
3008
2966
2928
2840
2032
1603
1509
1461
1419
1315
1302
1253
1216
1167
1109
1024
970
942
867
832
780
723
620
527
O
O
NH
NH
O
13

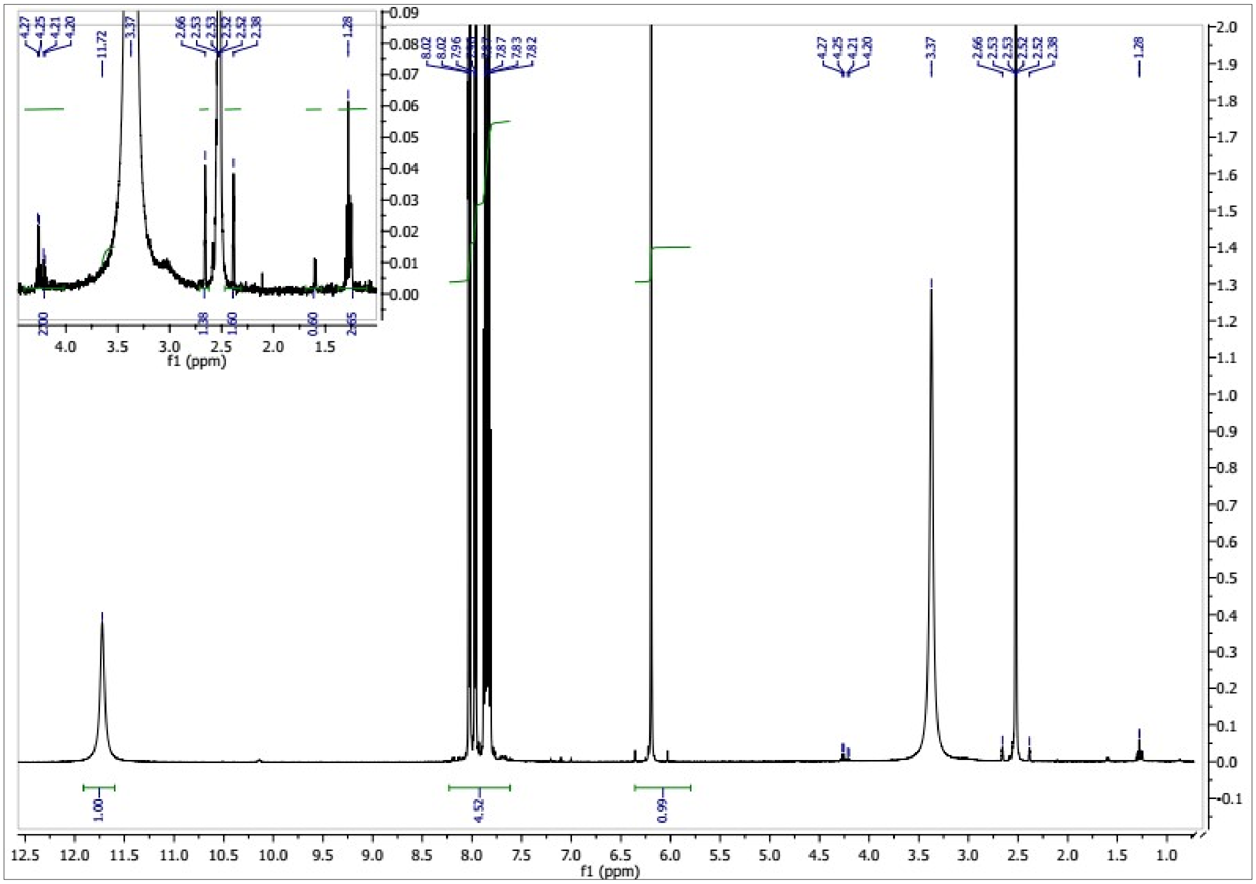
f1 (ppm)
11.72
8.02
8.02
7.96
7.87
7.83
7.82
4.27
4.25
4.21
4.20
3.37
2.66
2.53
2.53
2.52
2.52
2.38
1.28
1.00
4.52
0.99
2.00
1.38
1.60
0.60
2.65

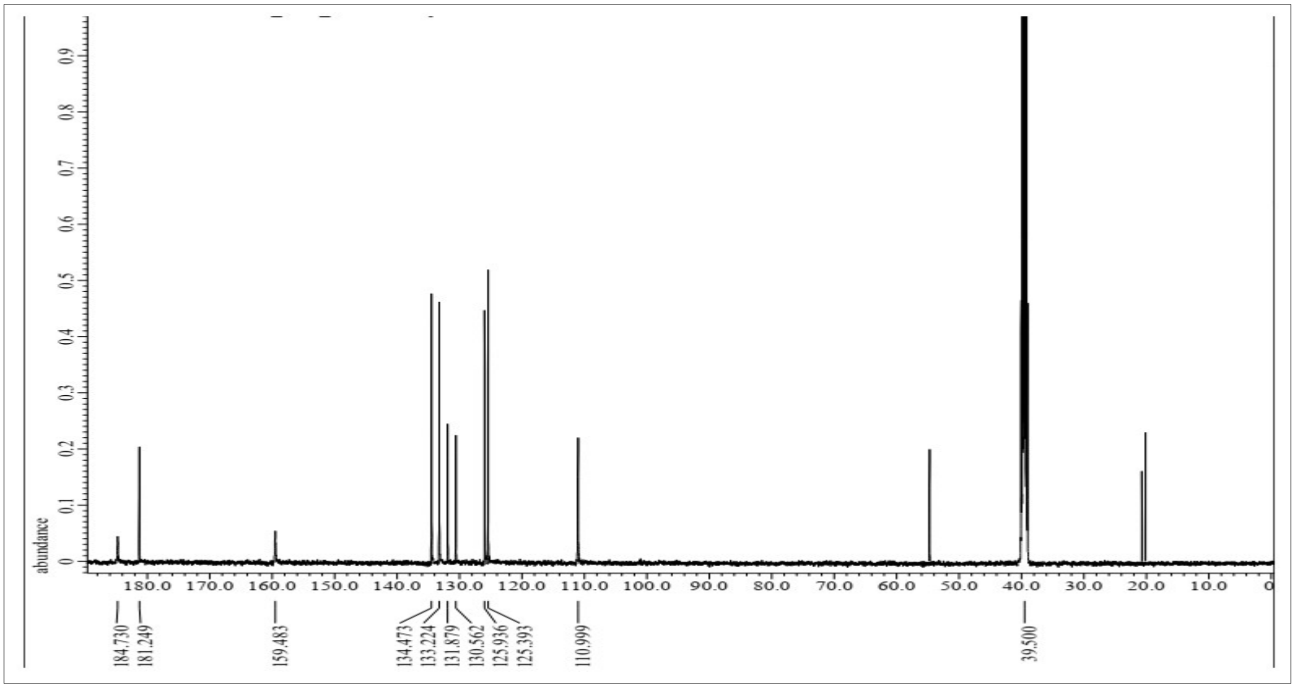

FTIR, ^{1}H-NMR (500 MHz, DMSO), and ^{13}C-NMR (125 MHz, DMSO) of compound 13.

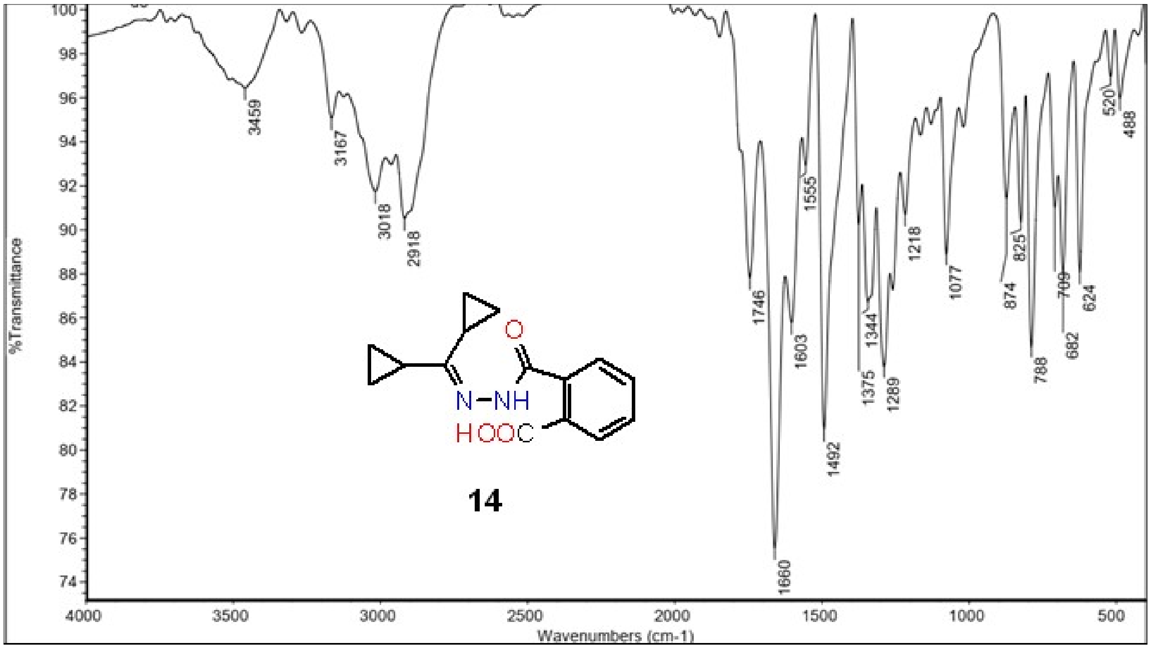

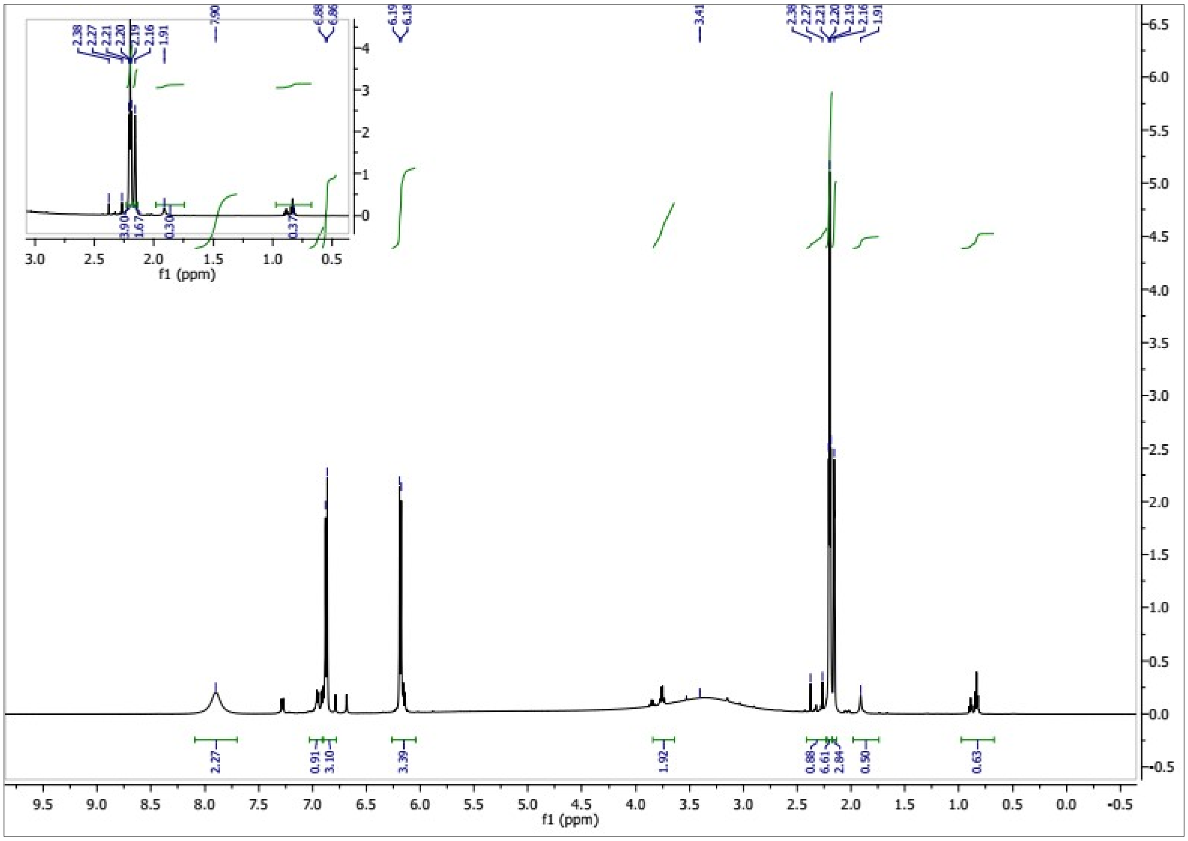

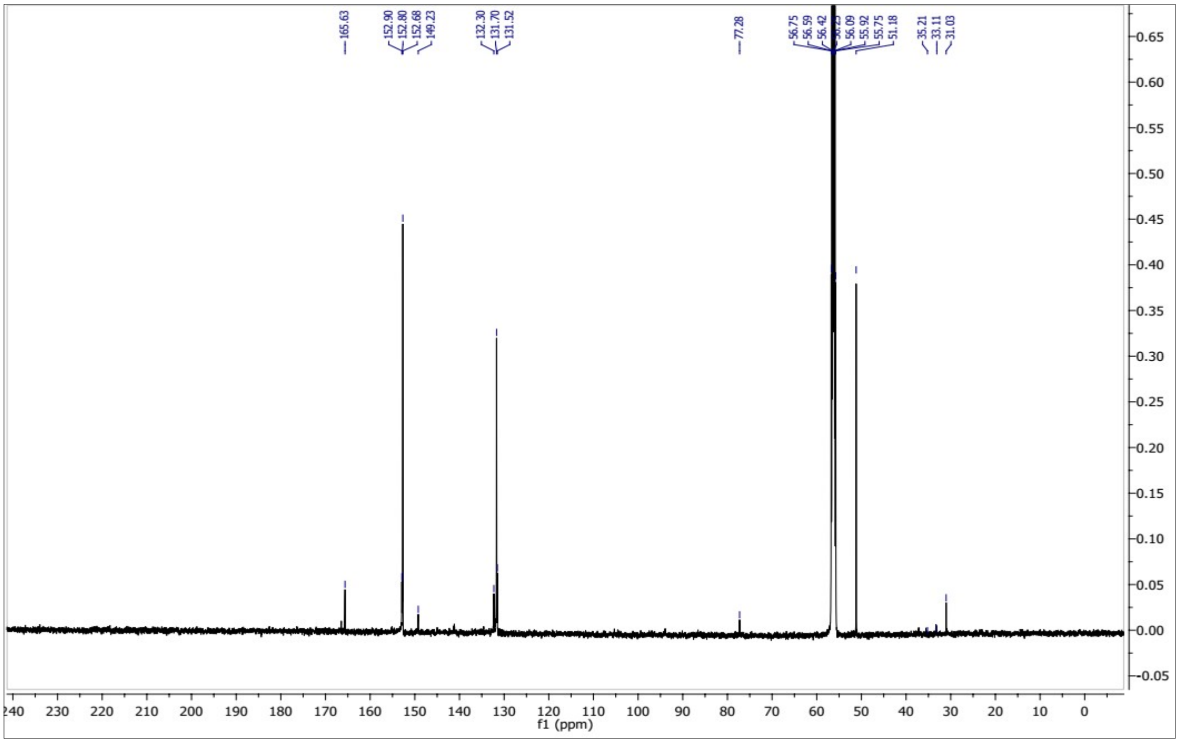

FTIR, ^{1}H-NMR (500 MHz, DMSO), and ^{13}C-NMR (125 MHz, DMSO) of compound 14.

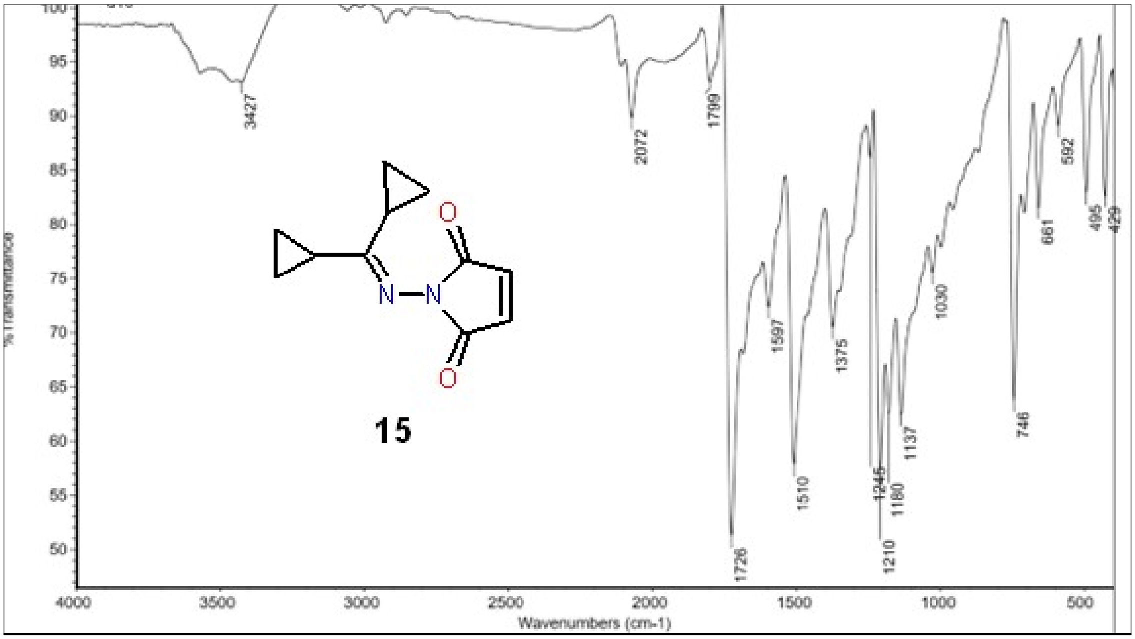

% Transmittance
Wavenumbers (cm-1)
3427
2072
1799
1726
1597
1510
1375
1245
1210
1180
1137
1030
746
661
592
495
429
15

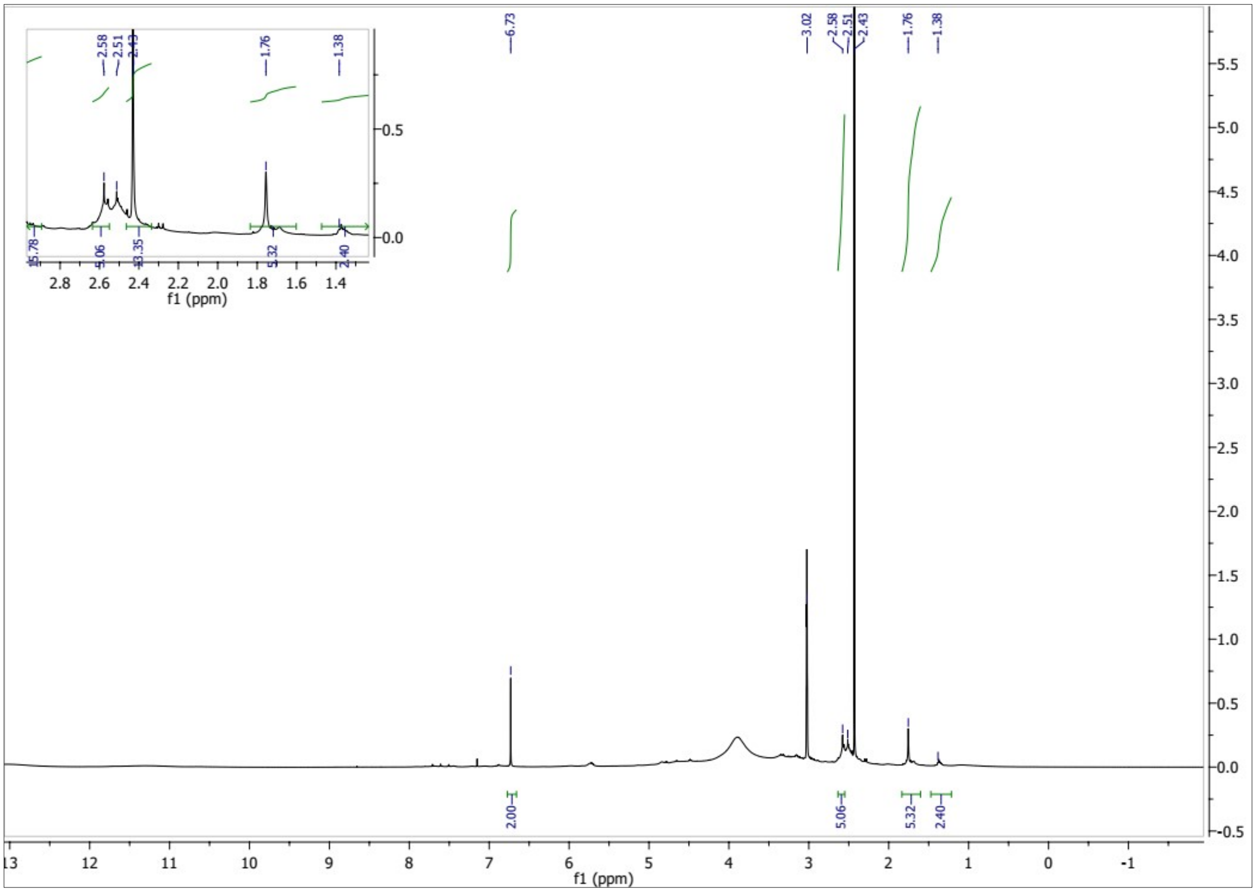

6.73
3.02
2.58
2.51
2.43
1.76
1.38
2.00
5.06
5.32
2.40
f1 (ppm)

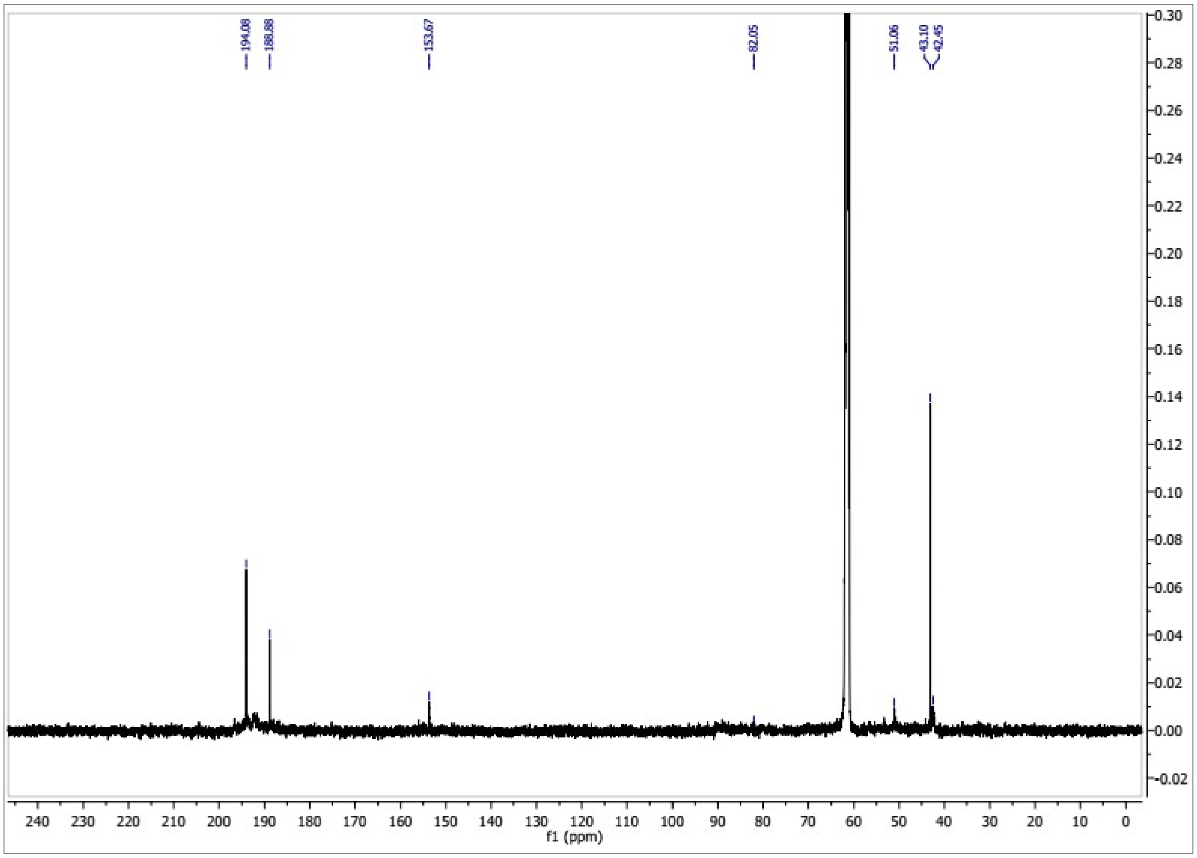

FTIR, ^{1}H-NMR (500 MHz, DMSO), and ^{13}C-NMR (125 MHz, DMSO) of compound 15.

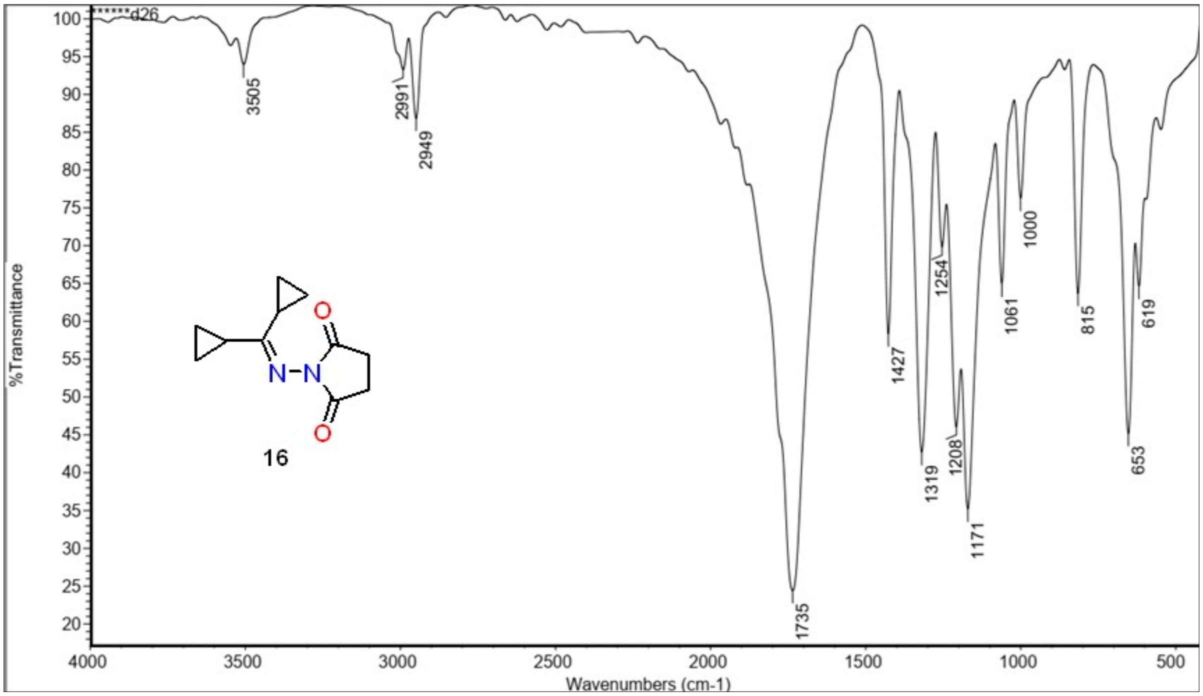

%Transmittance
Wavenumbers (cm-1)
3505
2991
2949
1735
1427
1319
1254
1208
1171
1061
1000
815
653
619
16

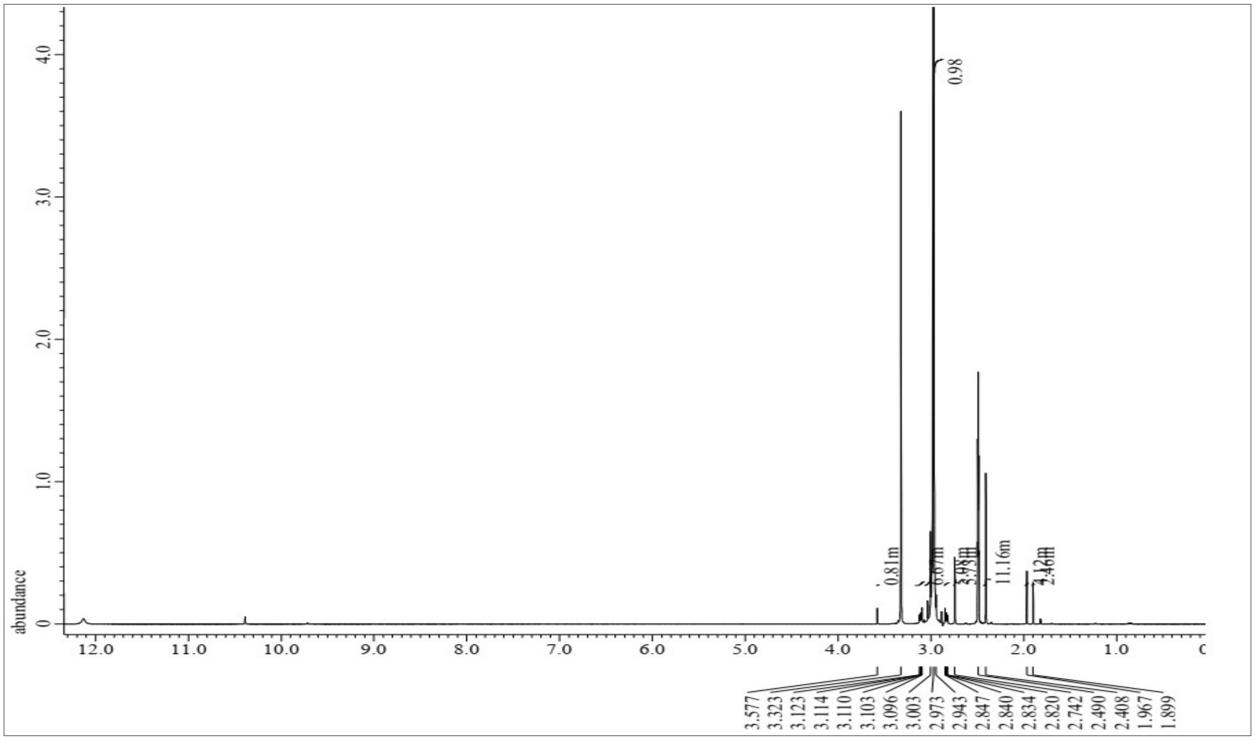

abundance
0.98
0.81m
11.16m
3.577
3.323
3.123
3.114
3.110
3.103
3.096
3.003
2.973
2.943
2.847
2.840
2.834
2.820
2.742
2.490
2.408
1.967
1.899

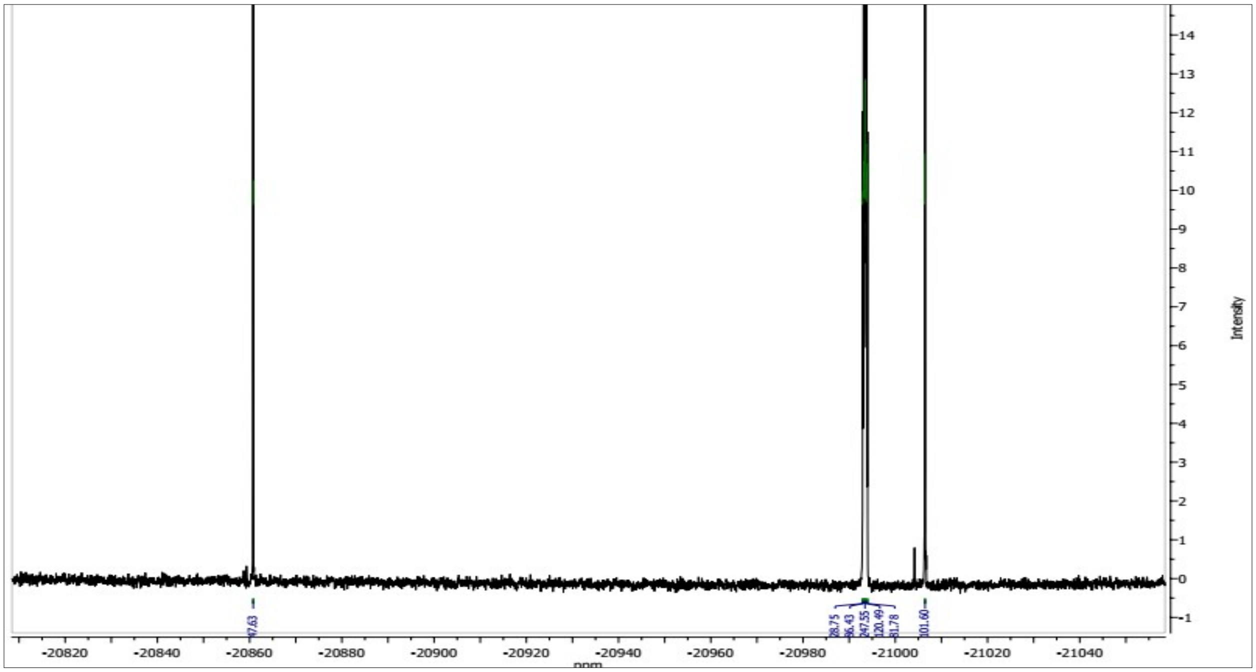

FTIR, ^{1}H-NMR (500 MHz, DMSO), and ^{13}C-NMR (125 MHz, DMSO) of compound 16.

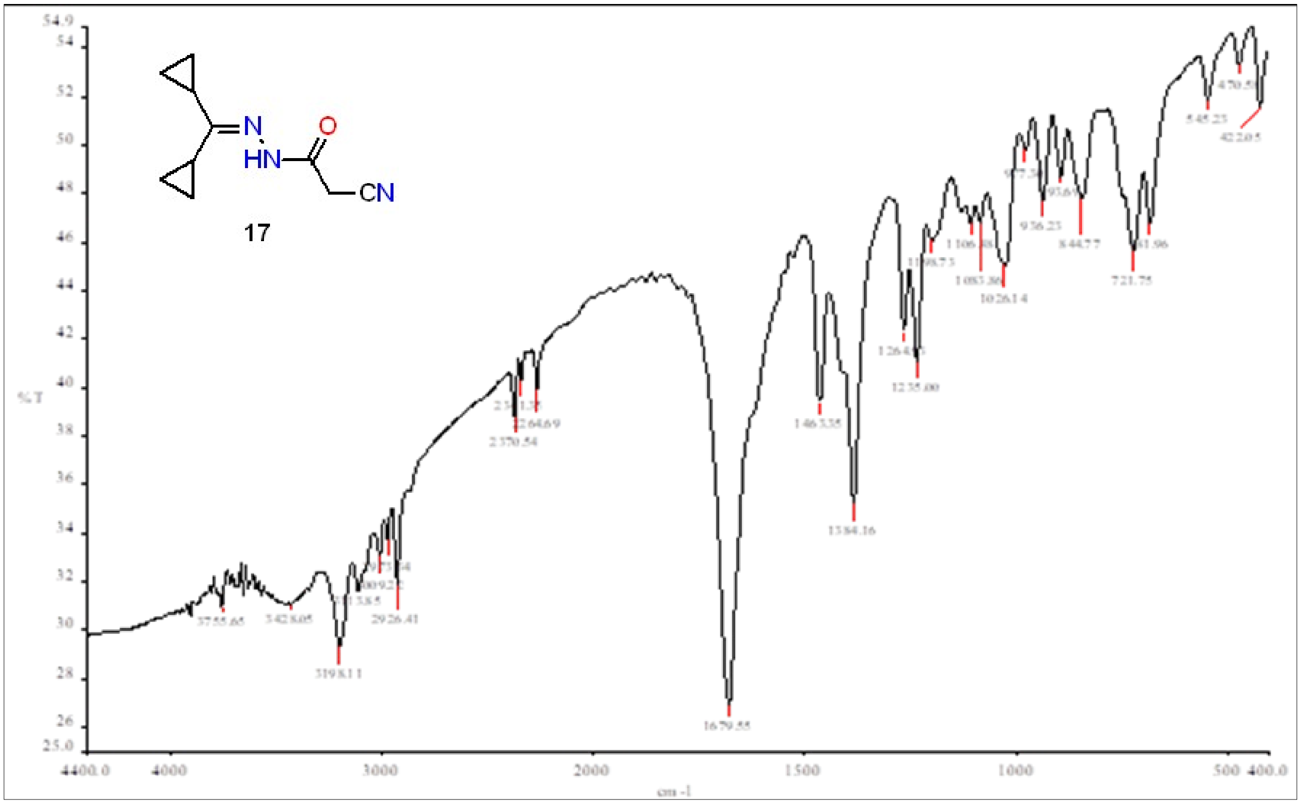

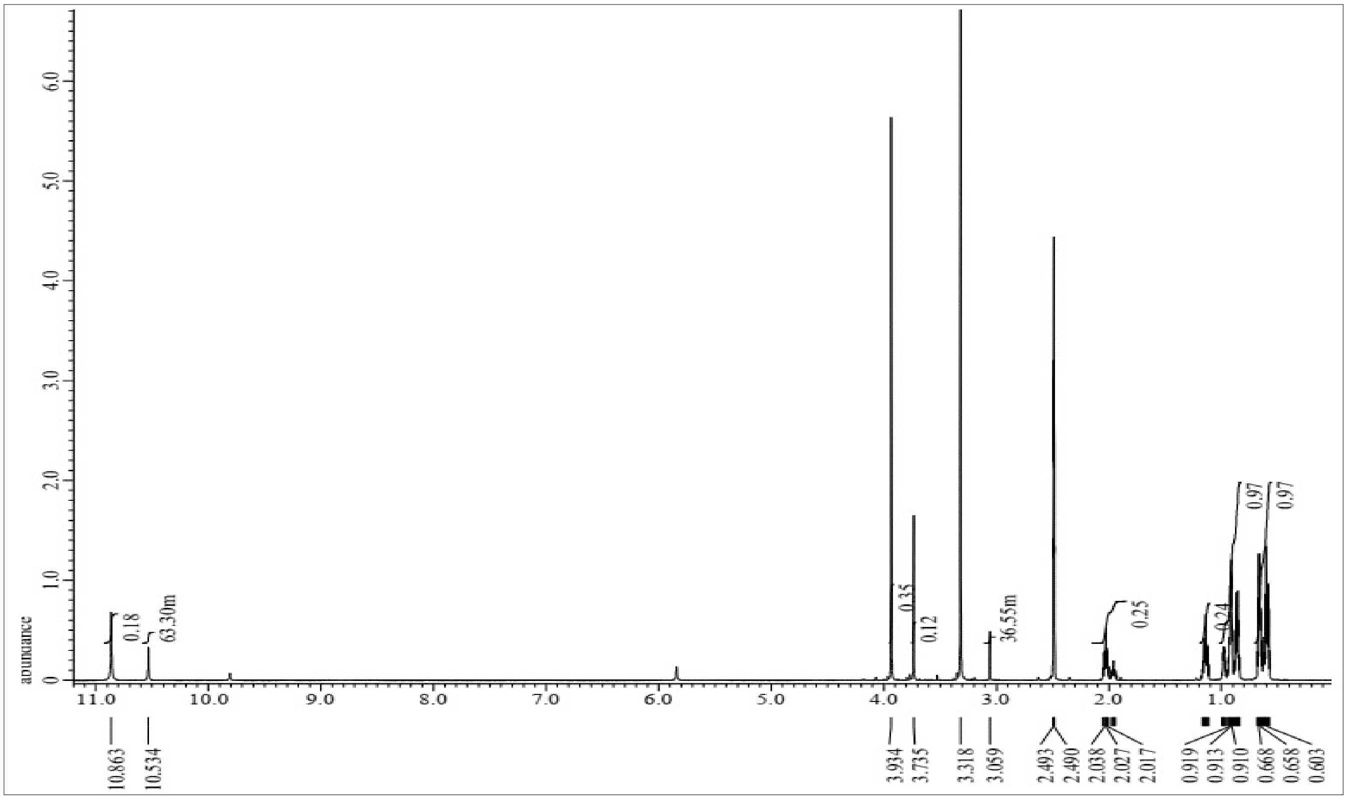

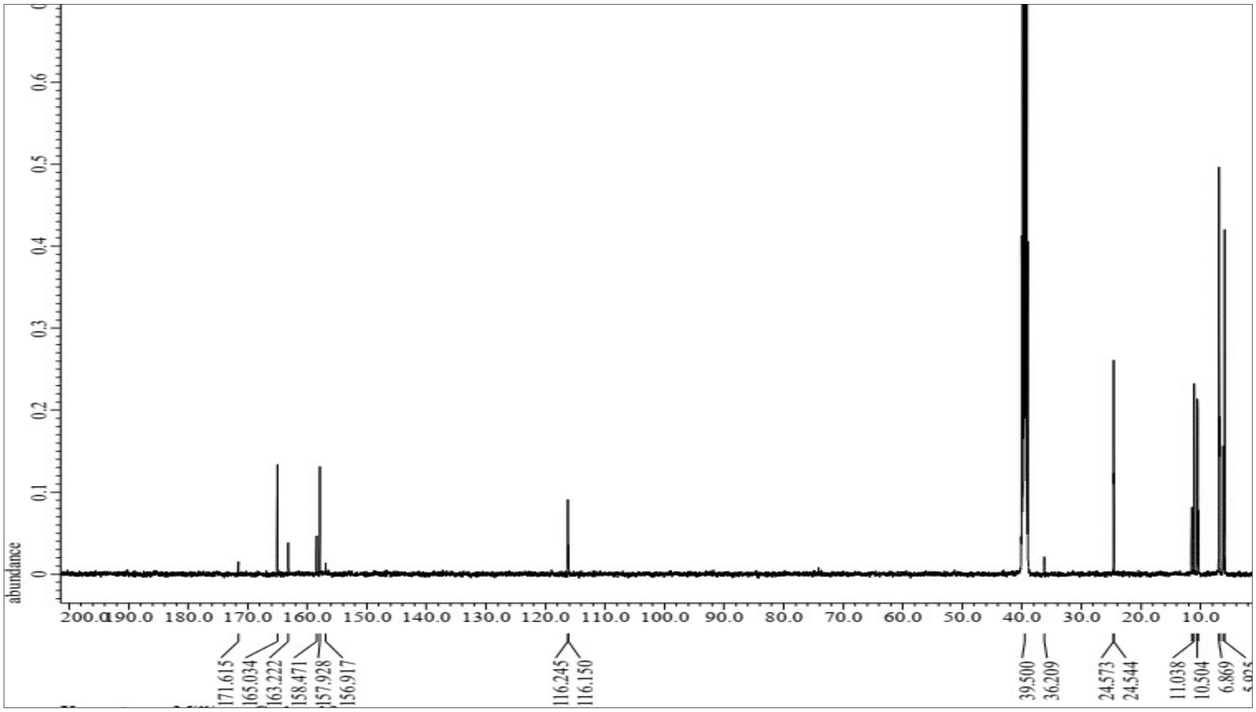

FTIR, ^{1}H-NMR (500 MHz, DMSO), and ^{13}C-NMR (125 MHz, DMSO) of compound 17.

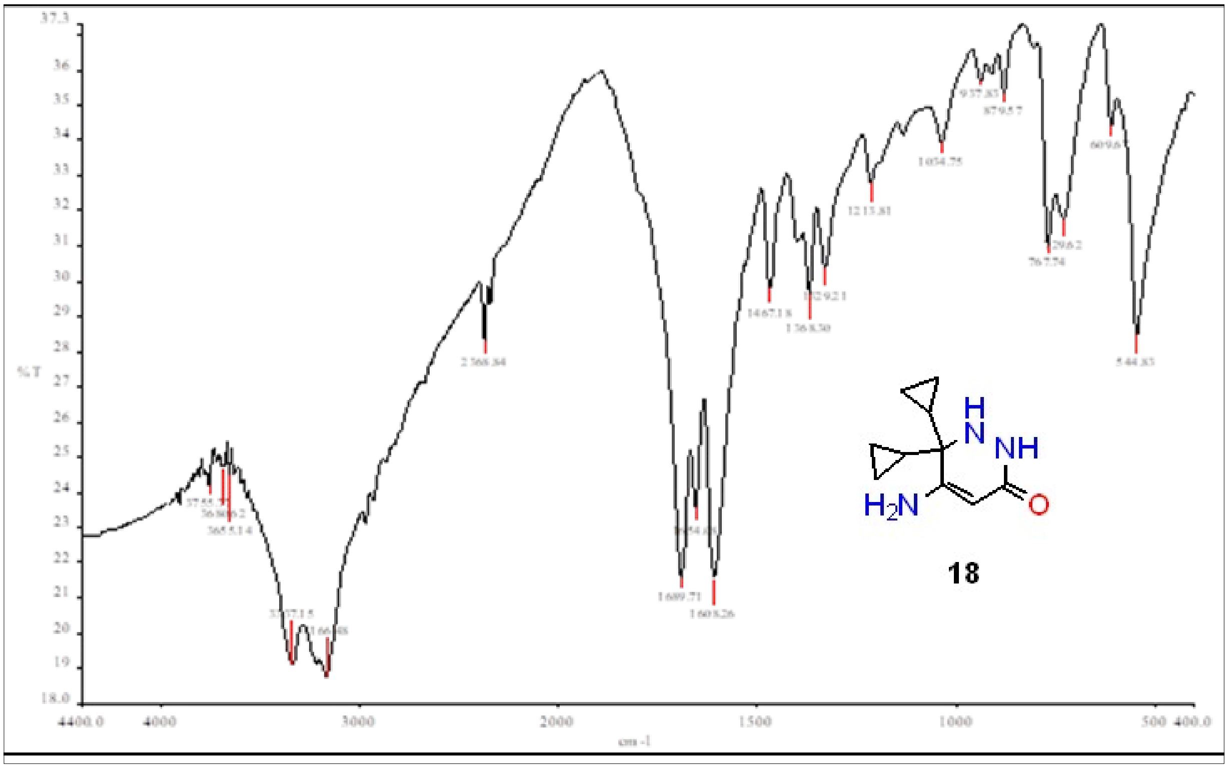

37.3
18.0
%T
4400.0
4000
3000
2000
1500
1000
500
400.0
cm-1
2368.84
1689.71
1608.26
1467.18
1368.30
1329.21
1213.81
1034.75
879.57
767.74
609.6
544.83
H
N
NH
H_2N
O
18

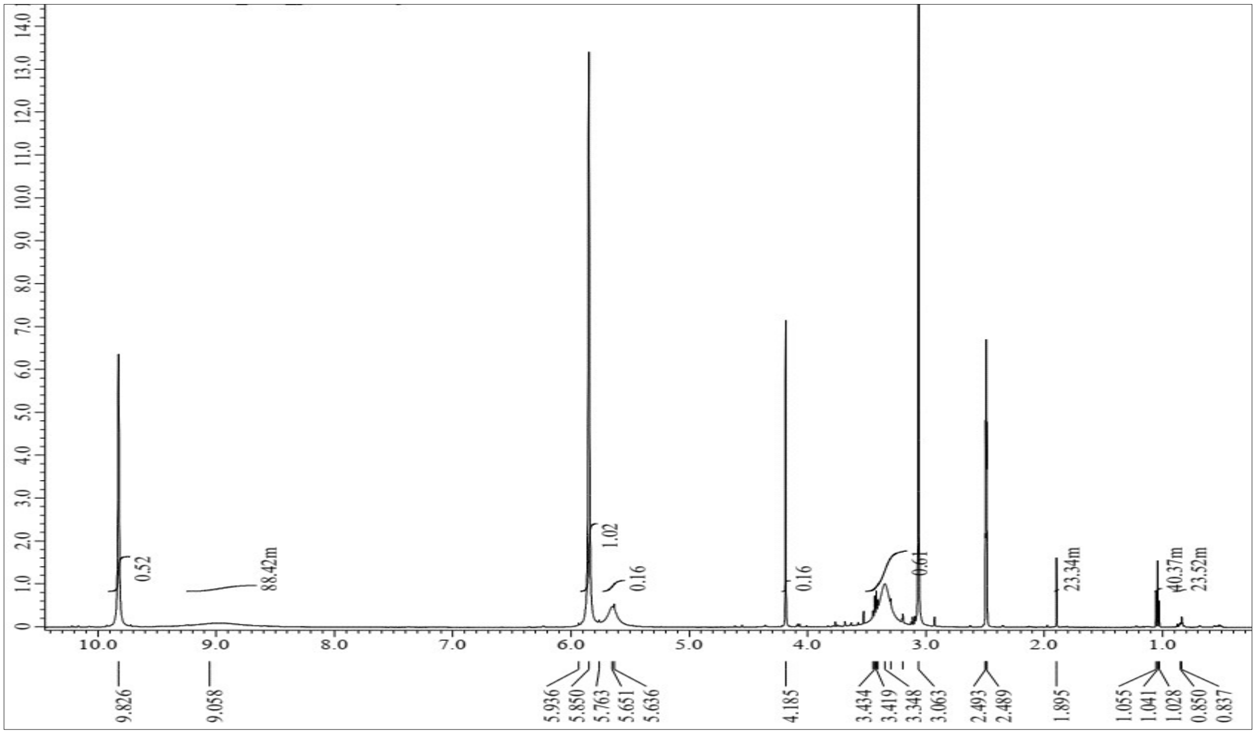

0.52
88.42m
1.02
0.16
0.16
0.61
23.34m
40.37m
23.52m
10.0
9.0
8.0
7.0
6.0
5.0
4.0
3.0
2.0
1.0
9.826
9.058
5.936
5.850
5.763
5.651
5.636
4.185
3.434
3.419
3.348
3.063
2.493
2.489
1.895
1.055
1.041
1.028
0.850
0.837

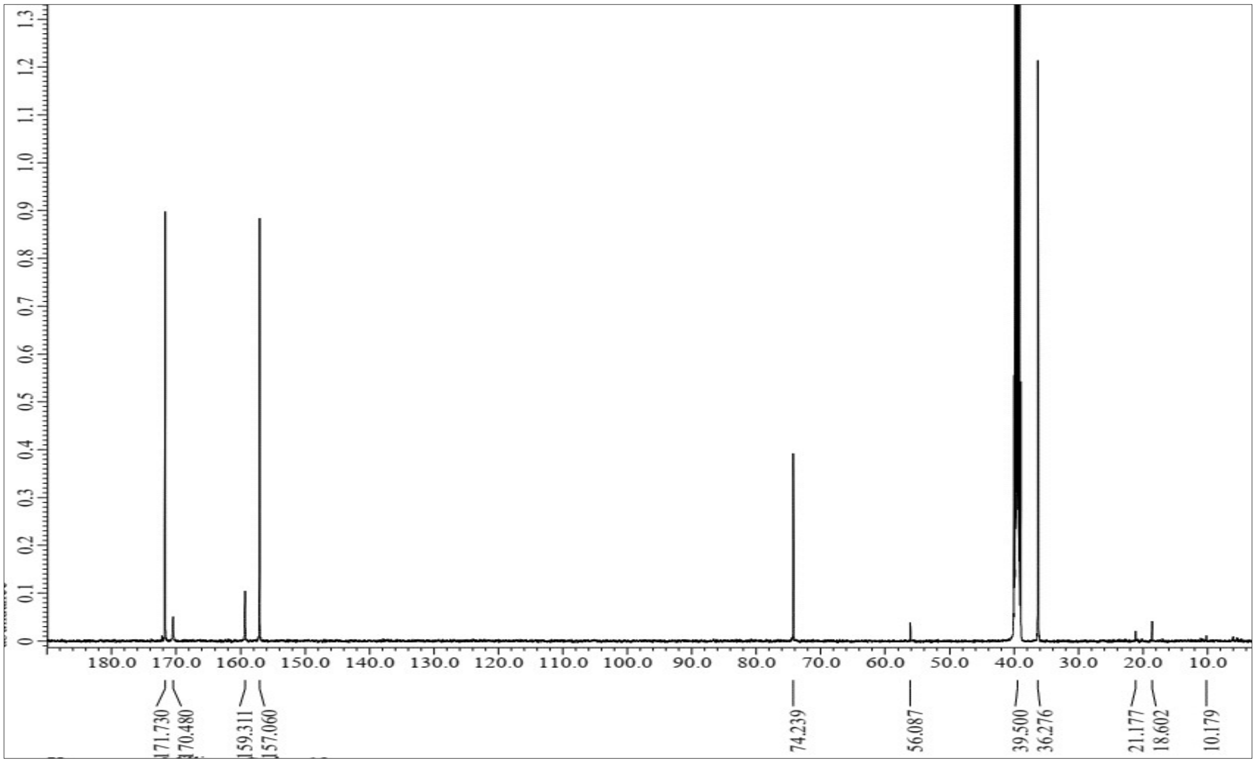

FTIR, ^{1}H-NMR (500 MHz, DMSO), and ^{13}C-NMR (125 MHz, DMSO) of compound 18.

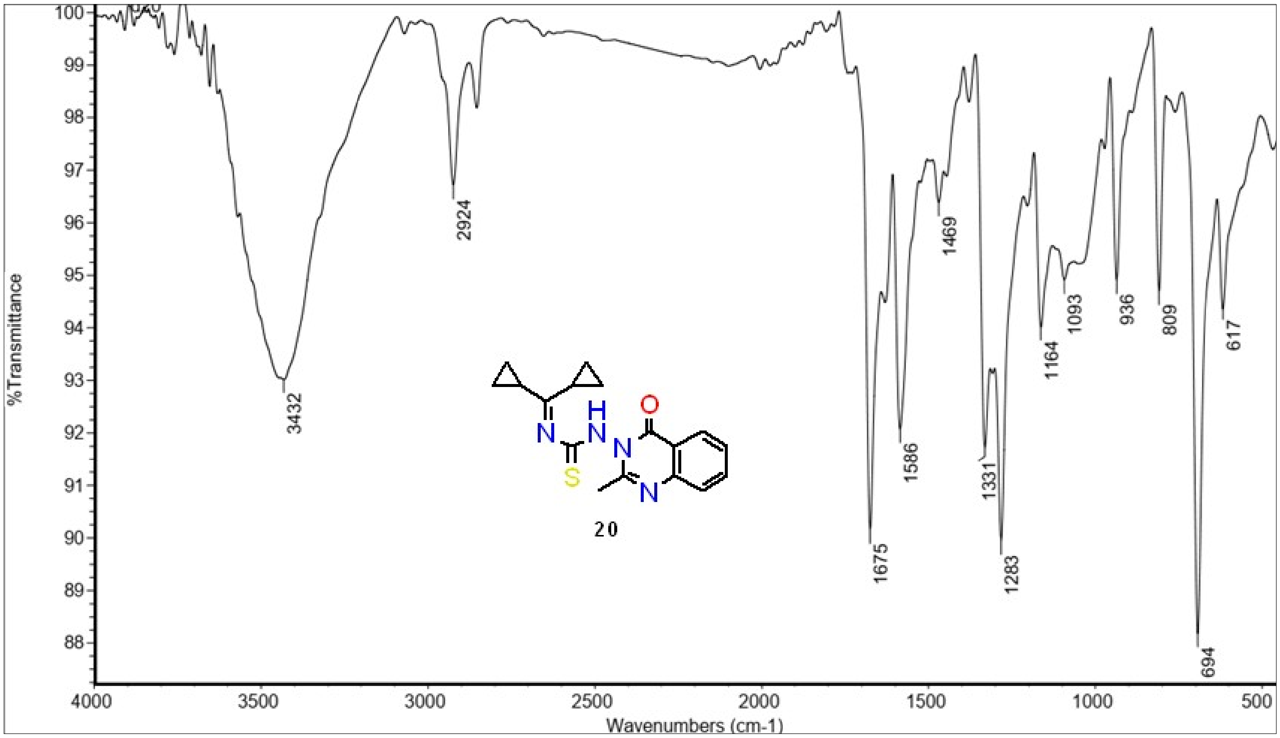

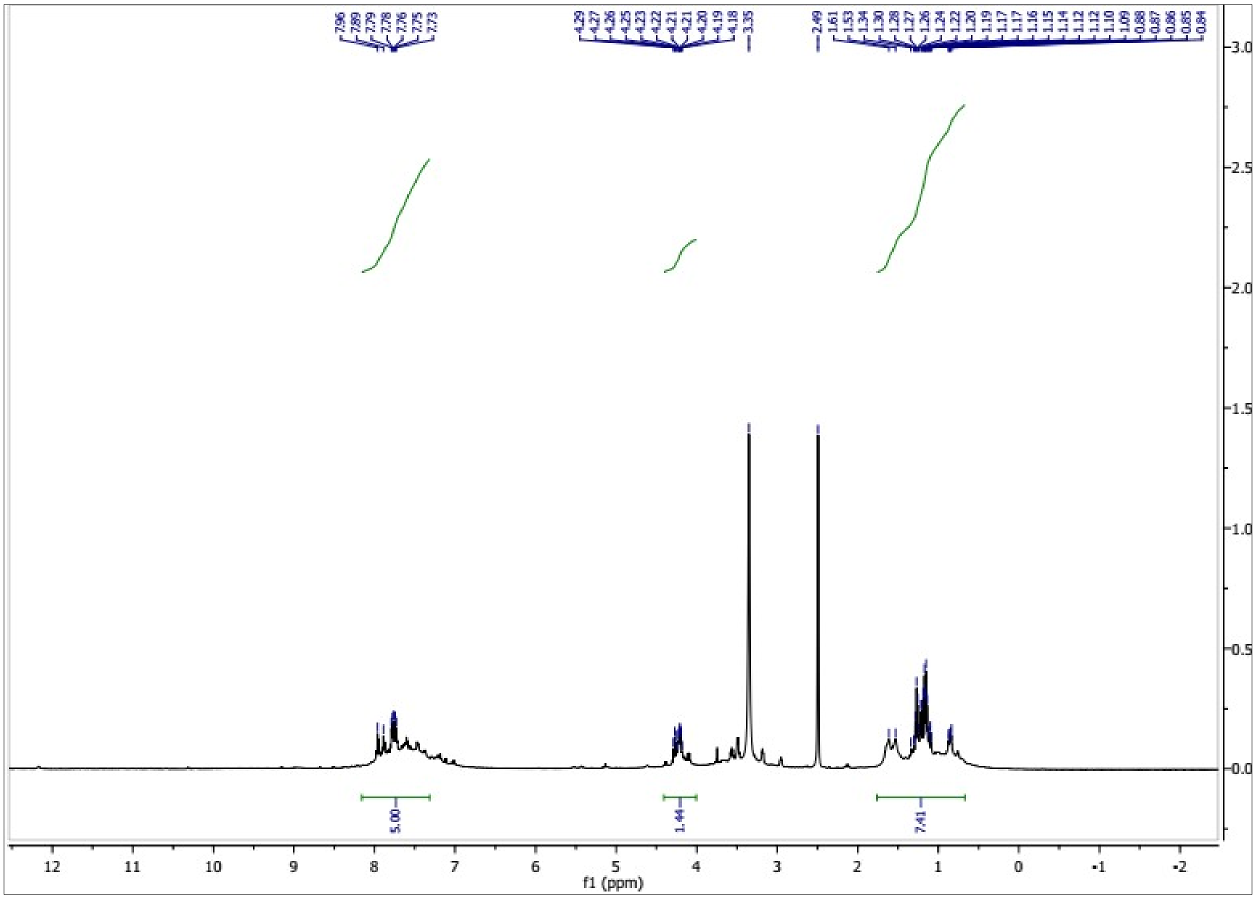

FTIR, and ^{1}H-NMR (500 MHz, DMSO) of compound 20

Table S1: Physicochemical properties and spectroscopy data of the synthesized compounds.

compound	Color Recrystallization solvent	Yield %	Mp C	IR (KBr, ν/cm^{-1}), ^{1}H, ^{13}C NMR (DMSO-d6, δ ppm). MS (EI): (m/z, %)
2	White needles (ethanol)	92%;	85	**IR : 3425, 3395 (NH2), 1604 (C=N).** **1H NMR: 0.6-1.07 (m, 8H, 4CH2), 2.25 (m, 4H, 2CH , NH2).** **13C-NMR: 5.6, 7.24, 9.36, 11.45 and 165.8.** **MS (EI): (m/z, %), 124 (M+-1, 1), 108 (100).**
3a	yellow needles (Ethanol)	78	164-6	**IR: 2967-2840 (CH aliphatic), 1659, 1600 (2C=N).** **MS (EI): (m/z, %), 241 (M^+-1, 9.08), 160 (100).**
3b	Yellow crystals (Ethanol)	92	>250	**IR: 2961-2830 (CH aliphatic), 1609 (2C=N). MS (EI): (m/z, %), 257 (M^+, 23)**
3c	buff needles (Ethanol)	81	104	**IR: 2927-2850 (CH aliphatic), 1656-1651 (2C=N).** **MS (EI): (m/z, %), 202 (M^+, 60).**
4	Dark red crystals (Ethanol)	88	>300	**IR: 3277(NH), 1723, 1613 (C=O, C=N).** **^{1}H NMR : 0.9-1.3 (m, 10H, H-aliph), 6.8-7.5 (m, 4H, H-Ar) and 10.9(s, 1H, NH).** **^{13}C-NMR: 7.36, 9.2, 13.13, 110.6, 111.12, 122.05, 127.8, 128.2, 134.4, 144.73, 145.19 and 163.4.** **MS (EI): (m/z, %), 253 (M^+, 100).**
5	Bright yellow crystals (Ethanol)	95	205-7	**IR: 3445(OH), 1622 (2 C=N).** **^{1}H NMR : 6.9-7.6(m, 8H, H-Ar), 8.9(s, 2H, CH=N), and 11.1(s, 2H, OH).**

				**MS (EI): (m/z, %), 240 (M^+, 17), and 185(100).**
6a	White crystals (Ethanol)	62	160-162	**IR (KBr, ν/cm^{-1}): 1601, 1685 (2 C=N), 3349 broad (NH_2, NH).**<br>**^{1}H NMR: 1.02 1.4 (m, 10H, H-aliphatic) and 3.8(s, 3H, OCH_3), 5.8 (s, 1H, CH), 6.7, 7.8 (m, 4H, H-Ar), 8.6 (s, 2H, NH_2) and 9.8 (s, 1H, NH).**<br>**MS (EI): (m/z, %), 268 (M^+-OCH_3, 6.6).**
7a	Yellow crystals (Ethanol)	75	238-240	**IR (KBr, ν/cm^{-1}): 3419, 3351 (2NH), 3259, 3190 (NH_2), 1679, 1613 (C=O, C=N).**<br>**^{1}H NMR: 1.0-1.055 (m, 10H, H-aliphatic), 6.8-7.3 (m, 4H, H-Ar), 10.5 (s, 1H, NH), 10.6 (s, 1H, NH) and 11.1 (s, 2H, NH_2) .**<br>**^{13}C-NMR: 10-20, 109.9, 111.03, 117.4, 119.98,162.7, 178.66 and 181.13.**<br>**MS (EI): (m/z, %), 313 (M^++2, 8.4).**
6b	Brown crystals (Ethanol)	62	>250°c	**IR: 3469 (NH), 3196, 3351(CH aromatic), 1639, 1605 (C=N).**<br>**^{1}H NMR: 1.0-1.055 (m, 10H, H-aliphatic), 3.1(s, 3H, OCH_3), 6.8-7.3 (m, 4H, H-Ar), 8.5 (s, 1H, NH).**<br>**MS (EI): (m/z, %), 313 (M^++2, 8.4)**
7b	Bage crystals (Ethanol)	62	262-264	**IR: 3438, 3238 (2NH), 1691, 1622 (C=O, C=N).**<br>**^{1}H NMR: 0.9-2.1 (m, 10H, H-aliphatic), 10.11 (s, 1H, NH), 11.2 (s, 1H, NH) and 6.7-**

				**8.2 (m, 9H, H-Ar).** **^{13}C-NMR: 17.8, 18.9, 23.3, 23.5, 32.14, 85.3, 127.7, 128.6, 130.2, 140.5, 141.8, 142.0, 146.5, 164.7, 165.2, 167.2, and 169.7.** **MS (EI): (m/z, %) 360 (M^+- 1, 15.1).**
8a	Faint yellow crystals (Methanol)	95	226-8	**IR: 3443, 3320 (NH_2), 3173, 3137 (2CH=N), 1650, 1613 (C=N).** **^{1}H NMR: 6.7-8.0 (m, 8H, H-Ar), 8.3 (s, 1H, CH=N), 9.8(s, 1H, NH_2) and 11.3 (s, 1H, OH).** **^{13}C-NMR: 116, 119.34, 120.37, 126.7, 131.17, 139.7, 156.4 and 177.6.** **MS (EI): (m/z, %), 297 (M^+, 2.5), 195(100).**
8b	Yellow crystals (methanol)	55	198-200	**IR: 1689,1623 (2 C=N).** **^{1}H NMR: 6.9-7.6 (m, 8H, H-Ar), 8.99 (s, H, CH=N) and 11.1(s, 2H, 2OH).** **MS (EI): (m/z, %), 356 (M^+, 20).**
10a	Brown crystals (Ethanol)	62	152-154	**IR: 1704, 1643, 1600 (C=O, 2C=N).** **^{1}H NMR: 1.9-2.0 (m, 13H, H-aliphatic, CH_3) and 6.9-8.3 (m, 6H, H-Ar).** **^{13}C-NMR: 20.5, 23.8, 31.4,32.6, 34.7, 72.25, 146.9, 149.8, 150.8, 151.1, 152.5, 153.09, 154.1, 154.9, 155.18, 155.53, 184.8, and 18873.** **MS (EI): (m/z, %), 289 (M^+- 4, 10.6).**
10b	Yellow crystals (acetic acid)	74	158-160	**IR: 1704,116455 (C=O,2 C=N).** **^{1}H NMR: 0.8-2.1 (m, 10H, H-aliphattic), 1.8 (s,3H, CH_3) and 7.4-8.3 (m, 11H, H-Ar,**

				**CH=C).** **^{13}C-NMR (DMSO-d6): δ ppm 10-20, 14.02, 20.5, 21.06, 125.1-133.7 Ar-C, 163.06, 165.8, 167.3, 169.12, and 172.03.** **MS (EI): (m/z, %), 352 (M^+, 1.9).**
11	Faint yellow needles (Ethanol)	82	230-232	**IR: 1675, 1660 (C=O, C=N).** **^{1}H NMR: 1.02-2.1 (m, 13H, H-aliphatic, CH_3) and 7.9-8.2 (m, 4H, H-Ar).** **^{13}C-NMR: 10.55-70.5, 148.1, 154.37, 155.9, and 203.8.** **MS (EI): (m/z, %), 267 (M^+, 3.6).**
13	Buff crystals (Methanol)	89	185-186	**IR: 3450 (2NH), 3428 (NH), 1687, 1603 (2C=O).** **^{1}H NMR: 1.2-1.5 (m, 10H, H-aliphatic), 6.1(s, 1H, NH), 7.7-8.01 (m, 1H, H-Ar), and 11.52(s, 1H, NH).** **^{13}C-NMR: 16.6-77, 18.1, 20.1, 20.9, 110.99, 125.3, 125.93, 130.56, 131.87, 134.47, 159.48, 181,24 and184.73.** **MS (EI): (m/z, %), 296 (M^+, 1.5).**
14	White crystals (Acetic acid)	72	>250	**IR: 3459(OH), 1746, 1660, 1605 (2C=O, C=N).^{1}H NMR: 0.6-1.8 (m, 10H, H-aliphatic), 7.8-8.1 (m, 5H, H-Ar) 77.9 (s, 1H, NH) and 11.5 (s, 1H, COOH).** **^{13}C-NMR: 18.6, 77.28, 131.5, 131.7, 132.3, 149.2, 152.6, 152.8, 152.9, and 165.6.** **MS (EI): (m/z, %), 272 (M^+, 25).**

15	Brown crystals (Methanol)	55	>260°	**IR: 1799, 1728, 1634 (2C=O, C=N).**<br>**^{1}H NMR: 0.8-2.1 (m, 10H, H-aliphatic), 6.7 (dd, 2H, H-Ar).**<br>**^{13}C-NMR: 34.2-82.8, 175.01, 153.65, 188.85 and 194.0.**<br>**MS (EI): (m/z, %), 204 (M^+, 56).**
16	White needles (DMF)	82	245-8	**IR: 1735 (2C=O).**<br>**^{1}H-NMR (DMSO-d6) δ ppm 1.8-3.5 (m, 14H, H-aliph).**<br>**^{13}C-NMR: 26.3-72, 172.0 and 172.2.**<br>**MS (EI): (m/z, %), 206 (M^+, 62).**
17	White crystals (Ethanol)	65	125-126	**IR (KBr, ν/cm^{-1}):3198 (NH), 2264(—C≡N), 1679 (C=N).**<br>**^{1}H NMR : 0.6-2.03 (m, 10H, H-aliphatic), 3.9 (s, 2H, CH_2) and 10.86 (s, 1H, NH).**<br>**^{13}C-NMR (DMSO-d6): δ ppm 5.92, 6.86, 10.5, 11.03, 24.57, 36.2, 116.24, 157.92, 163.2, 165.3, and 171.6.**<br>**MS (EI): (m/z, %), 191 (M^+, 13), 123(100).**
18	Brown pellets (Ethanol)	33	210-11	**IR: 3337(NH), 3166, 3100 (NH_2), 1689, 1654 (C=O, C=N).**<br>**^{1}H NMR : 0.8-1.8 (m, 10H, H-aliphatic), 4.1(s, 1H, CH-N), 5.6 (s, 1H, NH), 5.8 (S, 1H, H-Ar) and 9.8(s, 2H, NH_2).**<br>**^{13}C-NMR: 10.17, 18.6, 21.17, 36.27, 56.08, 74.23, 157.0, 159.3 and 171.7.**<br>**MS (EI): (m/z, %), 193 (M^+, 1.5), 69 (100).**

20	Brown needles (Ethanol)	45	135-2°c	**IR: 3432 (NH), 1675, 1660 (C=O, C=N).**<br>**^{1}H NMR: 0.8 -1.8 (m, 13H, H-aliphatic, CH_3), 7.7-7.9 (m, 4H, H-Ar) and 8.2 (s, 1H, NH).**<br>**MS (EI): (m/z, %), 326 (M^+, 100).**

Figure S2. Caspase 3 assay.

Compound 16

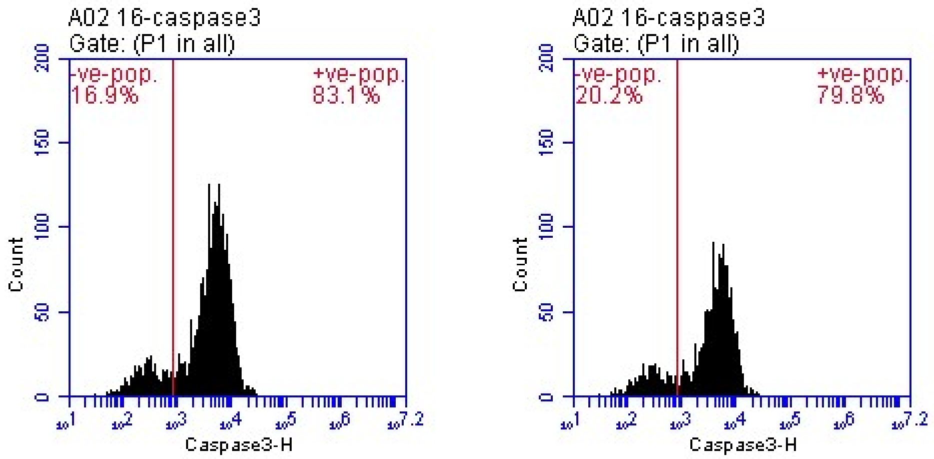

Compound 2

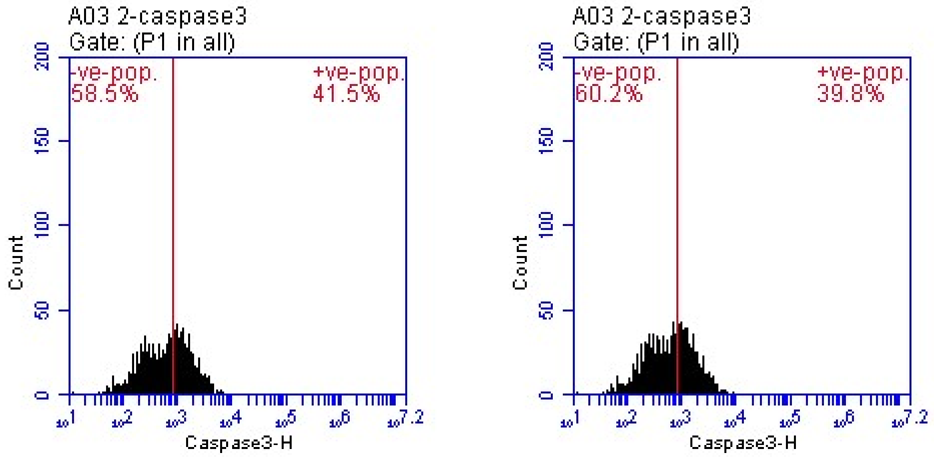

Compound 18

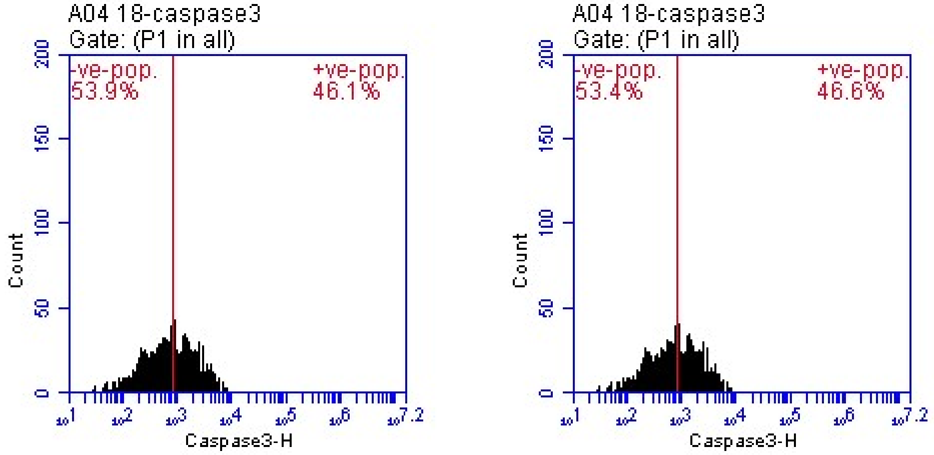

Media (No treatment)

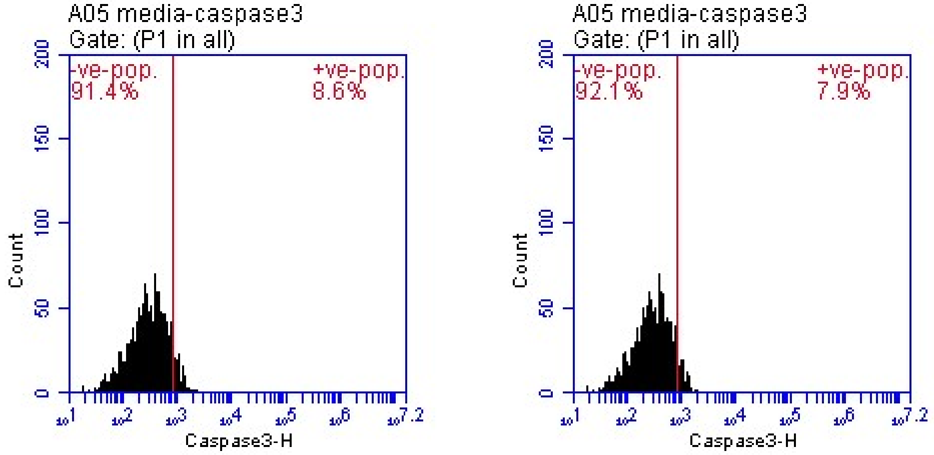

Compound 13

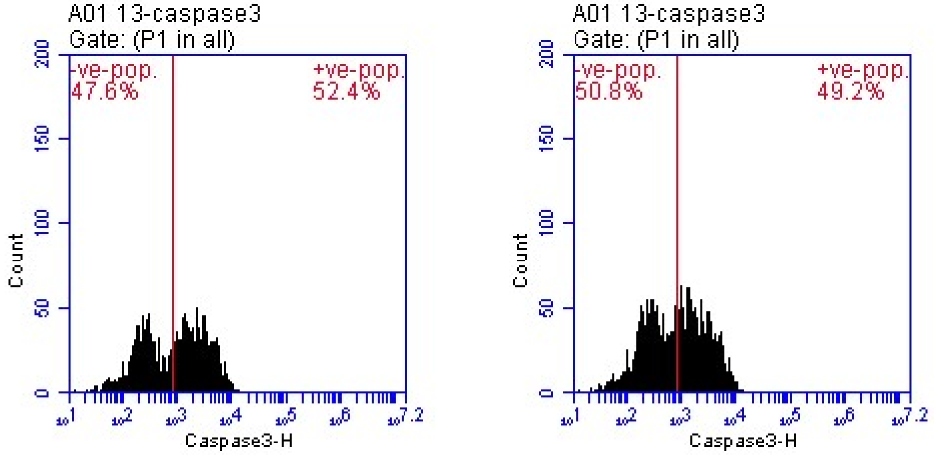

Sorafenib

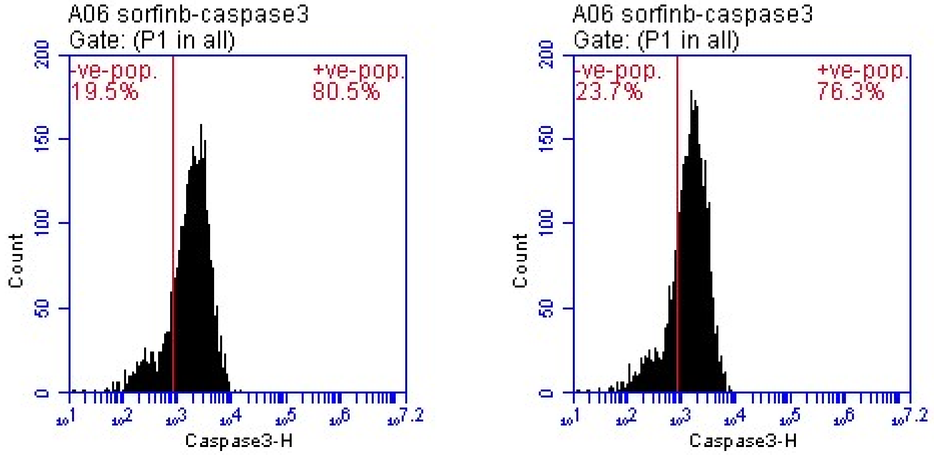

Compound 8a

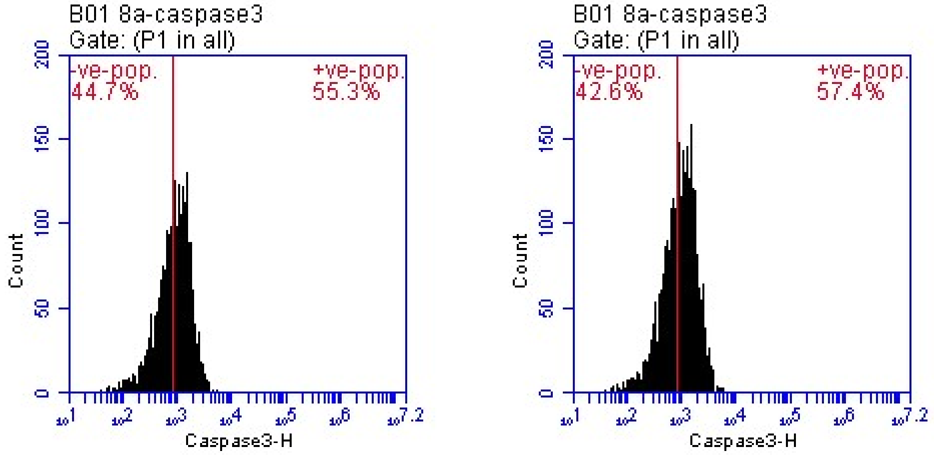

Compound 20

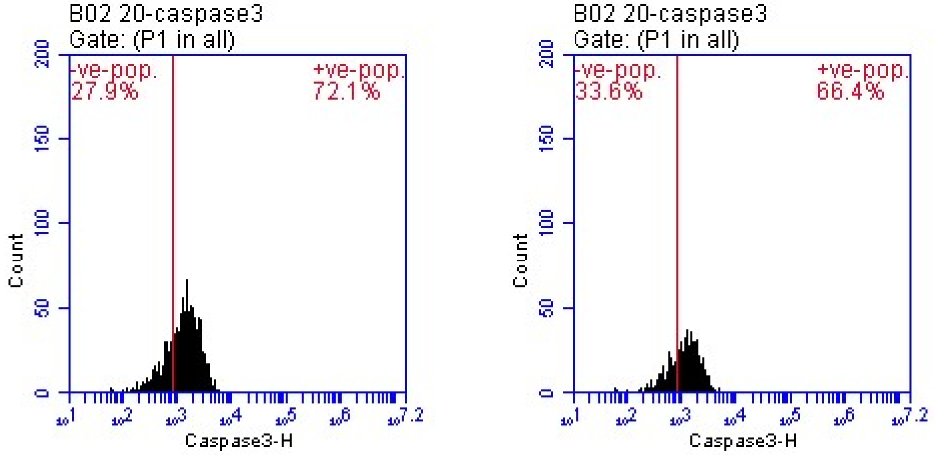

Compound 4

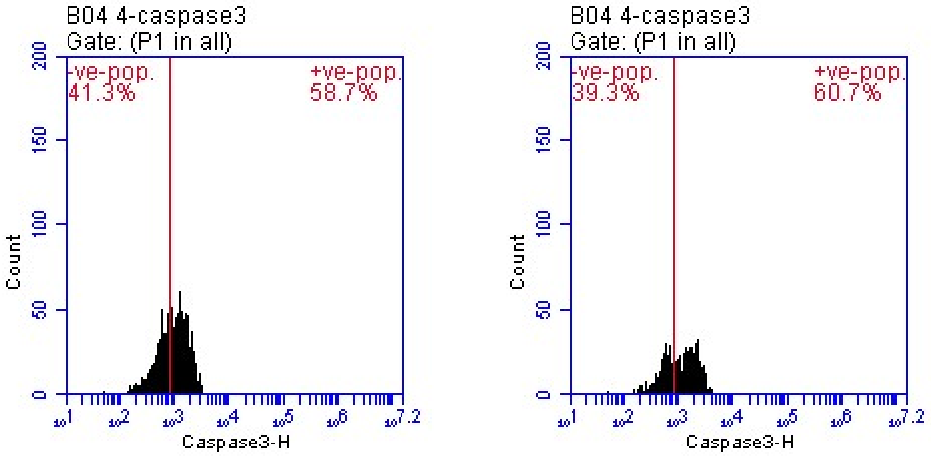
B04 4-caspase3
Gate: (P1 in all)
-ve-pop.
41.3%
+ve-pop.
58.7%
Count
Caspase3-H
B04 4-caspase3
Gate: (P1 in all)
-ve-pop.
39.3%
+ve-pop.
60.7%
Count
Caspase3-H

Figure S3. Cell cycle analysis.

Media (No treatment)

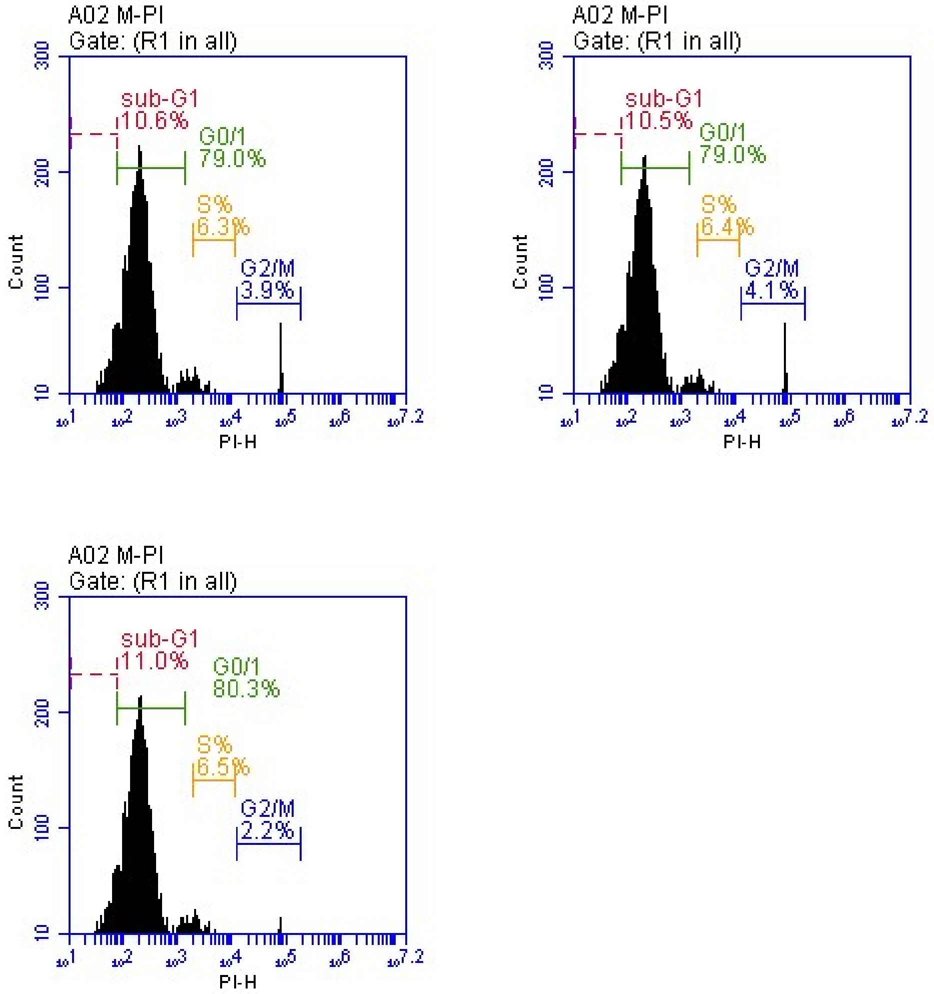

Sorafenib

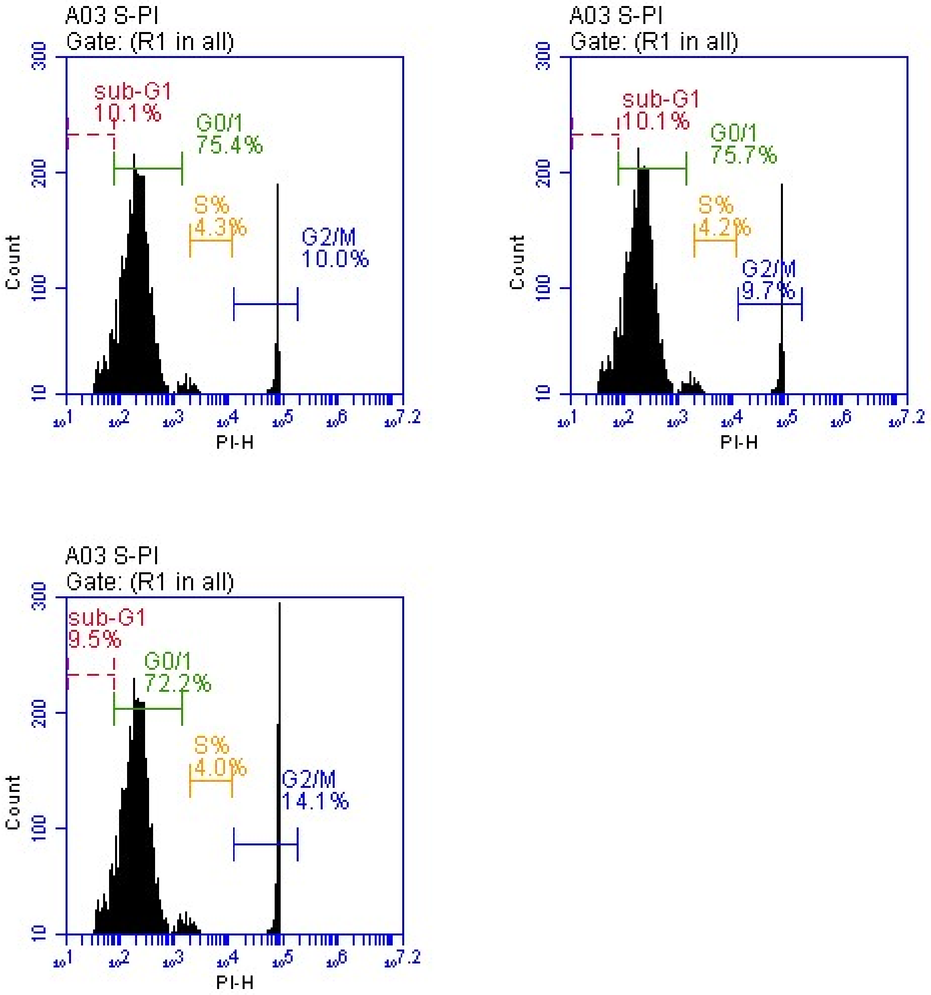
A03 S-PI
Gate: (R1 in all)
sub-G1
10.1%
G0/1
75.4%
S%
4.3%
G2/M
10.0%
Count
PI-H
A03 S-PI
Gate: (R1 in all)
sub-G1
10.1%
G0/1
75.7%
S%
4.2%
G2/M
9.7%
Count
PI-H
A03 S-PI
Gate: (R1 in all)
sub-G1
9.5%
G0/1
72.2%
S%
4.0%
G2/M
14.1%
Count
PI-H

Compound 16

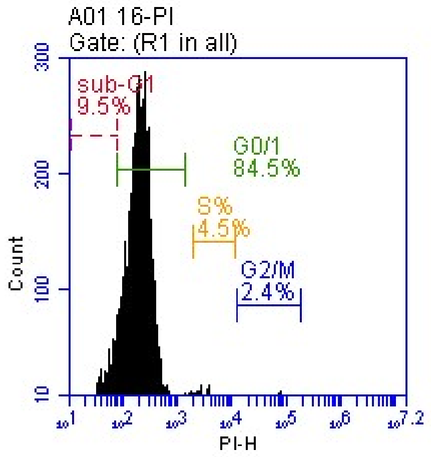
A01 16-PI
Gate: (R1 in all)
sub-G1
9.5%
G0/1
84.5%
S%
4.5%
G2/M
2.4%
Count
PI-H

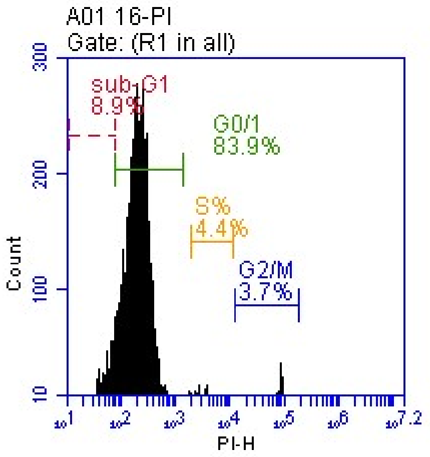
A01 16-PI
Gate: (R1 in all)
sub-G1
8.9%
G0/1
83.9%
S%
4.4%
G2/M
3.7%
Count
PI-H

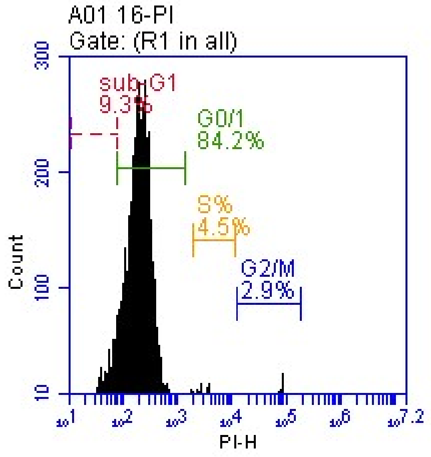
A01 16-PI
Gate: (R1 in all)
sub-G1
9.3%
G0/1
84.2%
S%
4.5%
G2/M
2.9%
Count
PI-H
